# Pushing the Limits of Characterising a Weak Halogen Bond in Solution

**DOI:** 10.1002/chem.202103559

**Published:** 2021-12-13

**Authors:** Stefan Peintner, Máté Erdélyi

**Affiliations:** ^1^ Department of Chemistry – BMC Uppsala University SE-751 23 Uppsala Sweden

**Keywords:** conformation, ensemble analysis, halogen bond, NAMFIS, NMR spectroscopy, RDC

## Abstract

Detection and characterisation of very weak, non‐covalent interactions in solution is inherently challenging. Low affinity, short complex lifetime and a constant battle against entropy brings even the most sensitive spectroscopic methods to their knees. Herein we introduce a strategy for the accurate experimental description of weak chemical forces in solution. Its scope is demonstrated by the detailed geometric and thermodynamic characterisation of the weak halogen bond of a non‐fluorinated aryl iodide and an ether oxygen (0.6 kJ mol^−1^). Our approach makes use of the entropic advantage of studying a weak force intramolecularly, embedded into a cooperatively folding system, and of the combined use of NOE‐ and RDC‐based ensemble analyses to accurately describe the orientation of the donor and acceptor sites. Thermodynamic constants (Δ*G*, Δ*H* and Δ*S*), describing the specific interaction, were derived from variable temperature chemical shift analysis. We present a methodology for the experimental investigation of remarkably weak halogen bonds and other related weak forces in solution, paving the way for their improved understanding and strategic use in chemistry and biology.

## Graphical abstract

**Very weak halogen bonds** are excessively difficult to characterise in solution. We present an approach for the detailed NMR spectroscopic description of very weak interactions, providing the solution ensemble of the flexible model compound, the accurate geometry of the binding sites and the corresponding thermodynamic parameters. Here NOE abbreviates nuclear Overhauser effect, RDC corresponds to residual dipolar coupling, and Δ*T* indicates variable temperature experiments.

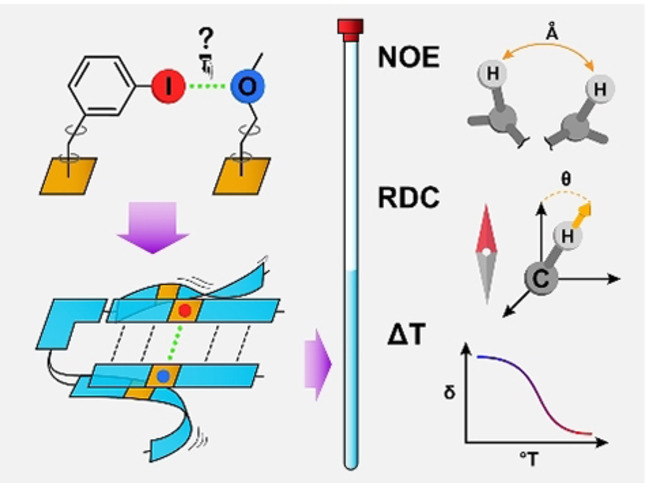

## Introduction

A halogen bond (XB) is the attractive interaction of a polarized halogen and a Lewis base.^[1]^ It resembles the hydrogen bond^[2]^ and has recently gained applications, for instance, in crystal engineering,^[3]^ materials design,[^3d^, ^4^] supramolecular chemistry,^[5]^ organic synthesis^[6]^ also including catalysis,^[7]^ structural biology^[8]^ and drug discovery.^[9]^ Being a very weak interaction, it has primarily been studied in the solid‐state and in silico,^[10]^ whereas its characterisation in disordered phases,^[11]^ especially in polar solutions^[12]^ remains a challenge.^[13]^ Most solution studies so far have assessed the easier to detect strong complexes, in which a large electron depleted area on the halogen bond donor has been induced either by perfluorination of nearby carbon atoms, or by oxidation of the halogen to halogen(I)^[6c]^ or halogen(III).^[14]^ Only scarce examples of molecular systems of direct relevance for pharmaceutical applications in polar solvents have yet been presented.[^8^, ^15^]

Intramolecular assessment of weak interactions offers an entropic advantage, and accordingly, the halogen bond of a perfluorinated donor could be assessed even in polar solvents, upon incorporation into an intramolecular model system.^[16]^ Furthermore, 4‐halo‐substituted phenylalanine was engineered into T4 lysozyme and its intramolecular halogen bond to a carbonyl oxygen was observed by X‐ray crystallography, and the existence of the bond further corroborated by solution calorimetry.^[17]^ A very weak Cl⋅⋅⋅O bond of a non‐fluorinated halogen bond donor has been characterized in DMSO, when studied in a cooperatively folding cyclic decapeptide.^[18]^ However, the chemical instability of this system impeded the investigation of bromine or iodine‐centered halogen bonds. Moreover, the cyclic nature of the system prevented the accurate experimental thermodynamic characterisation of the interaction. Thereto, the precise orientation of the halogen bond donor and acceptor sites could not be precisely described experimentally, only allowing the conclusion that the spectroscopic data was compatible with the formation of a halogen bond.

Herein, we present a strategy for the remarkably accurate spectroscopic characterisation of weak interactions in solution. Making use of a non‐cyclic, cooperatively folding backbone and the entropic advantage of an intramolecular setting, the method allows the thorough analysis of one specific weak interaction. We introduce the use of orienting media to the research field of halogen bonding, for the detection of bond vectors upon observation of residual dipolar couplings (RDCs). This allows the experimental detection of the orientation of halogen bond donor and acceptor sites, and thereby offers a direct evidence for the formation of a halogen bond in solution, without enforcement of the bonding geometry. The combined use of NOE‐based ensemble analysis and RDCs of a highly flexible molecular system is novel. The scope of this strategy is demonstrated by the detailed characterisation of an iodine‐centered, weak halogen bond in solution that has previously been inaccessible, being too weak to be detected by current techniques. Providing a new strategy to study the weakest interactions in solutions, we expect to facilitate future developments and applications of halogen bonding in, for example, medicinal chemistry and catalysis.

## Results and Discussion

### Design

**Figure 1:**
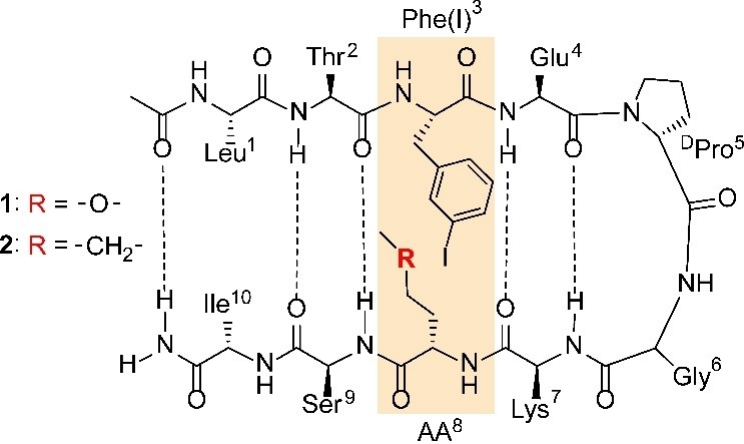
Compounds **1** and **2**, depicted as folded, antiparallel β‐hairpin. The central ^D^Pro‐Gly type II’ β‐turn reverses the backbone promoting the formation of a cross‐strand hydrogen bond network. In **1**, a cross‐strand halogen bond may form between the iodine of Phe(I)^3^ and oxygen of Hse(Me)^8^ (highlighted with yellow). Compound **2** is used as a reference that resembles **1** yet cannot form an interstrand halogen bond as the Lewis basic acceptor oxygen in amino acid 8 is replaced by a non‐Lewis basic methylene group. For **1** amino acid 8 is homo‐methyl‐serine, whereas for **2** it is a norleucine.

**Figure 2:**
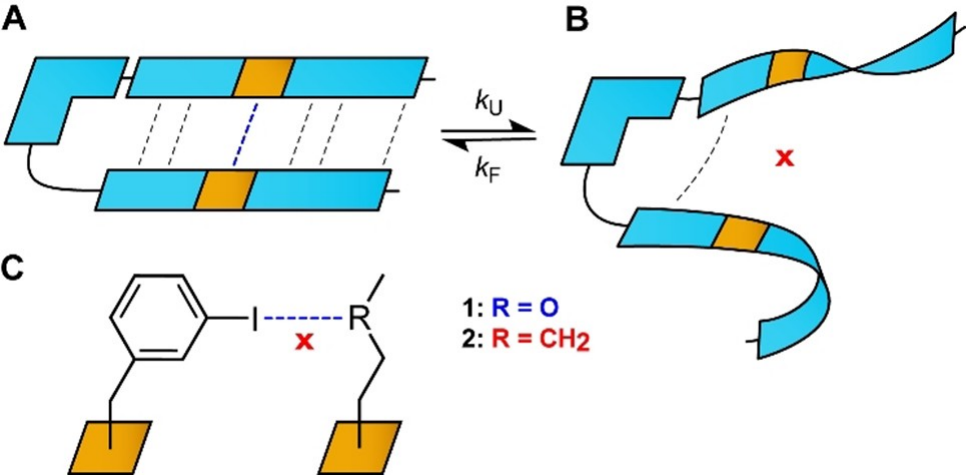
(A, C) The schematic representation of a model system designed for the spectroscopic characterisation of very weak interactions. The interaction of interest, a weak I⋅⋅⋅O halogen bond, is depicted in blue whereas other cooperative forces such as hydrogen bonds and hydrophobic forces are shown as black dashed lines. The system was designed to exist in a two‐state equilibrium between folded (A) and unfolded (B) states with a close to equal molar fraction at room temperature to allow the most accurate characterisation at temperatures close to the inflection point (*T*_m_, melting point) of the folding‐defolding curve (Figure 3). (C) As a reference compound (**2**), we used the closest analogue that is unable to form a halogen bond by substituting the halogen bond acceptor −O− (**1**) with a −CH_2_− (**2**) functionality. The difference in the folding properties of the model system, capable of halogen bonding, and the reference, prevented to form this interaction, provides a handle for the thermodynamic characterisation of the specific weak halogen bond of interest.

**Figure 3:**
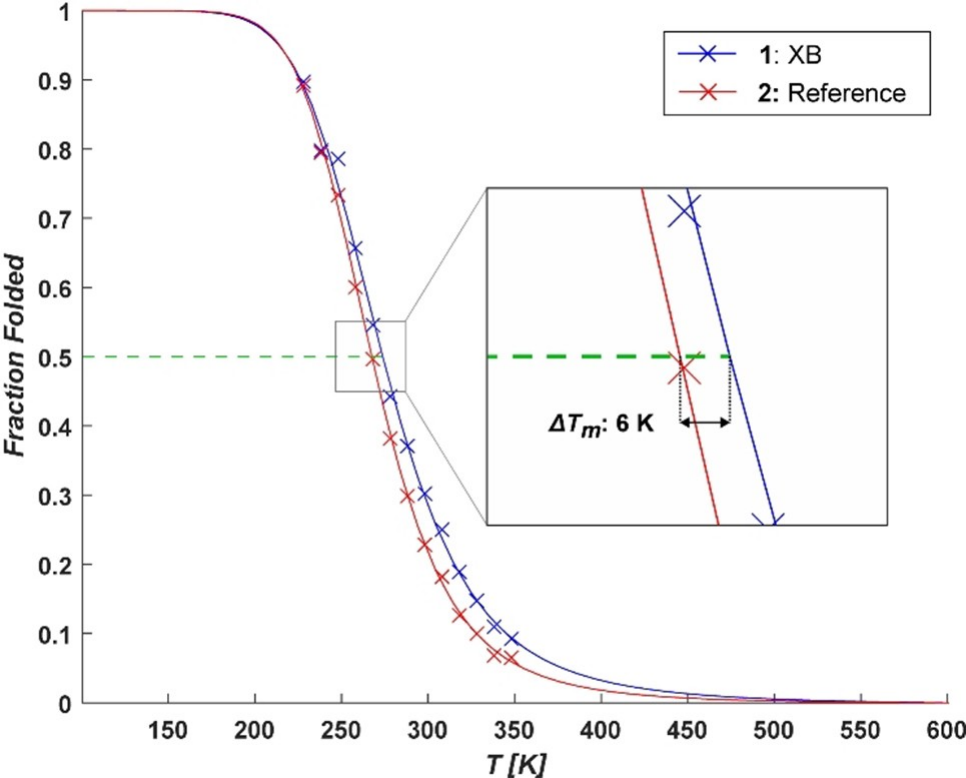
The experimentally determined melting curves of **1** (blue) and **2** (red), given as the molar fraction of folded conformation as a function of temperature. A dashed line (green) at 50 % molar fraction of the folded state indicates the curves inflection points i. e. the melting temperature (*T_m_*). The determined difference in melting temperature (*▵T_m_*) between **1** and **2** is 6 K.

The solution NMR spectroscopic characterisation of halogen bonds^[13b]^ as weak as that of iodobenzene and an ether oxygen is currently not possible as the interaction‐induced chemical shift changes are undetectably small (for details, see pages S12 and S45 in the Supporting Information). Herein, we present a strategy to bridge this scientific gap. We designed a model system (**1**, Figure 1) that exists in a two‐state equilibrium between a folded structure (Figure 2a), which permits formation of an intramolecular halogen bond, and an open, unfolded state (Figure 2b). Using a Monte Carlo conformational search‐based optimisation, we selected a system that is ∼50 % folded at room temperature. Therefore targeting the two‐state equilibrium close to the inflection point of the sigmoid melting curve where thermodynamic characterization is most accurate (Figure 3). This optimal folding ratio has been achieved by the rational modulation of weak forces, i. e. hydrogen bonds, salt bridges and hydrophobic interactions that cooperatively stabilize the folded state. For the quantitative description of one specific interaction, the halogen bond of interest, within this complex system, we designed a reference molecule (**2**). In the latter, the formation of a halogen bond is prevented by the smallest possible change, an −O− to −CH_2_− substitution, to eliminate the halogen bond acceptor site. This reference molecule, **2**, resembles the halogen bonding model system in all respect, but the ability to form a halogen bond. The difference in the folding‐defolding equilibrium of the two compounds, **1** and **2**, originates from this structural difference. All other interactions contributing to the cooperative folding are identical. Hence, the difference in their folding reflects the thermodynamics of the halogen bond of interest. DFT model computations corroborate that incorporation of the I^…^O halogen bond into model system **1** does not significantly alter the interaction energy (for details, see page S46 in the Supporting Information). As the side chains of Thr^2^, Glu^4^, Lys^7^ and Ser^9^ orient to the opposite face of **1** as compared to those of Phe(I)^3^ and Hse(Me)^8^, these do not influence the studied halogen bond.

Compounds **1** and **2** were synthesized on solid‐phase and purified by HPLC; for details see the Supporting Information.

### Thermodynamic analysis

Thermodynamic analysis was performed by observing the temperature dependence of the chemical shifts of ten backbone protons for compounds **1** and **2**, each in 10 K steps over a 130 K temperature range. To allow detection in such a wide temperature range (218 K to 348 K) and in a comparably polar environment, a solvent mixture of CD_2_Cl_2_:DMSO‐*d*_6_ (4 : 1) was used. The chemical shifts of the completely folded and completely unfolded state were estimated, following the procedure described by Munekata,^[19]^ by fitting experimentally observed data to the function describing a two‐state equilibrium (Eq. 1)

(1) $$\delta_{obs}=\delta_{U}+\left\{\frac{\delta_{F}\;-\;\delta_{U}}{1+exp\left[-\;\frac{{\Delta H}_{m}}{R}\;{}^{*}\;\left(\;\frac{1}{T}\;-\;\frac{1}{T_{m}}\;\right)\right]}\right\}$$

where δ_obs_ is the observed chemical shift at temperature *T*, δ_U_ and δ_F_ are the chemical shifts at completely unfolded and folded state. Correspondingly, ▵*H*_m_ denotes the enthalpy change upon unfolding at the melting temperature, *T*_m_, whereas *R* is the molar gas constant. To determine the overall folding rate, we normalized the individual protons’ folding curves to the maximum shift change of each. Subsequently, the melting curves, describing the overall folding of the two systems (Figure 3), were calculated based on the normalized data of all protons (for details, see the Supporting Information) using Equation (1), providing the thermodynamic parameters given in Table 1. The 6 K higher melting point of **1** as compared to **2** indicates that the halogen bond of the former promotes folding. The <1 kJ mol^−1^ absolute difference of the folding Gibbs free energies is in line with that expected for a weak halogen bond. However, the accuracy of the determination of absolute thermodynamic constants is limited, as indicated by the standard errors given in Table 1.

**Table 1:** Thermodynamic parameters for folding of system **1** and **2**.

Compound	*T_m_* [K]	Δ*H_m_* [kJ mol^−1^]	Δ*S_m_* [J K^−1^ mol^−1^]	Δ*G* ^o^ [kJ mol^−1^]^[a]^
**1**	274.0±2.6	23.9±3.3	73.1±12.0	2.1±0.2
**2**	267.6±1.3	26.4±1.7	78.4±6.0	3.0±0.1

[a] Standard Gibbs free energies were calculated for 298 K, assuming Δ*H* to be constant at any temperature.

**Figure 4:**
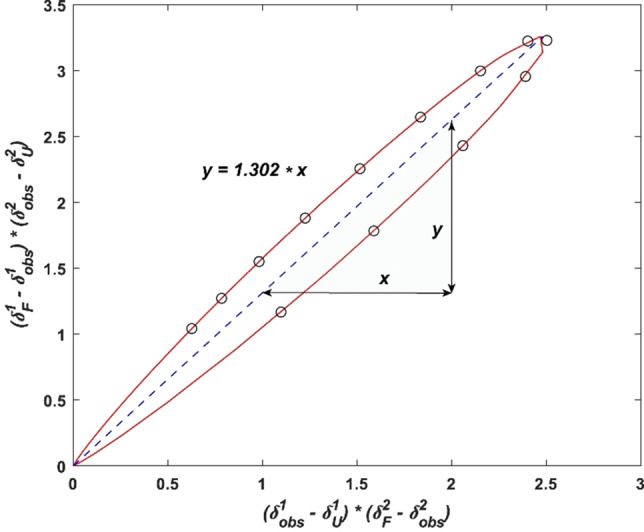
The relative folding ratio of **1** to **2**, $K_{F}^{1/2}$ determined by the slope of plotted melting curves as a fraction of folding rates. The steepness of the slope (blue dashed line) depicts the ratio of $k_{F}^{1}$ to $k_{F}^{2}$ showing a higher stability for **1** relative to **2** indicated by $K_{F}^{1/2}$ >1. The red line represents extrapolated data points applying fitted values for terminal shifts, enthalpy and melting temperature. Black circles outline the experimentally determined shift values within the recorded temperature range.

The change of Gibbs free energy at 298 K, Δ*G*°, was estimated for both peptides using Equation 2

(2) $$\Delta G^{\circ }=-RT{}^{*}\ln k_{F}$$

where

(3) $$k_{F}=\frac{\delta_{U}-\delta_{298}}{\delta_{298}-\;\delta_{F}}$$

providing a ΔΔ*G*°=−0.9 kJ mol^−1^. The relative stability of **1** as compared to **2** can be estimated as the ratio of their folding constants, $K_{F}^{1/2}$ . This can be derived from directly measured chemical shift differences only, without knowledge of the absolute temperature (Eq. (4)), decreasing the uncertainty of the estimation as compared to determination of the absolute thermodynamic constants:

(4) $$K_{F}^{1/2}=\frac{k_{F}^{1}}{k_{F}^{2}}=\frac{\left(\bar{\delta_{U}^{1}}-\;\delta_{obs}^{1}\right)}{\left(\delta_{obs}^{1}-\;\bar{\delta_{F}^{1}}\right)}/\frac{\left(\bar{\delta_{U}^{2}}-\;\delta_{obs}^{2}\right)}{\left(\delta_{obs}^{2}-\;\bar{\delta_{F}^{2}}\right)}$$

where δ_obs_ is the measured chemical shift, $\bar{\delta_{U}}$ and $\bar{\delta_{F}}$ are the shifts in the completely unfolded and completely folded states of compounds **1** and **2**, respectively. $K_{F}^{1/2}$ can be deduced as the slope when plotting $\left(\delta_{obs}^{1}-\bar{\delta_{F}^{1}}\right)\left(\bar{\delta_{U}^{2}}-\delta_{obs}^{2}\right)$ against $\left(\bar{\delta_{U}^{1}}-\delta_{obs}^{1}\right)\left(\delta_{obs}^{2}-\bar{\delta_{F}^{2}}\right)$ (Figure 4). This plot is non‐linear due to the difference in folding enthalpies of the studied compounds.^[20]^ The $K_{F}^{1/2}$ ∼1.3 ratio suggests a ∼30 % higher folding ratio of **1** as compared to **2**. This corresponds to ΔΔ*G*=−0.6 kJ mol^−1^ following:

(5) $$\Delta \Delta G=-R{}^{*}T_{m}^{2}{}^{*}ln\left(K_{F}^{1/2}\right)$$

where $T_{m}^{2}$ (here, 2 denotes compound **2**) is the melting temperature of **2** and $K_{F}^{1/2}$ is the ratio of folding constants, which is in agreement with ΔΔ*G*° estimated using the method of Munekata (Table 1).^[19]^ As this value is estimated from chemical shift differences only, independent of the absolute temperature, it is expected to be more accurate than that obtained from the difference in absolute stabilities (ΔΔ*G* ^o^=−0.9 kJ mol^−1^, Table 1).

### Ensemble analysis

**Figure 5:**
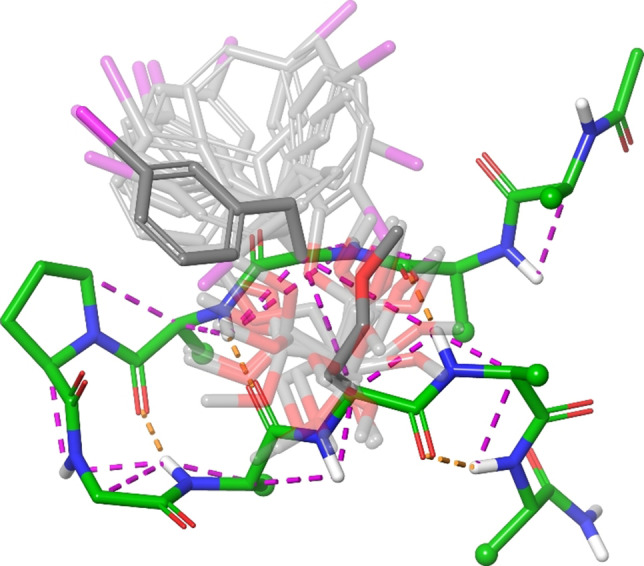
The most abundant folded β‐hairpin conformer of **1**, identified by NAMFIS analysis of solution NMR data. The backbone conformation, shown in green, is reliably determined whereas the orientation of the flexible sidechains, omitted for clarity, is less accurate. Key NOEs used in the ensemble analysis are shown as dashed lines (magenta), respectively. Flexibility of the halogen bonding site is indicated by the rotational freedom of respective sidechains depicted in grey. Hydrogen bonds stabilizing the folded geometry are shown as dashed lines (orange).

In order to identify the halogen bonded conformation of **1**, and to show that the folded population of **1** and **2** truly differ due to their ability versus inability to form an intramolecular halogen bond, we identified solution conformers based on NOEs and *J*‐couplings using the NAMFIS algorithm. This has been used for the ensemble analysis of compounds of comparable size and flexibility, including natural products,^[21]^ peptides[^18^, ^20^, ^22^] macrocycles^[23]^ and drug candidates.[^18^, ^23a^, ^23b^, ^23c^, ^24^] Interproton distances (NOE) and backbone dihedral angles (^3^ *J*_CαH,NH_) were determined from spectra acquired on a 3 mM solution of **1** and **2**, respectively, in CD_2_Cl_2_/DMSO‐*d*_6_ (4 : 1) on a 500 MHz NMR spectrometer equipped with a cryogenic probe. For highest accuracy, NOESY build‐ups with seven mixing times, 100–700 ms, were acquired, and inter‐proton distances derived using the initial rate approximation.^[25]^ ^3^ *J*_CαH,NH_ coupling constants were deduced from ^1^H NMR spectra. The population‐averaged experimental data (interatomic distances and dihedral angles, see the Supporting Information) were deconvoluted into population‐weighted ensembles of solution conformations using NAMFIS.^[22b]^ The input theoretical conformational pool was generated by unrestrained Monte Carlo conformational search with molecular mechanics minimization, within a 42 kJ mol^−1^ energy window from the global minimum. Combination of conformers generated by several Monte Carlo searches applying different force fields ensured sampling of the entire conformational space. As the force fields implemented into the software Schrödinger are not parametrized for halogen bonding, we enriched the theoretical input ensemble of **1** with conformers, in which the I⋅⋅⋅O halogen bond was facilitated by an additional force constant promoting an I⋅⋅⋅O interatomic distance shorter than the sum of the van der Waals radii of I and O. Following this procedure, conformational pools of 236 and 176 conformers for **1** and **2**, respectively, have been used as theoretical inputs for the NAMFIS algorithm, which thereof identified 9 conformers for **1** and 11 for **2**. As the wide conformational variability of the side chains is not possible to cover accurately with the current computational techniques, reliable conclusions can only be drawn regarding the conformation of the backbone. The individual conformations were assessed on the formation of a type II’ β‐turn, on the average interstrand backbone distance and on dihedral angles based on Ramachandran plot analysis to identify folded and unfolded geometries (Figure 2 and Supporting Information). The most abundant folded conformer (24 %) of **1** is shown in Figure 5 whereas all conformers are given in the Supporting Information. As expected, compound **1**, which is capable of forming a cross‐strand halogen bond, showed 17 % higher folded population, 56 %, as compared to **2**, 39 %. Being one of the cooperatively acting forces, the halogen bond may promote folding by promoting conformations, which allow formation of interstrand hydrogen bonds, and may also stabilize the folded conformer itself. The 17 % difference in folding of **1** to **2** is in good agreement with ΔΔ*G* of −0.6 kJ mol^−1^ estimated based on the analyses of variable temperature NMR data described above.

The temperature coefficients of the amide backbone protons, Δδ_NH_/Δ*T*,^[26]^ of **1** and **2** further corroborated the existence of folded conformers in solution that were identified by NAMFIS, indicating the formation of five intramolecular, hydrogen bonds for the amino acids Thr_2_, Glu_4_, Lys_7_, Ser_9_ and the C‐terminal amide (Table 2). The Δδ_NH_/Δ*T* of the amide protons involved in the formation of the intramolecular hydrogen bond network gradually increase towards the termini which is in agreement with a folding initiated by β‐turn formation.^[27]^ The amides of **1** show a slightly yet tendentiously smaller temperature dependence than those of **2**, which is compatible with **1** being more folded than the non‐halogen bonding reference, **2**.

**Table 2:** Amide temperature coefficients (Δδ_NH_/Δ*T*) for **1** and **2**. The amide NH signals are sorted in succession from the β‐turn region towards the termini. The difference in temperature coefficients (ΔΔδ/Δ*T*) of **1** and **2** is in line with a lower folded β‐hairpin population of **2** as compared to **1**.

Δδ_NH_/Δ*T* ^[a]^ [ppb K^−1^]	β‐turn				termini
	Lys‐7	Glu‐4	Ser‐9	Thr‐2	CONH_2_
**1**	2.45	3.85	4.41	5.09	4.56
**2**	2.65	4.25	5.02	5.65	5.21
ΔΔδ/Δ*T*	0.2	0.4	0.6	0.6	0.7

[a] Δδ_NH_/Δ*T*<3 ppb K^−1^ indicates intramolecular hydrogen bonding, 3–5 ppb K^−1^ dynamic equilibrium between hydrogen bonded and solvent exposed states, and >5 ppb K^−1^ indicate solvent exposed protons.

### Residual dipolar coupling analysis: Halogen bond geometry

**Figure 6:**
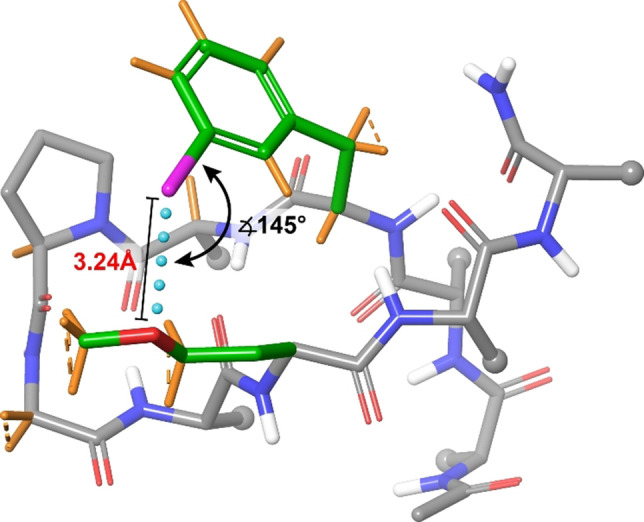
Halogen bonding β‐hairpin conformer as selected by the analysis of residual dipolar couplings, present to 30 % molar fraction. The bond angle and bond distance of the halogen bond are shown. For C−H bonds highlighted in orange experimental residual dipolar coupling constants were obtained.

Whereas NAMFIS analysis provides information on the overall fold, it does not allow conclusions to be drawn on the orientation of the functionalities involved in halogen bond formation. To assess the latter, we collected residual dipolar couplings for **1**, by acquiring Perfect‐CLIP‐^1^H,^13^C‐HSQC^[28]^ under isotropic and anisotropic conditions. Couplings were detected in the indirect F1‐dimension, avoiding multiple bond couplings that may cause peak asymmetry and broadening, impeding the accurate measurement of peak positions upon detection in the F2‐dimension. Alignment was induced using poly‐γ‐benzyl‐L‐glutamate (PBLG),^[29]^ which forms a lyotropic liquid crystal phase with organic solvents above the critical concentration of ∼130 mg mL^−1^ (10 % w/w). We confirmed the alignment by detection of a clean quadrupolar splitting (Δν_Q_∼125 Hz) of the DMSO‐d_6_ deuterium signal. Residual dipolar couplings (^1^ *D_CH_*) were deduced as the difference of the total couplings (^1^ *T*_CH_) measured in the anisotropic and the scalar coupling (^1^ *J*_CH_) obtained in the isotropic solution. They were ‐68 Hz to 58 Hz in magnitude (Table 3). Residual dipolar couplings are of non‐local character, and hence reflect the relative orientation of C−H bonds throughout the molecule, independent of their spatial separation. Their magnitude depends solely on the orientation of the internuclear vector of the corresponding C−H bonds with respect to the external magnetic field. Accordingly, the C−H bond vectors of the halogen bond donor iodo‐Phe^3^ (Figure 6) in combination with those of the methylene groups next to the halogen bond acceptor oxygen of homo‐met‐Ser^8^ provide the spatial arrangement of the donor and acceptor sites, which cannot be deduced from NOEs and *J*‐couplings. In addition to the above side chain protons of the halogen bond donor and acceptor sites, residual dipolar couplings of the backbone's C_α_−H_α_ bonds were included to ensure the accuracy of the alignment tensor deduction. Following literature procedures,^[30]^ we used singular value decomposition to calculate the order parameters and principal frames using the software MSpin.^[31]^ A theoretical input ensemble was generated by resampling the conformational ensemble deduced by NAMFIS. We retained the backbone dihedral angles whilst resampling the orientation of the side chains of interest. To ensure that all potential halogen bonded conformers were present, we enriched the input ensemble by sampling different C−I⋅⋅⋅O angles coherent with halogen bond formation. Singular value decomposition converged with excellent quality descriptors, that is a Cornilescu quality factor *Q*=0.4 % (0.004) and condition number SVD=4.028. Here, the quality factor (Q) is the measure of the goodness‐of‐fit of the theoretical model (structure) to the experimental data, with Q<30 % indicating a good fit.^[32]^ The condition number describes the sensitivity of the equation system to experimental errors, and thus reflects the reliability of the determination of the alignment tensor, with an SVD<30 indicating a robust fit.^[33]^ For details, we refer the reader to the Supporting Information (RDC analysis) and the literature.^[31]^ Overfitting was prevented by an initial model selection procedure, where sub‐ensembles with increasing number of conformations were assessed based on their Χ^2^‐penalty function, as implemented in the Fitter tool of the software MSpin. Residual dipolar coupling analysis indicated 53 % population of folded conformers for **1**, which is in excellent agreement with the outcome of the NAMFIS ensemble analysis (56 %) and with the thermodynamic data obtained from the assessment of the chemical shift melting curves. It further revealed that **1** adopts a folded β‐hairpin encompassing an interstrand I⋅⋅⋅O halogen bond with 30 % population. This corroborates our interpretation of the NAMFIS and variable‐temperature chemical shift analyses, i. e. that the higher folded population of **1** in respect to **2** is due to halogen bonding. Singular value decomposition selected β‐hairpin conformations with a C−I⋅⋅⋅O angle of 145°, from input conformations possessing a wide variety of donor‐acceptor orientations. This observation is in excellent agreement with the bimodal angle preference of ∼160–170° and ∼145–150° for halogen bonds in complex protein‐like environments, as described by Ho et al.^[15a]^ Halogen bonded conformers were seen also for some unfolded conformers, suggesting that formation of an interstrand halogen bond may promote the cooperative folding process.

**Table 3:** The residual dipolar couplings (^1^ *D*_CH_) for **1** were deduced as the difference of the total couplings (^1^ *T*_CH_) measured in an anisotropic solution and the scalar couplings (^1^ *J*_CH_) measured in an isotropic solution.

Residue		^1^ *J*_CH_	^1^ *T*_CH_	^1^ *D*_CH_
3‐Phe(I)‐α	CH	141.4±0.7	128.0±3.5	−13.4±4.2
3‐Phe(I)‐β	CH2	260.2±0.7	127.5±2.0	−67.9±2.6
3‐Phe(I)‐δ^[a]^	CH	160.2±0.6	120.3±1.0	−39.9±1.6
3‐Phe(I)‐δ^[b]^	CH	165.3±0.7	109.7±0.7	−55.6±1.4
3‐Phe(I)‐ϵ	CH	158.6±0.5	107.7±0.4	−50.9±0.9
3‐Phe(I)‐γ	CH	167.1±0.6	188.1±0.7	21.1±1.3
4‐Glu‐α	CH	138.9±0.7	196.4±1.7	57.5±2.4
5‐DPro‐α	CH	145.9±0.5	147.7±1.1	1.8±1.6
6‐Gly‐α	CH2	280.6±0.4	292.6±0.6	6.1±1.0
8‐Hse(Me)‐γ	CH2	283.9±0.3	287.8±0.9	1.5±1.1
8‐Hse(Me)‐ϵ	CH3	416.6±0.1	434.7±0.2	6.0±0.2

[a] CH group *para* to the iodine. [b] CH group *ortho* to the iodine.

## Conclusions

We disclose a strategy for the thorough geometric and thermodynamic characterisation of very weak interactions in solution, demonstrating its scope by the description of an I⋅⋅⋅O halogen bond (∼0.6 kJ mol^−1^). This was achieved by incorporation of the halogen bond donor and acceptor sites into a cooperatively folding system that allowed characterisation of its bond strength and orientation as an intramolecular interaction. In order to demonstrate the impact of the halogen bond on cooperative folding, a reference system lacking the halogen bond acceptor site showed, by NOE and scalar coupling based NAMFIS analysis, to fold 17 % less, as compared to the system designed to form an intramolecular halogen bond. Out of the 56 % folded conformers of the latter, 30 % formed a halogen bond, as shown by the analysis of ^1^H,^13^C residual dipolar couplings obtained by using a dilute solution of alignment medium (PBLG). Importantly, the detection of residual dipolar couplings allowed identification of the C−I⋅⋅⋅O halogen bond angle, 145°, which has previously not been conceivable in solution. It should be noted that this I⋅⋅⋅O halogen bond is much too weak to be detected in solution in an intermolecular setting using standard NMR techniques, whereas the strategy presented here allowed its detailed energetic and geometric characterisation.

Residual dipolar couplings induced by orienting media have previously been used for conformational studies of small molecules; however, so far typically on comparably rigid systems possessing only a few rotatable bonds.^[34]^ The combined use of residual couplings and NOE‐based NAMFIS analysis, as presented here for the first time, is shown to be applicable for describing the conformation of a comparably large and flexible molecule. This approach will extend the range of structural diversity that is assessable, opening for the investigation of more complex, flexible molecular systems.

The presented model system and strategy is expected to allow the investigation of very weak halogen bonds also in polar solutions, such as water. Due to its modularity, the halogen bond donor and acceptor sites can easily be substituted by other halogens and Lewis bases, respectively, allowing systematic studies. In addition, virtually any other type of interaction sites can be included, opening up for the solution investigations of further interactions including chalcogen, pnictogen and tetrel bonds, or even weak hydrophobic forces. The obtained experimental data is expected to help the parametrisation of computational force fields for the accurate description of weak interactions.

Weak interactions, such as halogen bonds, are of pivotal importance for molecular recognition, for instance in medicinal chemistry and in catalysis. As most of these processes rely on a highly dynamic solution‐state, gaining understanding of the geometry and energetics of weak interactions in solution is of high importance.

## Conflict of interest

The authors declare no conflict of interest.

## Supporting information

As a service to our authors and readers, this journal provides supporting information supplied by the authors. Such materials are peer reviewed and may be re‐organized for online delivery, but are not copy‐edited or typeset. Technical support issues arising from supporting information (other than missing files) should be addressed to the authors.

Supporting Information
