# Supplementary material for: Pushing the Limits of Characterising a Weak Halogen Bond in Solution
Source: Chemistry. 2021 Dec 13;28(5):e202103559. doi: 10.1002/chem.202103559 (PMC9300211; doi:10.1002/chem.202103559)
Supplement: Supplementary file 1 — Supporting Information [file CHEM-28-0-s001.pdf]

Chemistry–A European Journal

Supporting Information

Pushing the Limits of Characterising a Weak Halogen Bond in Solution

Stefan Peintner and Máté Erdélyi*

Supporting Information

Synthesis **S2**

1.1 General information *S2*

1.2 General procedure *S2*

1.3 Structure *S3*

1.4 Synthesis *S5*

1.4.1 Synthesis of **1** S5

1.4.2 Synthesis of **2** S5

2 NMR Spectroscopy **S7**

2.1 Chemical shift assignments *S7*

2.2 NOE-buildup derived interproton distances *S8*

2.3 Titration of diethylether to iodobenzene *S12*

2.4 Variable temperature experiments *S13*

2.4.1 Amide temperature coefficient S13

2.4.2 Melting curve analysis based on H_α shifts S16

3 Computational conformation sampling **S20**

3.1 Conformational search for NAMFIS analysis *S20*

3.2 Conformational search for RDC analysis *S21*

4 NAMFIS analysis **S23**

5 RDC analysis **S35**

6 Thermodynamic analysis **S40**

7 DFT Calculations **S45**

8 NMR spectra **S47**

9 HRMS Spectrograms **S62**

10 References **S63**

The original NMR FIDs are freely available on Zenodo as DOI: 10.5281/zenodo.5504456.

Synthesis

1.1 General information

Compounds **1** and **2** were synthesized by automated Fmoc-protected solid-phase peptide synthesis (SPPS) on a Prelude® Peptide synthesizer from Protein Technologies. Reagents and solvents commercially available were used without further purification. Reactions were monitored by LC-MS and MALDI. LC-MS analysis was performed on an Agilent 1100 series HPLC with a C18 Atlantis T3 column (5µm, 50 x 3 mm). A MeCN/H_2O gradient (1-99% MeCN proportion) with a flow rate of 0.75 L/min over 6 min was applied and a Waters micromass ZQ (model code: MM1) mass spectrometer in electrospray ionization (ESI) mode was used for detection. The MALDI system is a Bruker autoflex® II TOF/TOF Tandem MS. Compounds **1** and **2** were purified on preparative RP-HPLC using a Varian ProStar system equipped with a single wavelength detector at λ = 215 nm, and a RP Hichrom Kromasil C8 column (10 µm, 250 x 21.2 mm). A Gradient of MeCN/H_2O (+ 0.1% TFA) (20-60% MeCN proportion) as mobile phase were used with a flow rate of 8 mL/min. High resolution LC-MS analyses were performed on a Sciex QStar® XL with a Halo RP Amide C18 (2.6 µm, 50 x 2.1 mm) column. Gradient used was from 5-99% MeCN/H_2O at pH 4. TOF-MS was run with 1 scan/s and external mass reference. A detailed synthetic scheme is outlined in section 1.2. All amino acids were used as their L stereoisomer, if not stated otherwise.

1.2 General procedure

Compounds **1** and **2** were synthesized using the N_α-Fmoc protection scheme on a 150 µmol scale. *Tert*-butyl (tBu) protection was chosen for Thr, Ser and Glu, *tert*-butyloxycarbonyl (Boc) for Lys. We used the NovaPEG Rink amide resin (loading 0,53 mmol/g) as solid-phase. The resin was allowed to swell in DMF for 1 h prior to use. The first amino acid was manually loaded on the resin, and success of the loading was checked with Kaiser-test. Fmoc-AA-OH (2eq) was dissolved in 2mL DMF, HCTU (1.8 eq) and DIPEA (4 eq) was added. The mixture was added to the pre-swelled resin, shaken for 1 h, washed with DMF twice. Piperidine (20%) in NMP was used as Fmoc-deprotection reagent. Further consecutive coupling of Fmoc-protected amino acids was performed by automated SPPS adding amino acid solution (125 mM in NMP, 2.5 mL, 2.1 eq) to the resin. HCTU in DMF (0.65 mL of 0.45 M solution in DMF, 1.9 eq), DIPEA (0.3 mL of 2M in NMP, 4eq) and additional NMP (4.5 mL) were added. Coupling was allowed to proceed for 20 min, washed and repeated once. Special amino acids (Fmoc-*meta*-iodo-Phe-OH for **1** and **2** at position 3, Fmoc-O-methyl-homo-Ser-OH for **1** at position 8) were attached manually by single coupling with Fmoc-AA-OH (1.8 eq, 0.27 mmol), HCTU (1.44 eq, 0.216 mmol) and DIPEA (4 eq, 0.6 mmol) in 3 mL DMF for 1 h. After incorporation of the last amino acid, Fmoc-Leu-OH, the N-terminal Fmoc protecting group was removed and the resulting free amine was acetyl capped. Ac_2O (30 eq) was dissolved in 4 mL DMF, and was added to the pre-swelled resin. Subsequently, a solution of DIPEA (30 eq) in 3 mL DMF was added to the mixture and was shaken for 30 min, washed and repeated once. To prepare the resin for peptide-cleavage DMF-washed resin was further washed with DCM and MeOH thoroughly and dried under vacuum overnight. The peptide was then released from the resin, Boc and tBu protecting groups by treatment with a mixture of TFA, H_2O and triisopropylsilane (95:2.5:2.5; 10 mL). The reaction mixture was gently agitated for 3 h, filtered and concentrated by a stream on N_2. Ice-cold ether was used to precipitate the free peptide and to wash

away scavenger products. The ether dispersion was centrifuged and the ether was carefully decanted. The peptide was dispersed in cold ether and centrifuged again. After removal of the supernatant ether resulting peptide was dissolved in H_2O:MeCN (7:3) and lyophilized.

The crude peptide was purified by reversed phase HPLC according to conditions outlined in the general information.

1.3 Structure

The peptides synthesized and analyzed in this work are shown in Figure S1. Synthetic procedures can be found in section 1.2. Full characterization by NMR spectroscopy is shown in section 3. Conformational analysis is discussed in the main text, and additional data can be found in section 4. Abbreviations for amino acids are as follows: G: Gly, Glycine, I: Ile, Isoleucine, L: Leu, Leucine, ^{D}P: ^{D}Pro, D-Proline, F(I): m-I-Phe, Phe(I), meta-Iodo-Phenylalanine, E: Glu, Glutamic acid, K: Lys, Lysine, S: Ser, Serine, T: Thr, Threonine, Hse(Me): hmSer, *O*-methyl-Homoserine, Nle: Norleucine.

1

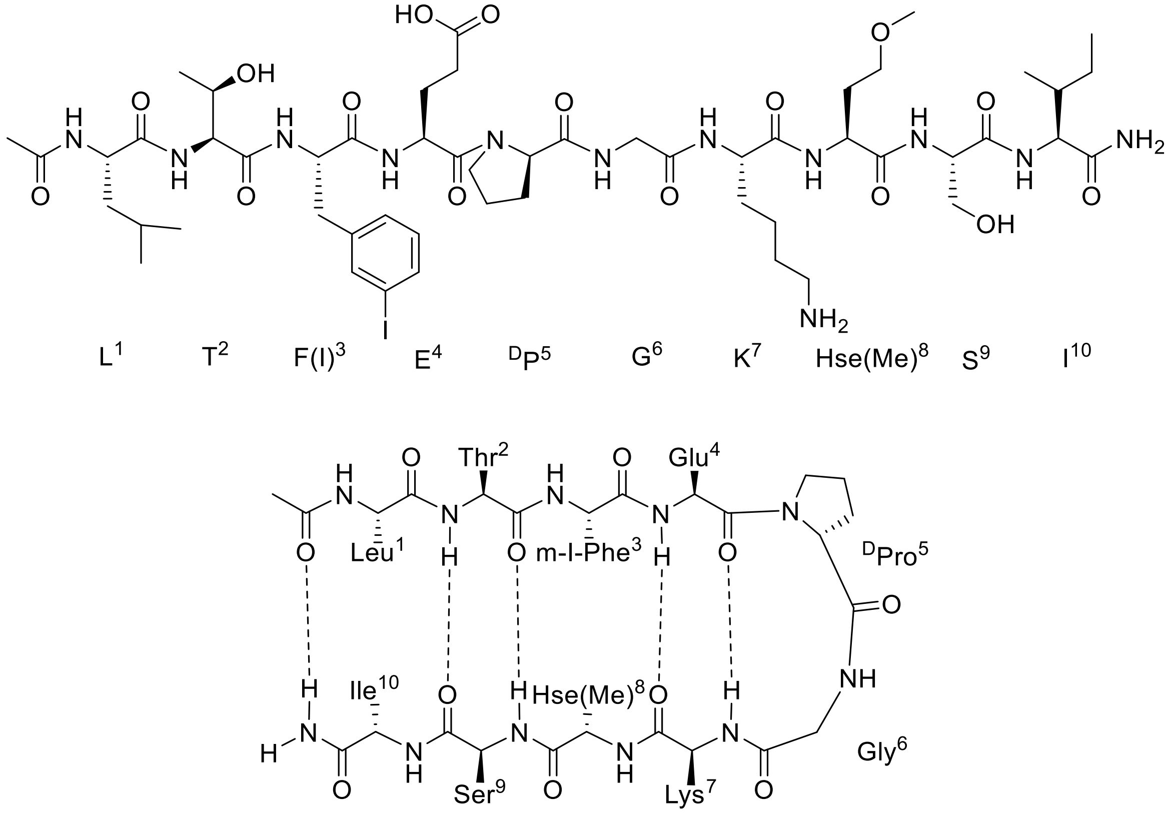

Figure S1: The structure of **1**: above the linear sequence as overview, below the intended antiparallel β-hairpin secondary structure. The halogen bonding site is positioned at m-I-Phe^{3} and Hse(Me)^{8}.

2

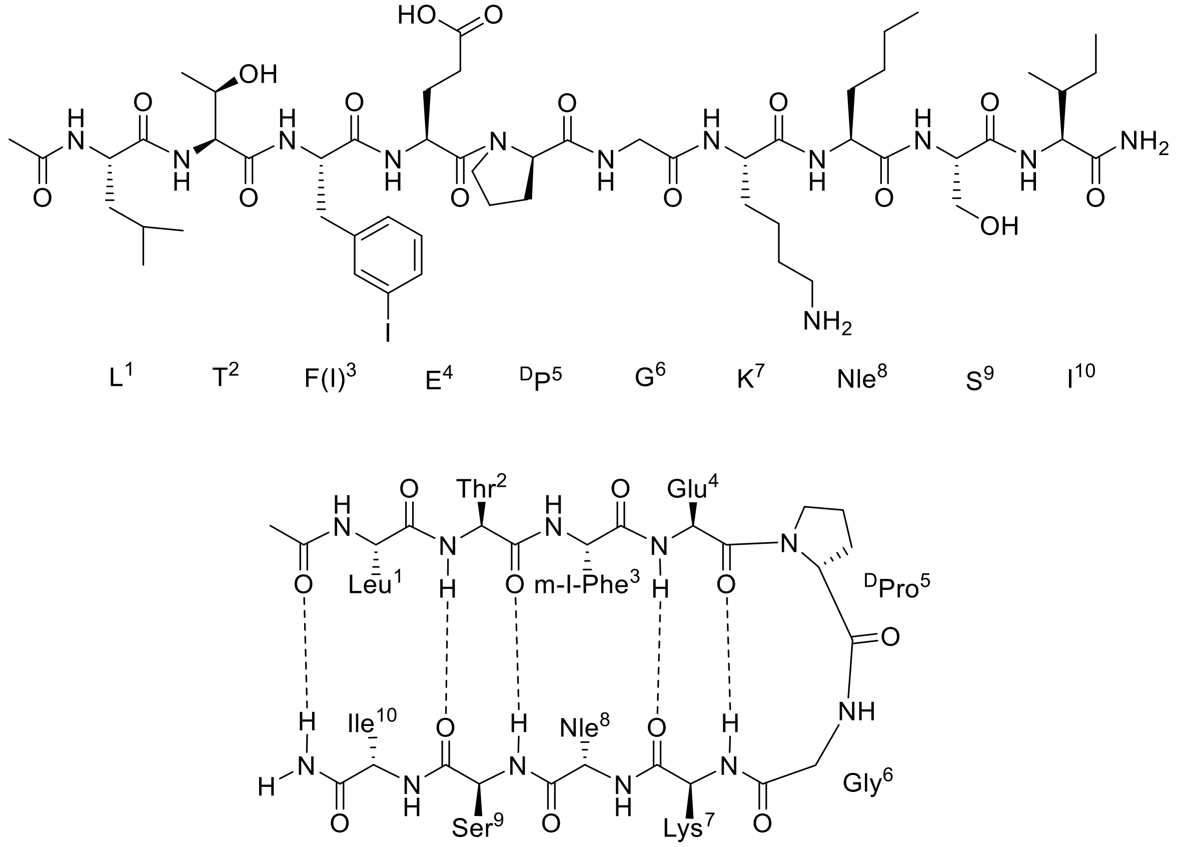

Figure S2: The structure of **2**: above the linear sequence as overview, below the intended antiparallel β-hairpin secondary structure. This peptide served as a reference molecule, lacking the Lewis-base (halogen bond acceptor) amino acid in position 8 opposing m-I-Phe[3].

1.4 Synthesis

1.4.1 Synthesis of **1**

The linear peptide **1** was synthesized as described above using NovaPEG Rink-amide resin (loading: 0.53 mmol/g) on a 150 µmol scale. Loading the pre-swelled resin (DMF) with the first amino acid (Fmoc-L-Ile-OH^{10}, 2eq) was performed using HCTU (1.8 eq) and DIPEA (4 eq) in DMF. A Kaiser test was performed to proof sufficient coupling. Piperidine (20% in DMF) was used for Fmoc deprotection 2×15 min. Fmoc-L-Thr(tBu)-OH^{9} and Fmoc-L-homo-methyl-Serine-OH^{8} were coupled alike. Kaiser test after coupling of Thr resulted in red resin beads and a clear solution. This is reported typical for serine or threonine amino acids. Subsequent steps (Lys(Boc)^{7}-Gly^{6}-^{D}Pro^{5}-Glu(OtBu)^{4}) were coupled using an automated synthesizer. Conditions are given in the general procedure of peptide synthesis. Fmoc-protected peptide was acquired from the synthesizer and manually Fmoc-deprotected by treatment with 20% piperidine in DMF. Coupling of Fmoc-meta-Iodo-Phe-OH^{3} (1.8 eq) was performed using HCTU (1.44 eq) and DIPEA (4 eq) in DMF. Final amino acid sequence (Thr^{2}-Leu^{1}) was coupled on the synthesizer. After deprotection of the final amino acid Fmoc-Leu^{1} with 20% piperidine in DMF the obtained free terminal amino group was acetylated by treatment with Ac_2O (30 eq) and DIPEA (30 eq) in DMF twice. Success of the capping step was confirmed by Kaiser test analysis. The resin was prepared for TFA cleavage by washing 3x with DCM, 3x with MeOH and dried in vacuum over-night. Cleavage and removal of protecting groups was performed according to protocol stated above. Final product **1** was purified by prep-HPLC with an overall yield of 22%. HRMS m/z $[M+H]^+$ calculated for $C_{53}H_{85}IN_{12}O_{16}$: 1273.5329, found: 1273.5370.

1.4.2 Synthesis of **2**

The linear peptide was synthesized as the previous one but the 7 amino acid sequence Ile^{10}-Ser(tBu)^{9}-Nle^{8}-Lys(Boc)^{7}-Gly^{6}-D-Pro^{5}-Glu(OtBu)^{4} was coupled by automated synthesis following the above given protocol. The Fmoc-protected peptide segment acquired from the synthesizer was Fmoc-deprotected with 20% Piperidine in DMF, followed by manual coupling of Fmoc-meta-Iodo-Phe-OH^{3} (1.8 eq) using HCTU (1.44 eq) and DIPEA (4 eq) in DMF. Final amino acids Thr(tBu)^{2}-Leu^{1} were coupled in automated fashion. Preparation, cleavage and purification was performed as stated above giving **2** in 23% yield. HRMS m/z $[M+H]^+$ calculated for $C_{54}H_{87}IN_{12}O_{15}$: 1271.5537, found: 1271.5374.

Scheme S1: Synthesis of peptides **1** and **2**

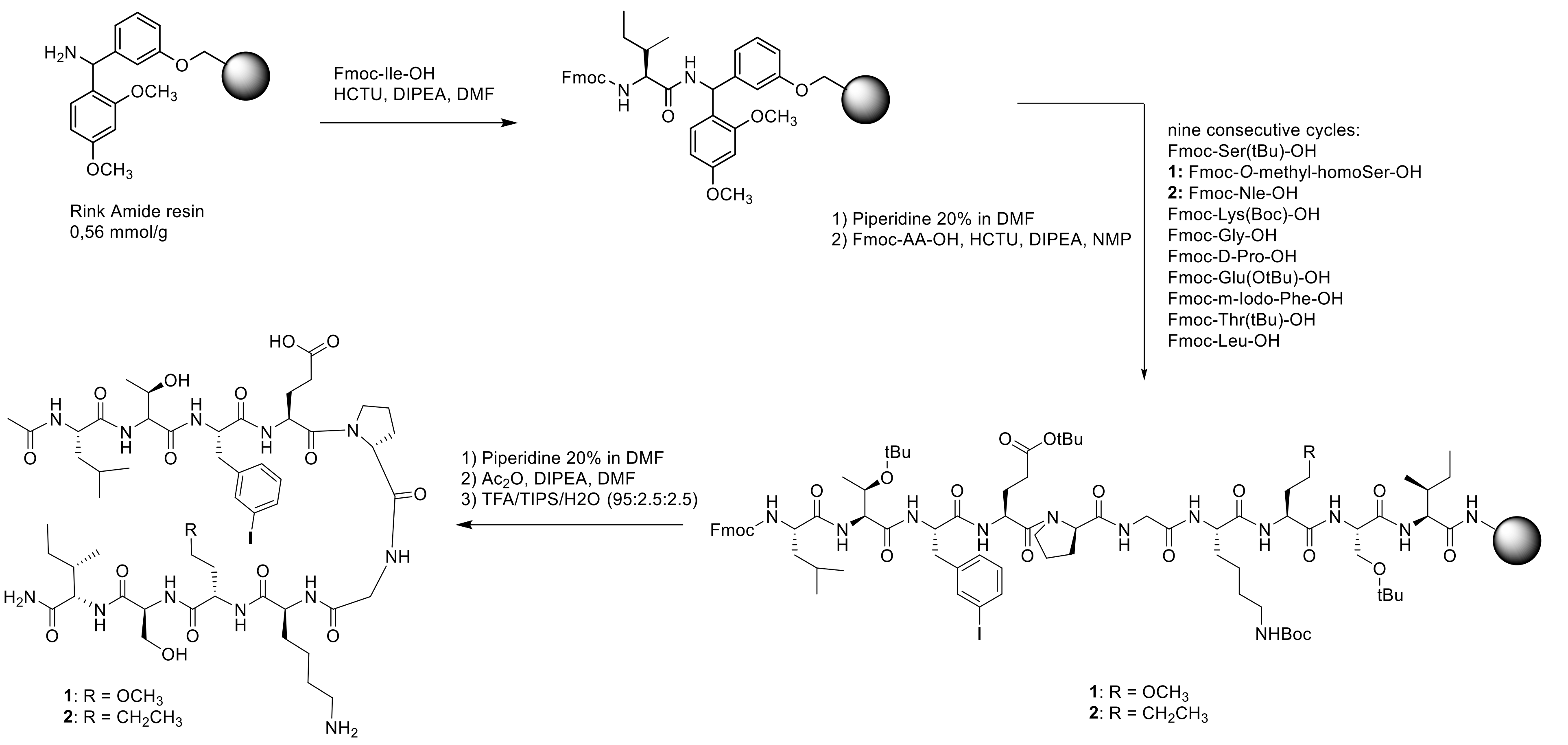

2 NMR spectroscopy

All spectra, including ^{1}H and ^{13}C NMR and 2D COSY, TOCSY, HSQC, HMBC and NOESY experiments were recorded at 25°C/298K on a 600 MHz BRUKER Avance Neo spectrometer equipped with a TCI cryogenic probe (CRPHe TR-^{1}H &^{19}F/^{13}C/^{15}N 5mm-EZ). Samples were prepared by dissolving the lyophilized peptide in 120 µl DMSO-d_6 and further diluting it with 480 µl CD_2Cl_2 yielding ~2.4 mM concentration. ^{1}H NMR assignment was derived by TOCSY-NOESY backbone walk and subsequent assignment of individual sidechain signals. Chemical shifts are shown in tables S1 (**1**) and S2 (**2**), respectively. $^{3}J_{C\alpha H\text{-}NH}$ coupling constants were determined from ^{1}H NMR and are given in Table S3. Variable temperature experiments were recorded on a Varian Agilent MR400-DD2 equipped with an OneNMR probe. The temperature range was in the limit of signal detection and boiling point of the NMR solvent mixture, from -45°C/228K to +75°C/348K. To determine residual dipolar couplings a solution of peptide (≈ 6mg in 20% DMSO-d_6 in CD_2Cl_2) was added to 80 mg PBLG (poly-benzyl-L-glutamate, MW: 5.54 E^5 g·mol^{-1}) in an NMR tube. Gel formation was observed and the tube was intensely agitated using an NMR tube centrifuge. As internal standard a flame sealed glass capillary containing dry DMSO-d_6 was added inside the NMR tube. ^{2}H-NMR was recorded to confirm alignment by a clean split (≈125Hz) of the oriented solvent signal. F1 coupled ^{13}C-^{1}H CLIP-HSQCs were recorded for two samples, an isotropic with the peptide free in solution and anisotropic with the peptide aligned in the PBLG gel. RDCs were obtained as the difference of observed C-H coupling constants. For details see section 5.

2.1 Chemical shift assignments

Table S1: ^{1}H NMR chemical shift assignment [δ, ppm] of **1** in 20% DMSO-d_6 in CD_2Cl_2.

Residue	**Hα**	**Hα1**	**Hα2**	**Hβ**	**Hβ1**	**Hβ2**	**Hγ**	**Hγ1**	**Hγ2**	**Hδ**	**Hδ1**	**Hδ2**	**Hε**	**Hζ**	**NH**
Ac	1.93														
Leu[1]	4.38				1.52		1.66				0.87	0.90			7.89
Thr[2]	4.28			4.02			1.00								7.78
Phe(I)[3]	4.82				2.85	3.13					7.61*	7.21*	6.99	7.51	7.93
Glu[4]	4.55				1.96	2.16		2.29							8.31
D-Pro[5]	4.32				2.07	1.91		1.86	2.03		3.71	3.61			
Gly[6]		3.76	3.83												8.09
Lys[7]	4.36				1.78	1.87		1.45			1.66		2.88		7.71
hmSer[8]	4.47				1.99	1.83		3.35					3.21		8.05
Ser[9]	4.43				3.59	3.73									7.87
Ile[10]	4.27			1.84			0.90	1.48	1.14	0.85					7.54
$CONH_2$															6.40, 7.23

* Values correspond to chemically different CH aromatic protons. The signal at 7.61 ppm represents the δ-proton *ortho* to the iodine and the signal at 7.21 ppm represents the δ-proton *para* to the iodine.

Table S2: ^{1}H NMR chemical shift assignment [δ, ppm] of **2** in 20% DMSO-d6 in CD_2Cl_2.

Residue	**Hα**	**Hα1**	**Hα2**	**Hβ**	**Hβ1**	**Hβ2**	**Hγ**	**Hγ1**	**Hγ2**	**Hδ**	**Hδ1**	**Hδ2**	**Hε**	**Hζ**	**NH**
Ac	1.94														
Leu[1]	4.48				1.53		1.64				0.86	0.90			7.76
Thr[2]	4.36			4.02			1.00								7.91
Phe(I)[3]	4.92				2.84	3.11					7.60*	7.19*	6.98	7.51	8.01
Glu[4]	4.64				2.04	1.86		2.29							8.36
D-Pro[5]	4.32				1.98	2.18		1.93	2.09		3.64	3.72			
Gly[6]		3.75	3.86												8.17
Lys[7]	4.48				1.88	1.78		1.47			1.67		2.88	n.a.	7.69
Nle[8]	4.39				1.59	1.69		1.26			1.25		0.83		8.05
Ser[9]	4.50				3.69	3.55									7.98
Ile[10]	4.34			1.83			0.92	1.51	1.14	0.87					7.61
$CONH_2$															6.30, 7.35

*: Values correspond to chemically different CH aromatic protons. The signal at 7.60 ppm represents the δ-proton *ortho* to the iodine and the signal at 7.19 ppm represents the δ-proton *para* to the iodine.

Table S3: $^3J_{CHα\text{-}NH}$ couplings for **1** and **2**.

	**$^3J_{CHα\text{-}NH}$**	
Residue	**1**	**2**
Leu[1]	7.4	7.6
Thr[2]	7.5	7.6
Phe(I)[3]	8.2	8.5
Glu[4]	7.4	7.7
D-Pro[5]	-	-
Gly[6]	6.2*	6.1*
Lys[7]	7.4	7.9
hmSer[8]/Nle[8]	7.4	7.4
Ser[9]	7.3	7.7
Ile[10]	8.4	n.a.

*Coupling constant deduced from the NH_{Gly} signal that appears as a triplet.

2.2 NOE-buildup derived interproton distances

To obtain quantitative inter-proton distances seven ^{1}H,^{1}H-NOESY experiments were evaluated with increasing mixing times ranging from 100 ms to 700 ms. Experiments were recorded in random order to counteract systematic errors. Spectra were acquired with 512 × 4096 complex points (F1 × F2) and a spectral width of 7800 Hz. Recycle relaxation delay (d1) was set to 2.5 sec. Deuterium signal of

DMSO-d6 was used for the lock signal. Diagonal and cross peaks were integrated for all mixing times and individual NOE intensities taken as the absolute integral were normalized according to:

$$\eta_{norm,ij} = \sqrt[2]{\frac{|cross\,peak_{ij} * \; cross\,peak_{ij}|}{|diagonal\,peak_i * \; diagonal\,peak_j|}} \tag{S1}$$

per mixing time, respectively. A minimum of four normalized intensities, η_{norm}, for consecutive mixing times describing a linear initial build-up with $R^2 \geq 0.95$ were used to determine the build-up rate σ_{ij}. Build-up rates of locked-in-distance protons were used as distance reference (e.g.: geminal protons: 1.78Å, ortho protons: 2.50Å).[1] Distances were calculated according to

$$r_{ij} = r_{ref}\left(\frac{\sigma_{ref}}{\sigma_{ij}}\right)^{\frac{1}{6}} \tag{S2}$$

where r_{ij} is the resulting distance between proton i and j, r_{ref} is the fixed distance of the reference proton pair, σ_{ref} is the build-up rate of the used reference protons and σ_{ij} is the build-up rate of the protons of interest using normalized NOE intensities. Obtained distances are given in the Tables S4-S5.

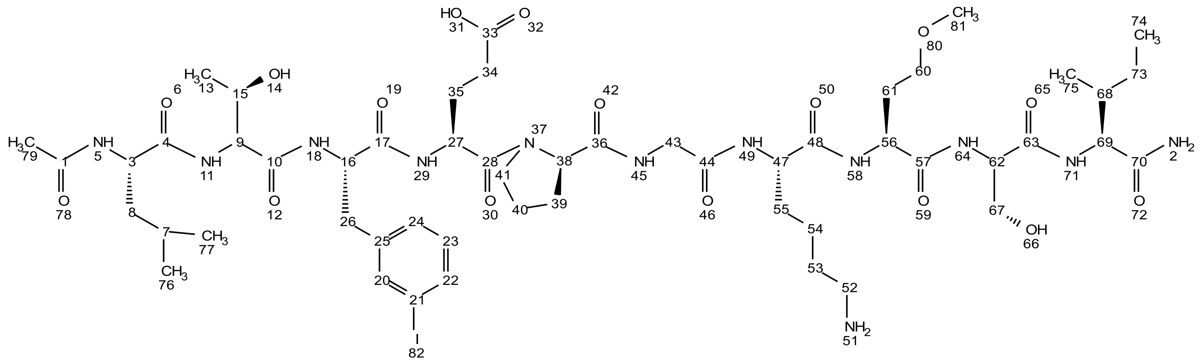

Figure 3: Structure and proton numbering as used for the assignment of **1**.

Table S4: Interproton distances derived from NOE build-up analysis of **1**.

	Type	Proton i	Proton j	δ_i (ppm)	δ_j (ppm)	σ_{ij}	R^2	Distance r_{ij} (Å)
1	CαH-CαH	16	56	4.82	4.47	1.09E-05	0.96	2.77
2	NH-CαH	29	27	8.31	4.55	1.73E-05	0.98	2.57
3	NH-CαH	29	16	8.31	4.82	4.82E-05	0.99	2.16
4	NH-CαH	45	38	8.09	4.32	3.29E-05	0.99	2.31
5	NH-CαH	58	47	8.05	4.36	5.7E-05	0.99	2.10
6	NH-CαH	58	56	8.05	4.47	1.5E-05	0.96	2.63
7	NH-CαH	18	9	7.93	4.28	3.27E-05	0.99	2.31
8	NH-CαH	18	16	7.93	4.82	1.84E-05	0.99	2.54
9	NH-CαH	64	56	7.87	4.47	4.01E-05	0.97	2.23
10	NH-CαH	64	62	7.87	4.43	1.77E-05	0.98	2.56
11	NH-CαH	49	47	7.71	4.36	1.19E-05	0.99	2.73
12	NH-CαH	71	62	7.54	4.43	1.57E-05	0.99	2.61

13	NH-Gly	49	43b	7.71	3.76	1.23E-05	0.99	2.72
14	NH-Gly	49	43a	7.71	3.83	9.38E-06	0.99	2.84
15	NH-Gly	45	43b	8.09	3.76	3.69E-05	0.98	2.26
16	NH-CαH	64	16	7.87	4.82	2.07E-06	0.96	3.66
17	NH-CαH	11	3	7.78	4.38	3.36E-05	0.96	2.30
18	NH-NH	45	49	8.09	7.71	1.81E-05	0.99	2.55
19	NH-NH	29	18	8.31	7.93	6.7E-06	0.96	3.01
20	CαH-Pro	27	41a	4.55	3.71	3.07E-05	0.96	2.33
21	CαH-Pro	27	41b	4.55	3.61	3.71E-05	0.99	2.26
22	CαH-CβH	9	15	4.28	4.02	1.47E-05	0.96	2.64
23	NH-CβH	64	67b	7.87	3.59	2.46E-06	0.95	3.55
24	NH-CβH	49	55b	7.71	1.78	1.7E-05	0.96	2.57
25	CαH-CγH	3	7	4.38	1.66	5.54E-06	0.96	3.10
26	CαH-CγHs	27	34ab	4.55	2.29	3.61E-06	0.99	3.33
27	NH-CγHs	49	54ab	7.71	1.45	5.28E-06	0.96	3.13
Ref.	*ortho* CHs	24	23	7.21	6.99	2.02E-05	0.83	2.50

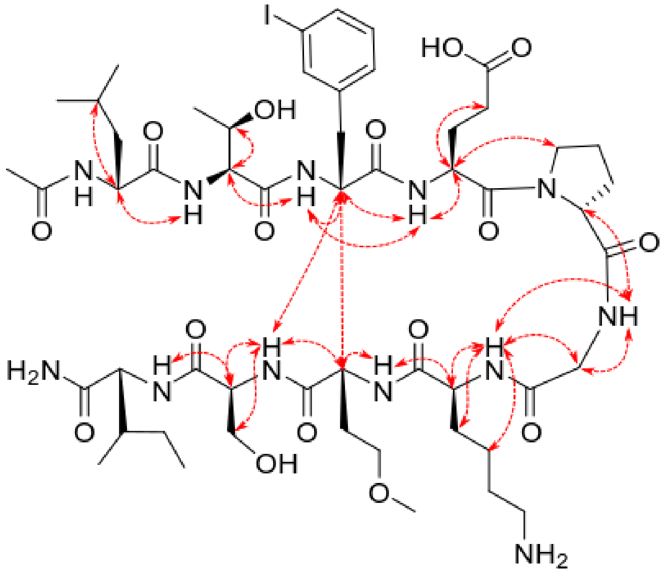

Figure S4: NOE interproton distances used for the NAMFIS analysis of **1**.

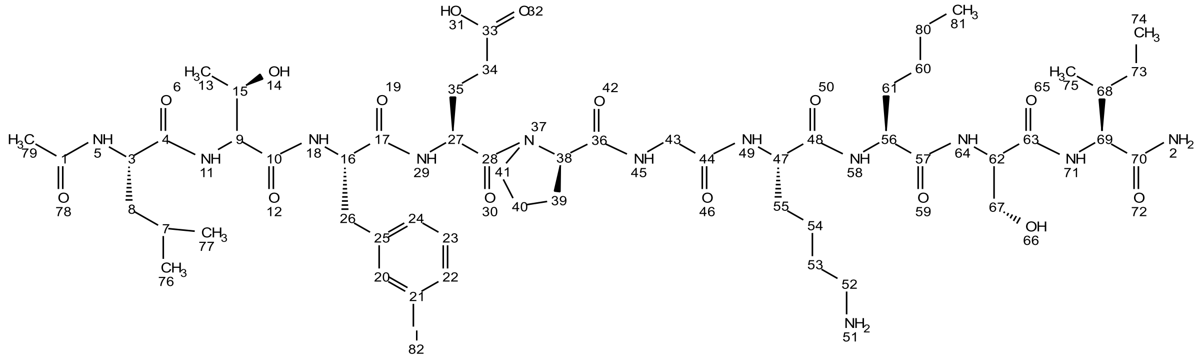

Figure S5: Structure and proton numbering as used for assignment of **2**.

Table S5: Interproton distances derived from NOE build-up analysis for **2**.

	Type	Proton i	Proton j	δ_i (ppm)	δ_j (ppm)	σ_{ij}	R^2	Distance r_{ij} (Å)
1	CaH-CaH	16	56	4.92	4.38	2.02E-05	0.96	2.62
2	NH-CaH	49	43a	7.69	3.86	1.29E-05	0.99	2.83
3	NH-CaH	29	16	8.36	4.92	5.2E-05	0.99	2.24
4	NH-CaH	18	9	8.01	4.36	3.61E-05	0.99	2.38
5	NH-CaH	18	16	8.01	4.92	2.02E-05	0.99	2.62
6	NH-CaH	58	47	8.05	4.48	5.67E-05	0.98	2.21
7	NH-CaH	49	43b	7.69	3.74	2.5E-05	0.97	2.53
8	NH-CaH	29	27	8.36	4.64	1.88E-05	0.96	2.65
9	NH-CaH	58	56	8.05	4.38	1.79E-05	0.99	2.68
10	NH-CaH	29	56	8.36	4.38	7.45E-06	0.99	3.10
11	NH-CaH	45	27	8.16	4.64	2.41E-06	0.99	3.74
12	NH-CaH	5	69	7.75	4.36	6.35E-07	0.99	4.67
13	NH-CaH	64	62	7.98	4.50	2.48E-05	0.98	2.54
14	NH-CaH	49	38	7.69	4.33	2.9E-06	0.98	3.62
15	NH-CaH	11	3	7.90	4.47	5.49E-05	0.97	2.22
16	NH-CaH	49	47	7.69	4.48	1.23E-05	0.97	2.85
17	NH-NH	29	18	8.36	8.01	1.7E-05	0.99	2.70
18	NH-NH	45	49	8.16	7.69	2.4E-05	0.99	2.55
19	NH-NH	11	5	7.90	7.75	1.2E-05	0.98	2.86
20	NH-NH	58	49	8.05	7.69	5.93E-06	0.97	3.22
21	NH-NH	29	49	8.36	7.69	4.77E-06	0.99	3.34
22	CaH-Pro	27	41b	4.64	3.65	5.36E-05	0.99	2.23
23	CaH-Pro	27	41a	4.64	3.72	4.22E-05	0.97	2.32
24	CaH-Pro	27	38	4.64	4.33	2.93E-06	0.95	3.62
25	NH-Pro	45	38	8.16	4.33	4.28E-05	0.98	2.31
26	NH-Pro	45	39a	8.16	2.17	5.8E-06	0.96	3.23
27	CaH-CbH	9	15	4.36	4.02	1.71E-05	0.95	2.70
28	NH-CbH	29	55a	8.36	1.88	3.66E-06	0.95	3.49
29	NH-CbH	29	26a	8.36	3.12	6.12E-06	0.95	3.20
30	NH-CbH	18	15	8.01	4.02	1.26E-05	0.95	2.84
31	NH-CbH	49	55b	7.69	1.79	1.03E-05	0.95	2.93
32	NH-CbH	18	26a	8.01	3.12	8.04E-06	0.98	3.06
33	NH-CbH	11	8ab	7.90	1.53	5.52E-06	0.98	3.26
34	NH-CbH	49	55a	7.69	1.88	9.99E-06	0.96	2.95
35	NH-CbH	11	15	7.90	4.02	8.46E-06	0.95	3.03
36	NH-CgH	18	13	8.01	0.99	2.19E-06	0.96	3.80
37	NH-arom	18	20	8.01	7.61	9.08E-06	0.98	3.00
Ref.	*geminal* CH_2	41a	41b	3.72	3.65	0.000207	0.95	1.78

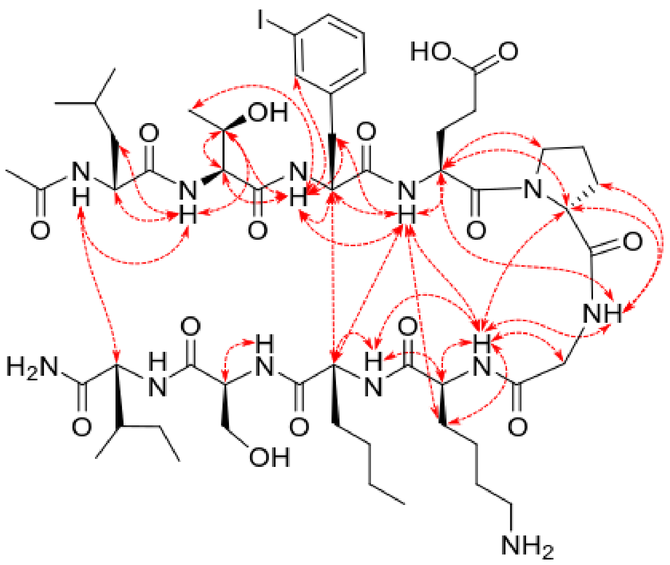

Figure S6: NOE interproton distances used for the NAMFIS analysis of **2**.

2.3 Titration of diethylether to iodobenzene

To test if a very weak halogen bond can be detected intermolecularly we titrated equivalents of dietheylether into a solution of iodobenzene (20mM) in $CDCl_3$. Shifts were followed by ^1H and ^{13}C NMR and are depicted in Figure 7 and Figure 8. No significant carbon chemical shift changes for iodobenzene were visible, whereas minor proton shifts were detected in the ^1H NMR.

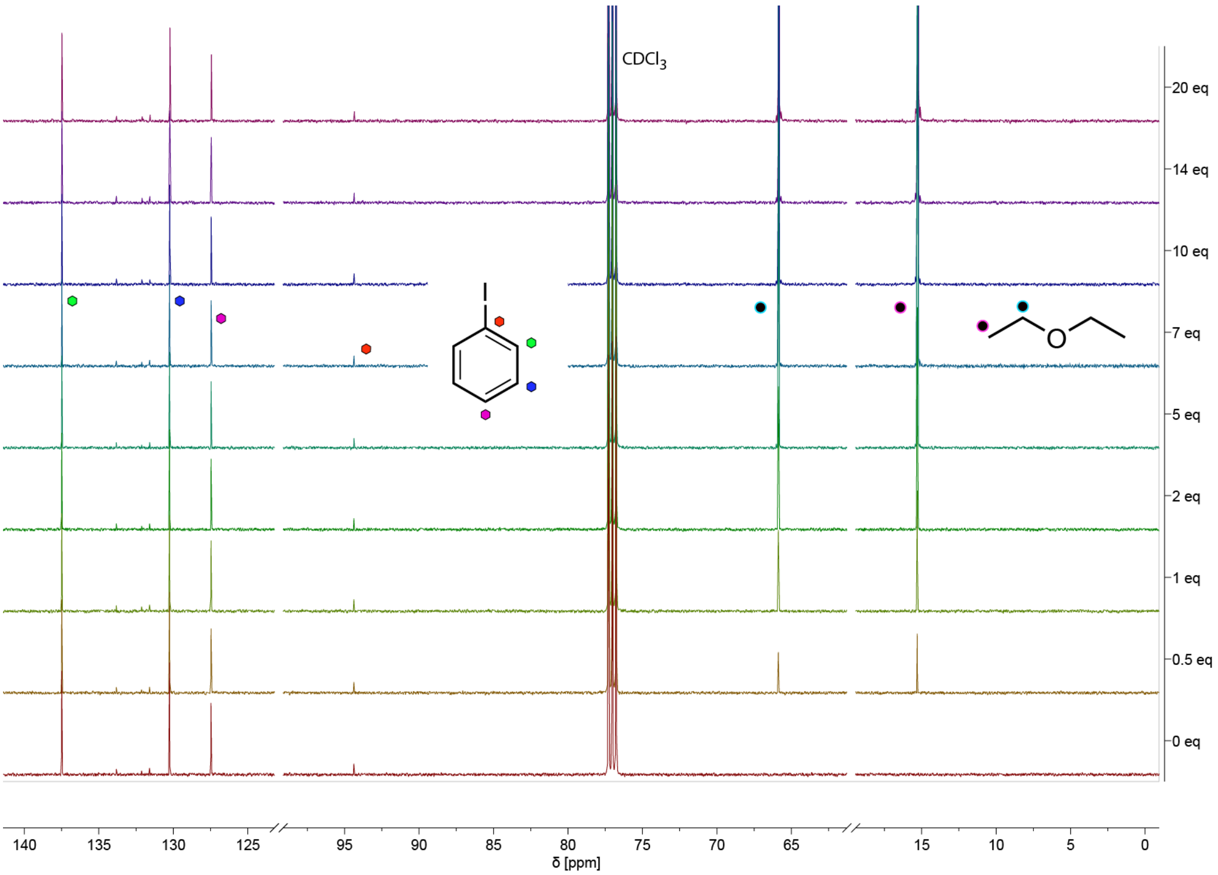

Figure 7: ^{13}C NMR of titration experiment adding equivalents of diethylether to a solution of iodobenzene in $CDCl_3$.

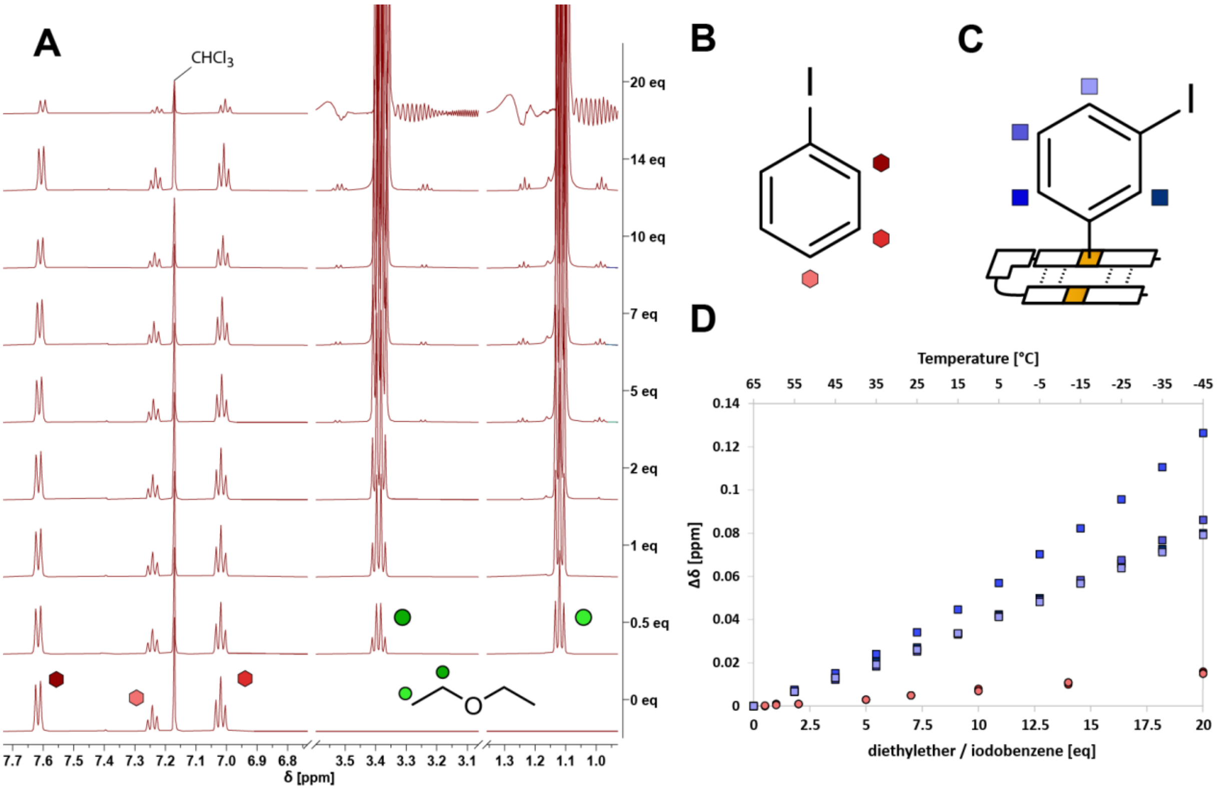

Figure 8: (**A**) ^{1}H NMR of titration experiment adding equivalents of diethylether to a solution of iodobenzene in $CDCl_3$. (**B**) Depiction of iodobenzene. Individual protons are marked according to the assignment in **A** and **D**. (**C**) Schematic representation of *meta*-iodo-phenylalanine in the intramolecular halogen bond forming system **1**. Individual chemical shift changes in **D** correspond to proton markers. (**C**) Comparison of the extend of shift change observed for iodobenzene against diethylether (shades of red) and protons of the halogen bond donor site in **1** (shades of blue). Although the halogen bonds forming in both systems are of similar strength, chemical shift changes are significantly larger upon the incorporation into the cooperatively folding system **1** in which the formed halogen bond contributes to the extent of folded complex.

2.4 Variable temperature experiments

2.4.1 Amide Temperature coefficient

Amide proton temperature coefficients were obtained by running ^{1}H-NMR experiments ranging from -45°C/228K to +75°C/348K, using 20% DMSO-d_6 in CD_2Cl_2 where the DMSO-d_6 deuterium signal was used as lock signal. A Varian 400MHz spectrometer equipped with an OneNMR probe was used. To avoid errors introduced by the temperature dependence of the chemical shift of a reference substance, the spectra were referenced to the transmitter nucleus frequency (^{1}H) (Agilent VnmrJ commands: `solvent='';setref(tn)`). The observed linear shift change of all amide proton signals with $R^2 \geq 0.99$ allowed to deduce the temperature coefficient as:

$$\Delta\delta_{NH}/\Delta T = \frac{\delta_{T,high} - \delta_{T,low}}{T_{high} - T_{low}} \ . \qquad (S3)$$

Coefficients are traditionally given as absolute values in ppb·K^{-1}.[2] $\Delta\delta_{NH}/\Delta T \leq 3$ indicate a strong intramolecular hydrogen bond for the corresponding amide proton. Values 3-5 ppb·K^{-1} indicate an equilibrium between the NH with an intramolecular hydrogen bond acceptor and its surrounding solvent, whereas coefficients ≥ 5 indicate solvent exposed protons.[3] Results are summarized in

Tables S6-S7. Comparing amide temperature coefficient between **1** and **2** revealed an increase in hydrogen bond lattice strength in **1**. This effect becomes more evident the further away from the β-turn. Interstrand hydrogen bonding amide NHs (blue) and solvent exposed NHs (red) are depicted in Figure S9. A comparison of shift changes, as $\Delta\delta_{NH}/\Delta T$ and $\Delta\Delta\delta_{NH}/\Delta T$ is depicted in Figure S10 and Figure S11.

Table S6: Amide proton temperature coefficients $\Delta\delta_{NH}/\Delta T$ (ppb·K^{-1}) for **1** in 20% DMSO-d_6 in DCM-d_2. Spectra are referenced to the transmitter nucleus frequency (^{1}H). Shifts (ppm) differ thereof from standard referenced spectra.

**Temp. °C**	**Leu^1 NH**	**Thr^2 NH**	**Phe(I)^3 NH**	**Glu^4 NH**	**Gly^6 NH**	**Lys^7 NH**	**hmSer^8 NH**	**Ser^9 NH**	**Ile^{10} NH**	**C-terminal NH$_a$**	**C-terminal NH$_b$**
75		2.462	2.635	2.997	2.825	2.516	2.707		2.264		
65	2.615	2.501	2.675	3.039	2.857	2.537	2.753	2.615	2.298	1.131	1.960
55	2.667	2.542	2.717	3.085	2.891	2.564	2.804	2.652	2.335	1.191	2.016
45	2.718	2.587	2.762	3.132	2.930	2.587	2.859	2.692	2.376	1.251	2.060
35	2.770	2.632	2.805	3.177	2.968	2.612	2.915	2.733	2.435	1.318	2.107
25	2.822	2.678	2.851	3.221	3.008	2.636	2.978	2.773	2.467	1.385	2.152
15	2.876	2.730	2.900	3.266	3.056	2.661	3.045	2.818	2.523	1.462	2.201
5	2.930	2.783	2.951	3.309	3.100	2.686	3.115	2.863	2.575	1.543	2.248
-5	2.986	2.837	3.000	3.346	3.145	2.711	3.188	2.910	2.631	1.624	2.293
-15	3.043	2.894	3.045	3.382	3.198	2.737	3.263	2.959	2.690	1.710	2.338
-25	3.098	2.955	3.098	3.413			3.337	3.012		1.792	2.382
-35				3.428							2.417
-45				3.463							
R^2	0.99	0.99	0.99	0.99	0.99	0.99	0.99	0.99	0.99	0.99	0.99
**$\Delta\delta_{NH}/\Delta T$**	**5.4**	**4.9**	**4.7**	**3.9**	**3.7**	**2.5**	**6.5**	**4.4**	**4.9**	**7.3**	**4.5**

Table S7: Amide proton temperature coefficients $\Delta\delta_{NH}/\Delta T$ (ppb·K^{-1}) for **2** in 20% DMSO-d_6 in DCM-d_2. Spectra are referenced to the transmitter nucleus frequency (^{1}H). Shifts (ppm) differ thereof from standard referenced spectra.

**Temp. °C**	**Leu^1 NH**	**Thr^2 NH**	**Phe(I)^3 NH**	**Glu^4 NH**	**Gly^6 NH**	**Lys^7 NH**	**Nle^8 NH**	**Ser^9 NH**	**Ile^{10} NH**	**C-terminal NH$_a$**	**C-terminal NH$_b$**
75			2.717	3.101	2.905	2.546	2.690		2.334		
65		2.662	2.777	3.153	2.949	2.566	2.756		2.372	1.076	2.088
55		2.705	2.802	3.193	2.989		2.804		2.415	1.120	2.143
45		2.757	2.859	3.241	3.037	2.623	2.868	2.842	2.459	1.184	2.202
35	2.696	2.807	2.926	3.289	3.090	2.640	2.940	2.896	2.511	1.248	2.265
25	2.752	2.868	2.992	3.341	3.146	2.670	3.024	2.949	2.578	1.317	2.319
15	2.858	2.923	3.036	3.383	3.202	2.693	3.105	2.999	2.637	1.388	2.377
5	2.878	2.985	3.098	3.429	3.263	2.724	3.189	3.051	2.707	1.474	2.435
-5	2.934	3.049	3.152	3.468	3.322	2.757	3.271	3.091	2.778	1.559	2.459
-15	3.006	3.104	3.205	3.502	3.378	2.785	3.349			1.645	2.529
-25	3.061	3.160	3.248	3.533	3.426		3.414			1.727	2.580
-35	3.137		3.296	3.555					2.973	1.820	2.616
-45									3.042		2.651
R^2	0.99	0.99	0.99	0.99	0.99	0.97	0.99	0.99	0.99	0.99	0.99
**$\Delta\delta_{NH}/\Delta T$**	**6.1**	**5.7**	**5.4**	**4.3**	**5.3**	**2.7**	**7.5**	**5.0**	**6.0**	**7.6**	**5.2**

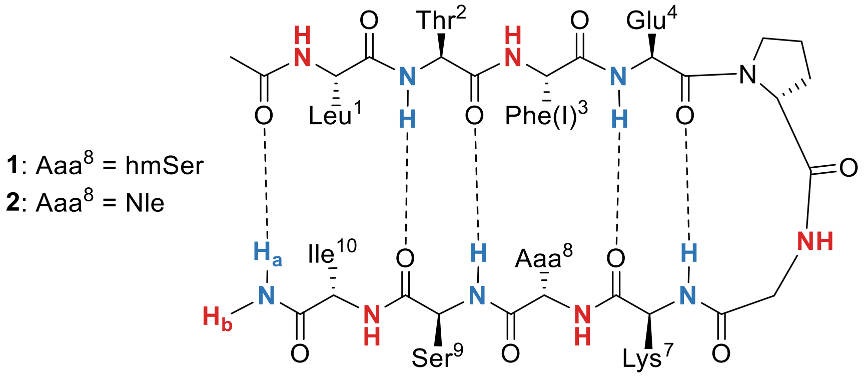

Figure S9: Structures of **1** and **2** with highlighted interstrand hydrogen bonds and amide NHs not involved in the hydrogen bond lattice. Interstrand hydrogen bond forming NHs are colored blue. Solvent exposed NHs are colored red

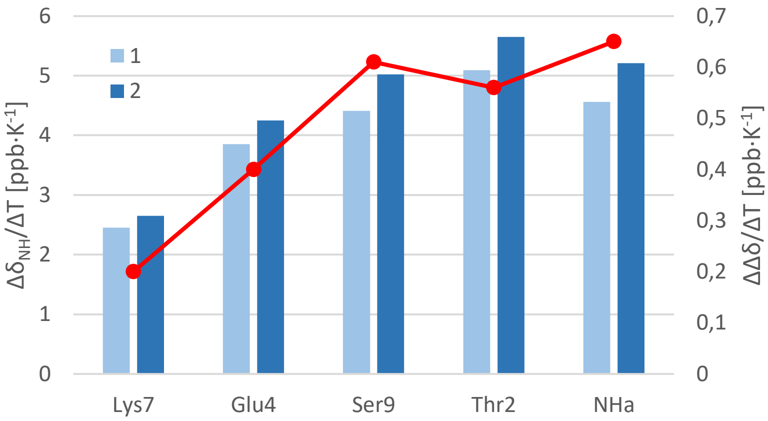

Figure S10: Comparison of amide temperature coefficient of **1** (bright blue) and **2** (dark blue) of amino acids involved in interstrand hydrogen bonding and sorted from the β-turn region to the termini (left to right). Difference in temperature coefficients is depicted as red interconnected dots. An overall trend for weaker hydrogen bonds towards the termini is visible whereat **2** shows a steeper increase of amide coefficient compared to **1** observable as the increase in ΔΔδ/ΔT.

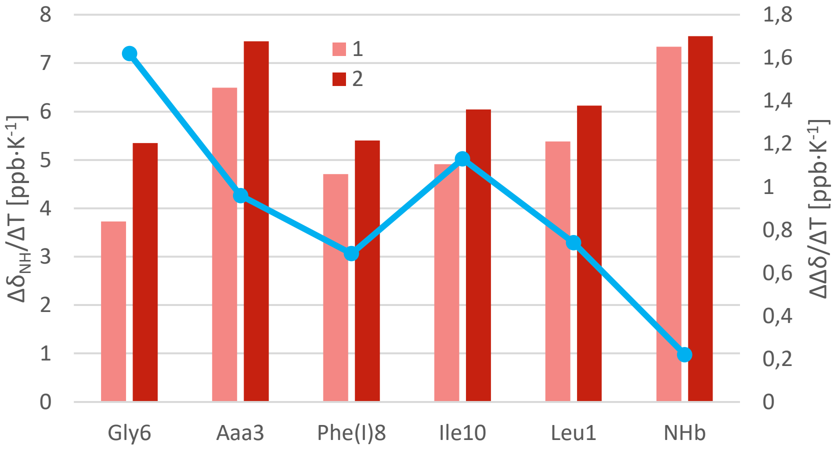

Figure S11: Comparison of amide temperature coefficient of **1** (bright red) and **2** (dark red) of amino acids not involved in interstrand hydrogen bonding and sorted from the β-turn region to the termini (left to right). Difference in temperature coefficients is depicted as blue interconnected dots.

2.4.2 Melting curve analysis based on H_α shifts

The H_α chemical shift is commonly used as thermodynamic determinant of the change in folding/unfolding equilibrium upon change of the surroundings. Following the NMR chemical shift change of these proton signals over a large temperature range can provide a sigmoidal curve, known as the melting curve. The folded and unfolded states are detectable for large systems e.g. a globular proteins, where the tertiary structure is held together by a multitude of non-covalent interactions. For peptides, this curve is broad and shifted to lower temperatures as fewer interactions cooperate to stabilize their native structure. Predominantly the low temperature plateau is not reached since the peptides stabilizing factors won't overcome internal entropy. The data obtained from NMR, CD, fluorescence and Raman IR experiments fit to a two-state transition as a function of temperature.[4] Extrapolating experimental data to an assumed steady state value for the folded peptide, in most cases, derives constants describing the traditional sigmoidal function. Even though the final shift is roughly estimated, the first order derivative of data points obtained from the curve fit will indicate the inflection point, T_m that usually lies in a temperature range covered by experimentally acquired data points.[5] Relative comparison of T_m between two similar peptides gives an estimate of overall stability difference in the measured solvent reflecting quantity and strength of contributing structure-stabilizing factors.

We followed shift change for H_α protons over a temperature range of 130°C (-45°C/228K -> 75°C/348K) starting with the lowest temperature in a pre-cooled NMR probe. The temperature was increased stepwise in intervals of 10°C followed by a 15 min temperature equilibration time. Sample preparation and referencing was described earlier. Peak shifts were deduced at the maximum height of H_α signals in ^{1}H-NMR spectra at all temperatures. Overlap and peak broadening hampered precise deduction of chemical shifts and heavily affected signals were skipped. Experimentally determined shift values (δ_{obs}) were corrected to a standard ppm scale by referencing δ_{obs} to ^{1}H-shifts referenced on the solvent peak recorded at 25°C/298K. Corrected shift values ($\delta_{H\alpha}$) are listed in Table S8 for **1** and Table S9 for **2**.

Table S8: Shift changes of H_α for **1** recorded in 20% DMSO-d_6 in DCM-d_2. Recorded spectra are referenced to the transmitter nucleus frequency (^{1}H). Deduced shifts (ppm) were corrected to standard referenced values using shifts recorded at 298K with solvent peak referencing. δ_U is the terminal unfolded shift obtained from curve fitting.

**Temp**	**$\delta_{H\alpha}$ [ppm]**										
K	**Leu^1**	**Thr^2**	**Phe(I)^3**	**Glu^4**	**Pro^5**	**Gly^6**		**Lys^7**	**hmSer^8**	**Ser^9**	**Ile^{10}**
348.15			4.789	4.581	4.314	3.852	3.806	4.410	4.475	4.434	4.288
338.15			4.793	4.574	4.314	3.845	3.797	4.406	4.473	4.433	4.282
328.15	4.375	4.270	4.798	4.567	4.312	3.841	3.791	4.398	4.471	4.431	4.279
318.15	4.377	4.272	4.804	4.561	4.313	3.837	3.781	4.387	4.469	4.432	4.276
308.15	4.379	4.279	4.811	4.556	4.316	3.833	3.772	4.373	4.469	4.431	4.272
298.15	4.380	4.280	4.820	4.550	4.320	3.830	3.760	4.360	4.470	4.430	4.270
288.15	4.386	4.286	4.830	4.545	4.325	3.827	3.746	4.345	4.472	4.430	4.266
278.15	4.390	4.287	4.842	4.540	4.335	3.825	3.732	4.333	4.476	4.429	4.261
268.15	4.403	4.303	4.854	4.534	4.343	3.824	3.714	4.329	4.483	4.427	4.258
258.15	4.413	4.314	4.867	4.530	4.352	3.826	3.696	4.316	4.493	4.424	4.247
248.15	4.413	4.326	4.887	4.523	4.362	3.828	3.676	4.301	4.504	4.420	4.246
238.15	4.426	4.334	4.901		4.366	3.830	3.651	4.281	4.475	4.434	4.232
228.15	4.435	4.338	4.915					4.253			4.217
**δ_U**	3.69	4.26	4.70	5.38	4.31	3.90		4.96	4.47	4.44	4.30

Table S9: Shift changes of alpha protons for **2** recorded in 20% DMSO-d_6 in DCM-d_2. Recorded spectra are referenced to the transmitter nucleus frequency (^{1}H). Deduced shifts (ppm) were corrected to standard referenced values using shifts recorded at 298K with solvent peak referencing. δ_U is the terminal unfolded shift obtained from curve fitting.

**Temp**	**$\delta_{H\alpha}$ [ppm]**										
K	**Leu[1]**	**Thr[2]**	**Phe(I)[3]**	**Glu[4]**	**Pro[5]**	**Gly[6]**		**Lys[7]**	**Nle[8]**	**Ser[9]**	**Ile[10]**
348.15			4.857	4.643	4.316						4.326
338.15	4.447		4.868	4.643	4.319			4.440		4.477	4.332
328.15	4.454		4.877	4.642	4.324	3.859	3.789	4.446		4.478	4.330
318.15	4.457	4.359	4.890	4.640	4.322	3.859	3.771	4.461	4.400	4.477	4.336
308.15	4.467	4.363	4.903	4.640	4.322	3.859	3.761	4.469	4.396	4.488	4.340
298.15	4.48	4.36	4.92	4.64	4.32	3.86	3.75	4.48	4.39	4.50	4.34
288.15	4.491	4.365	4.939	4.640	4.316	3.862	3.740	4.495	4.411	4.514	4.348
278.15	4.502	4.378	4.957	4.637	4.318	3.862	3.727	4.507	4.417	4.521	4.344
268.15	4.520	4.390	4.975	4.632	4.304	3.864	3.715	4.530	4.430	4.528	4.347
258.15	4.523	4.401	4.994	4.628	4.295	3.866	3.701	4.544	4.439	4.533	4.344
248.15	4.533	4.413	5.011	4.622	4.283	3.869	3.689	4.561	4.450	4.540	4.343
238.15	4.543	4.422	5.024	4.618	4.275	3.859	3.789	4.571	4.461	4.546	4.338
228.15		4.432	5.046		4.263	3.859	3.771	4.578	4.469	4.541	4.320
**δ_U**	3.62	4.35	4.78	4.85	4.32	3.85		4.19	4.39	4.47	4.35

Chemical shifts at unfolded conformation (δ_U), representing a random coil state, were estimated by fitting observed shifts to a two state melting function as proposed by Munekata *et al.*[6]

$$\delta_{obs} = \delta_U + \left\{ \frac{\delta_F - \delta_U}{1 + exp\left[-\frac{\Delta H_m}{R} * \left(\frac{1}{T} - \frac{1}{T_m}\right)\right]} \right\} \qquad (S4)$$

With δ_F and δ_U as the maximum chemical shifts for fully folded and unfolded states, respectively. ΔH_m is the enthalpy change upon unfolding at the transition temperature T_m. Factors for heat capacity were not included as changes in exposed surface area upon unfolding of short peptides is expected insignificant. This initial fit served the purpose to acquire an estimated random coil value (δ_U) for individual amino acids in the used solvent system. Both linear peptides showed reduced shift change at higher temperatures indicating the plateau for unfolded, random coil shifts. Therefore, determining δ_U based on experimental shift values gave reliable results independent from exact fitting of other variables. Non-linear least-squares curve fitting procedure was performed applying an *in-house* MatLab® script made available online (https://github.com/stepei/meltcurvefit).

Difference of obtained random coil values (δ_U) and Hα shifts for each amino acid were further used as the square-root-difference, $\Delta\delta_{H\alpha}^T$, per temperature, *T*.

$$\Delta\delta_{H\alpha}^T = \sqrt[2]{(\delta_{H\alpha}^T - \delta_U)^2} \qquad (S5)$$

The overall shift range of individual H_αs has little information about the conformational change, whereas the change in shift rate is indicative for the peptides thermodynamic behavior. Simply taking the RMS of all amino acids per temperature would result in a pooled curve dominated by the amino acid with the largest shift range. Therefore we applied a Hα shift-range rescaling of individual amino acids by z-score normalization.[7] This allowed pooling of shifts to generate an overall melting curve descriptive for the peptides backbone stability. Normalized values (δ_{norm}^{T}) were calculated following

$$\delta_{norm}^{T} = \frac{\left(\Delta\delta_{H\alpha}^{T} - \bar{\delta}_{H\alpha}\right)}{\sigma_{H\alpha}} \tag{S6}$$

where $\bar{\delta}_{H\alpha}$ is the mean and $\sigma_{H\alpha}$ is the standard deviation of an amino acids Hα shift change, $\Delta\delta_{H\alpha}^{T}$, over the observed temperature range. Overall melting curve's data points for **1** and **2** were obtained by taking the arithmetic mean, δ_{av}^{T}, of normalized shift values for each temperature step.

$$\delta_{av}^{T} = \frac{1}{n}\sum_{i=1}^{n} \delta_{i,norm}^{T} \tag{S7}$$

Table S10: Averaged values representing the overall folding behavior of **1** and **2**.

**Temp**	$\boldsymbol{\delta_{av}}$	
K	**1**	**2**
348.15	-1.122	-1.135
338.15	-1.062	-1.125
328.15	-0.931	-1.021
318.15	-0.786	-0.934
308.15	-0.574	-0.750
298.15	-0.394	-0.599
288.15	-0.154	-0.369
278.15	0.095	-0.097
268.15	0.453	0.280
258.15	0.840	0.620
248.15	1.289	1.056
238.15	1.330	1.256
228.15	1.675	1.574

The thereof obtained overall thermodynamic data for **1** and **2** was further investigated to obtain descriptors of folding and unfolding behavior as well as the relative stability between the halogen bonding **1** and the reference **2**. For detail see thermodynamic analysis.

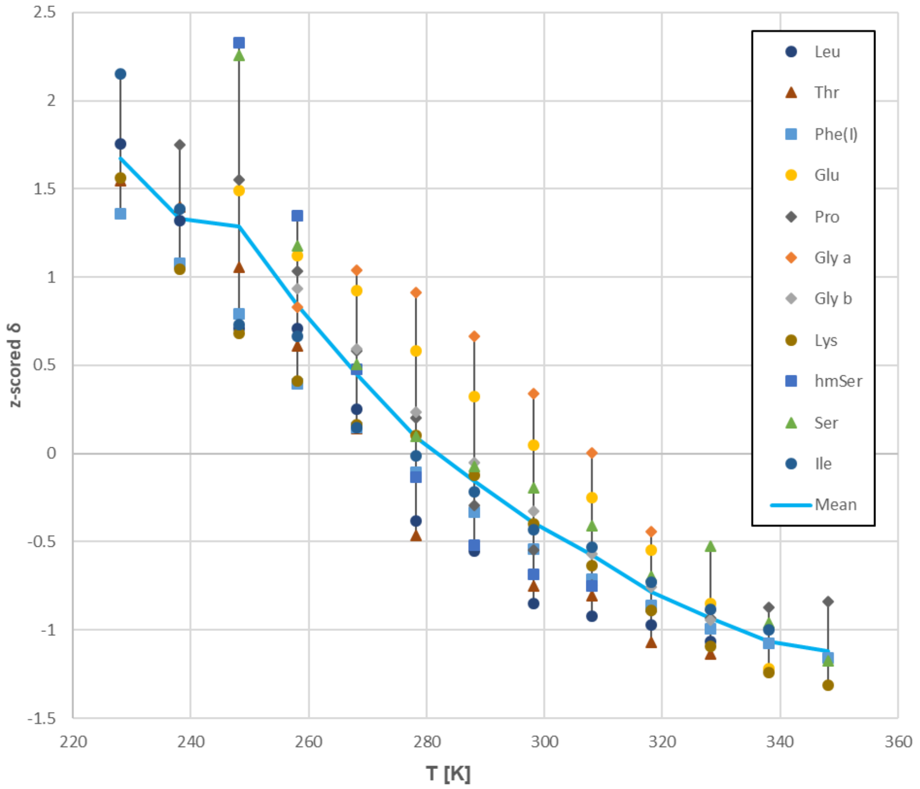

Figure S12: Z-score normalized chemical shift values for individual amino acids of **1**. Mean values of amino acid shifts per temperature are connected by a blue line representing the overall melting curve of **1**.

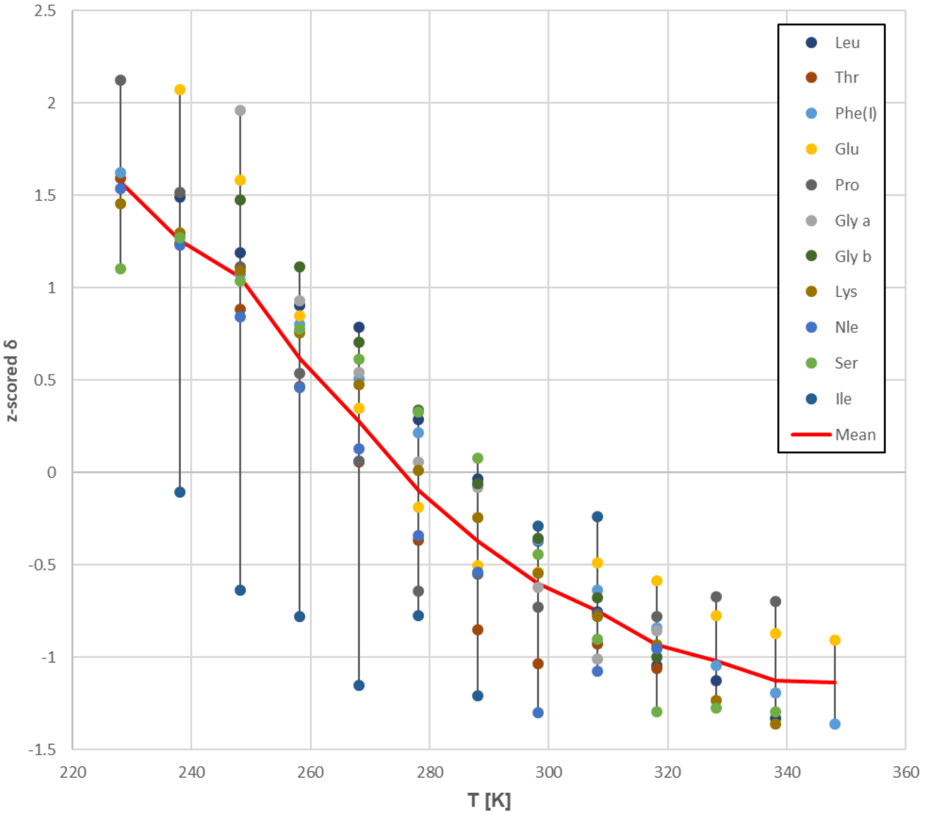

Figure S13: Z-score normalized chemical shift values for individual amino acids of **2**. Mean values of amino acid shifts per temperature are connected by a red line representing the overall melting curve of **2**.

3 Computational conformation sampling

3.1 Conformational search for NAMFIS analysis

A Monte Carlo conformational search was performed to generate the conformational ensemble for the NAMFIS analysis with 50.000 Monte Carlo steps using intermediate torsion sampling and a RMSD cut-off at 2.0 Å followed by Molecular Mechanics energy minimization using Macromodel as implemented in the Schrödinger Maestro package (v. 11.9.011). For each peptide independent conformational searches utilizing OPLS3e, OPLS and AMBER* as force fields and the implicit solvation model GB/SA for Water and $CHCl_3$. A Polak-Ribière type conjugate gradient (PRCG) algorithm was performed for energy minimization with 5.000 iterative steps. All conformers within 42 kJ mol^{-1} from the global found minimum were saved. For **1**, constrained searches in the three force fields and both solvation models were performed as well. Constraints were applied due to lack of the halogen's σ-hole description factors or other parameters describing its attractive interaction towards a Lewis base in current force fields models. Without constraints, electron repulsion was observed regardless the angle of which a Lewis base and the halogen orient towards each. Distance constraint was set to 3.2 ± 0.3 Å that is 90% of the sum of the Van-der-Waals radii of iodine (198 pm) and oxygen (152pm) with a force constant of 500 kJ mol^{-1} $Å^2$. Angle constraint was set to 180° ± 18° between C-I···O with a 500 kJ mol^{-1} $Å^2$ force constant. After conformational searches including constrained searches all ensembles were combined and redundant conformer elimination (RCE) was performed by comparison of heavy atom coordinates including hydroxyl groups. RMSD cut-off was set to 2.7 Å resulting in the final input ensembles of **1** and **2** for NAMFIS analysis. Results are summarized in Table S11.

Table S11: Results of Conformational searches for the NAMFIS analysis using Monte Carlo torsional sampling.

Compound	Force Field	Solvation model	Number of Conformers Total[a]	RCE 2.7 Å[b]
**1**	OPLS3e	Water	237	
	OPLS3e	$CHCl_3$	128	
	OPLS	Water	225	
	OPLS	$CHCl_3$	30	
	AMBER*	Water	467	
	AMBER*	$CHCl_3$	37	
**1** with C-I···O constraints[c]	OPLS3e	Water	139	
	OPLS3e	$CHCl_3$	36	
	OPLS	Water	31	
	OPLS	$CHCl_3$	57	
	AMBER*	Water	92	
	AMBER*	$CHCl_3$	158	
			Σ 1637	236
**2**	OPLS3e	Water	143	
	OPLS3e	$CHCl_3$	9	
	OPLS	Water	599	
	OPLS	$CHCl_3$	57	
	AMBER*	Water	299	
	AMBER*	$CHCl_3$	78	
			Σ 1185	176

[a] Total number of conformers found for each force field and solvation model [b] Redundant Conformer Elimination: Number of conformers obtained with a root-mean-square cut-off of 2.7 Å for heavy atoms [c] Constraints were applied to the distance and angle of the C-I···O halogen bond in halogen bonding **1**.

3.2 Conformational search for RDC analysis

To refine the sidechain geometry of the NAMFIS selected conformers of **1**, additional RDC based conformational analysis was performed. NMR experiments in isotropic and anisotropic conditions provided residual dipolar coupling constants for sidechain C-H bonds of the halogen bonding site as well as for backbone Cα-H of **1**. The experimental data was deconvoluted using the Fitter and RDC tools in MSpin v2.3.4-776. A conformer input ensemble was created using the outcome ensemble of the NAMFIS analysis. Individual outcome conformers were imported into Schrödinger's Maestro and a restrained conformational search was run. ϕ-, ψ- and ω-torsional angles were constrained as they were selected by NAMFIS with a 500 kJ mol^{-1} force constant. Rotation of all sidechains was unrestrained and sampled by a Monte Carlo torsional sampling with 10.000 steps, a 42 kJ mol^{-1} energy window and an RMSD cutoff of 1.5 Å. The conformational search was performed with the force field AMBER* in two implicit solvation models (Water and $CHCl_3$). Due to the lack of descriptors for the σ-hole and the corresponding electrostatic attraction in current force fields, constrained searches were performed to also sample halogen-bonded conformers. Two types of halogen-bond constrained conformational searches applying different, hard and soft, force constants to the C-I···O bond angle were run. This provided enhanced sampling of the positioning of the iodine in proximity of the oxygen corroborating with results by Ho *et al*. claiming a multi-angle behavior of halogen bond formation in biological systems.[8] Distance constraint for the I···O halogen bond was set to 3.2 Å representing a 90% distance of the sum of the Van-der-Waals radii of iodine (198 pm) and oxygen (152 pm) applying a force constant of to 1.000 kJ mol^{-1}. Constraints of 180° for the C-I···O bond angle with a zero tolerance

and a 100 kJ mol^{-1} force constant for soft halogen bond sampling and a 1.000 kJ mol^{-1} for hard sampling was applied. Settings regarding freezing of ϕ-, ψ- and ω-torsional angles were the same as for the non-halogen-bond constrained conformational search. Results of the 6 conformational samplings per constrained backbone conformations obtained from NAMFIS were pooled and the 3 lowest energy conformers of the halogen bond unrestrained-, soft- and hard-restrained runs were selected. In 3 cases soft and hard constraint gave the same halogen bond geometries of which one of them was eliminated. The resulting ensemble contained 24 conformers containing all backbone geometries as obtained from NAMFIS and samples the conformational space of Phe(I) and homo-met-Ser including halogen bond geometries. Relying on lowest energy conformers from AMBER* for conformer selection, is based in a necessary reduction of ensemble size due to the expensive deconvolution algorithm in MSpins Fitter relying on a brute-force combination search *vide infra*. For procedure on RDC data deconvolution using the NAMFIS based ensemble see section RDC analysis.

4 NAMFIS analysis

Computed input ensembles for **1** and **2** were used to determine individual interproton distances and dihedral angles. This dataset was deconvoluted against experimentally measured distances and coupling constants by the NAMFIS algorithm to determine solution ensembles. This was achieved following a previously described protocol.[9] Back calculation of dihedral angles deduced from the theoretical ensemble to scalar coupling constants $^{3}J_{NH,C\alpha H}$ was performed using a Karplus-equation optimized for peptides.[10a-b]

$$^{3}J_{HNH\alpha} = 9.4\ \cos^2\theta - 1.1\ \cos\theta + 0.4 \qquad (S8)$$

Where the angle θ is the dihedral angle between the amide N-H and the CαH given in radians. Distance information for the backbone and sidechains were used likewise, where it is to mention that flexibility of sidechains led to less good described orientations. The resulting solution ensemble therefore represents the backbone conformations, but sidechain positioning relies on fewer input data and thereto it is not possible to properly sample computationally. It was therefore further optimized using residual dipolar coupling (RDC) measurements *vide infra.* Every distance, *d* was assigned a general error estimate, *err* based on the distance it describes. Longer distances were assigned a larger error as the experimental determination of lower intensity NOE signals becomes less reliable (Table S12).[11] The limits of allowed deviation of experimental distances is weighted with positive and negative constraints. Upper and lower limits of the corresponding constraint will be calculated as

$$Limit_{lower} = d - r1 * err \qquad (S9)$$

$$Limit_{upper} = d + r2 * err \qquad (S10)$$

Where *d* is the NOE distance, *r1* was set to 3.0 for **1** and 1.8 for **2**, *r2* to 3.0 for **1** and 3.1 for **2** and *err* is the corresponding error based on size of *d*. Assigned error estimates for distances are summarized in Table S12. $^{3}J_{NH,C\alpha H}$ scalar coupling constants were assigned an error of 3.0 Hz for **1** and 1.5 Hz for **2**. Ensemble analysis was validated by removal of 10% experimental information without observing major change in molar fraction %. Results are shown in Tables S10, S11 and figures S10, S11.

Table S12: Error estimates used for experimentally determined interproton distances used in the NAMFIS analysis.

NOE distance (*x* in Å)	Error estimate (*err*)
x < 2.5	0.1
2.5 ≤ *x* < 3.5	0.2
3.5 ≤ *x*	0.3

Table S13: NAMFIS results show molar fraction of individual conformers. Molar fractions highlighted in bold are β-hairpin conformers.

1		2	
Conf. No.	Molar fraction %	Conf. No.	Molar fraction %
1	**16**	1	13
2	**3**	2	**24**
3	7	3	6
4	**13**	4	5
5	**24**	5	6
6	11	6	6
7	6	7	10
8	14	8	9
9	6	9	6
		10	**10**
		11	**5**
β-hairpin content	**56 %**		**39%**

Table S14: Experimentally determined (NOESY buildup) vs. back calculated interproton distances (Å) and coupling constants (Hz) of the backbone amide bond of specified amino acid residue for solution ensemble of **1**. For corresponding protons of individual distances see *Table S4*.

**Interproton distances [Å]**		**Coupling constants [Hz]**		
Exp.	Calc.	Exp.	Calc.	Residue
2.77	2.62	7.4	6.8	Glu^{4}
2.57	2.79	7.4	8.8	Lys^{7}
2.16	2.23	8.2	8.1	$Phe(I)^{3}$
2.31	2.34	7.4	7.9	$hmSer^{8}$
2.10	2.24			
2.63	2.64			
2.31	2.37			
2.54	2.59			
2.23	2.28			
2.56	2.48			
2.73	2.84			
2.61	2.65			
2.72	2.75			
2.84	2.84			
2.26	2.46			
3.66	3.48			
2.30	2.31			
2.55	2.59			
3.01	3.04			
2.26	2.37			
2.33	2.42			
2.64	2.65			
3.55	3.25			
2.57	2.67			
3.10	2.99			
3.33	3.20			
3.13	3.12			
RMSD distances: 0.11		*RMSD coupling constants: 0.80*		

*Table S15: Experimentally determined (NOESY buildup) vs. back calculated interproton distances (Å) and coupling constants (Hz) of the backbone amide bond of specified amino acid residue for solution ensemble of **2**. For corresponding protons of individual distances see Table S5.*

**Interproton distances [Å]**		**Coupling constants [Hz]**		
Exp.	Calc.	Exp.	Calc.	Residue
2.62	2.63	7.7	8.8	Glu[4]
2.83	2.84	7.9	8.5	Lys[7]
2.24	2.33	8.5	8.1	Phe(I)[3]
2.38	2.47	7.4	8.4	Nle[8]
2.62	2.62	7.6	7.6	Thr[2]
2.21	2.28			
2.53	2.70			
2.65	2.94			
2.68	2.74			
3.10	3.39			
3.74	3.79			
4.67	4.74			
2.54	2.63			
3.62	4.08			
2.22	2.29			
2.85	2.87			
2.70	2.87			
2.55	2.32			
2.86	2.88			
3.22	3.27			
3.34	3.40			
2.23	2.37			
2.32	2.50			
3.62	3.70			
2.31	2.44			
3.23	3.36			
2.70	2.78			
3.49	3.58			
3.20	3.23			
2.84	2.89			
2.93	2.87			
3.06	3.03			
3.26	3.32			
2.95	2.74			
3.03	2.96			
3.80	3.79			
3.00	2.87			
RMSD distances: 0.14		*RMSD coupling constants: 0.76*		

1: 16%

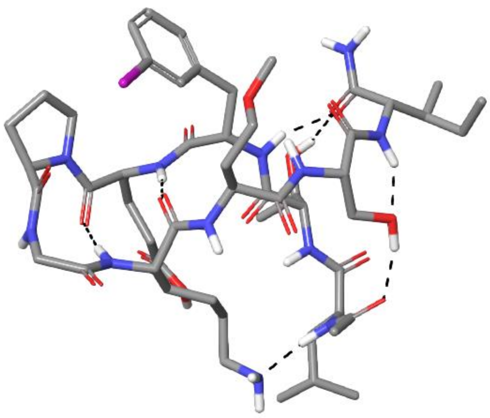

2: 3%

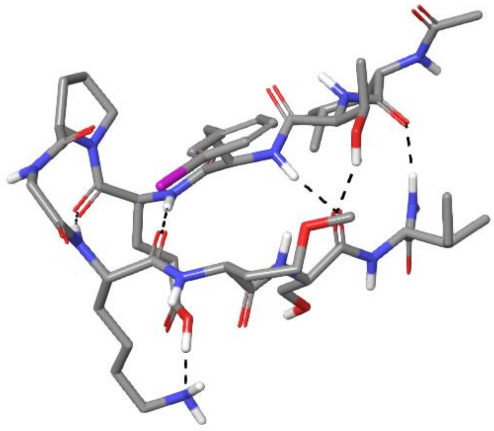

3: 7%

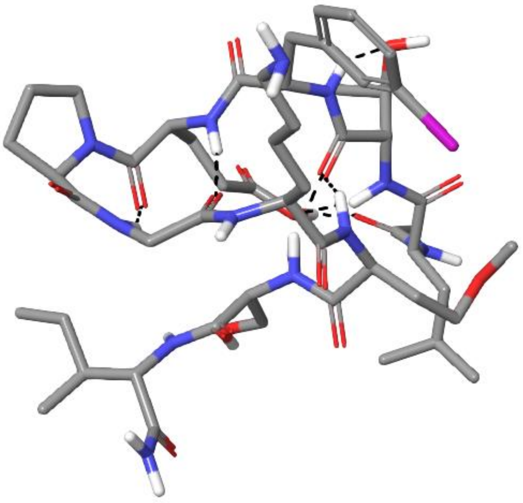

4: 13%

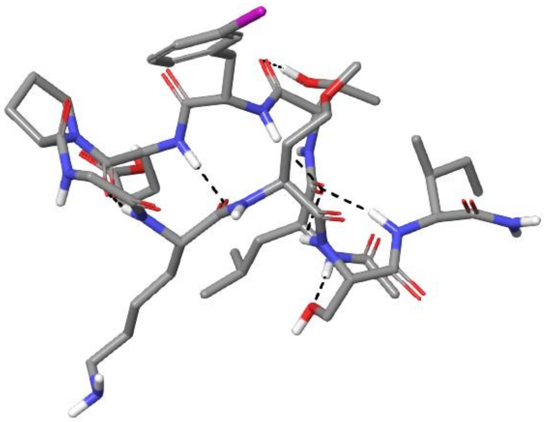

5: 24%

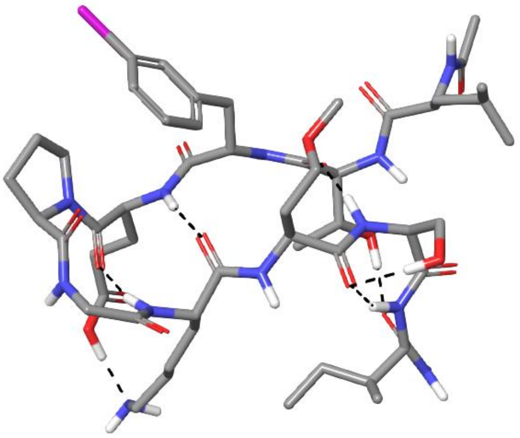

6: 11%

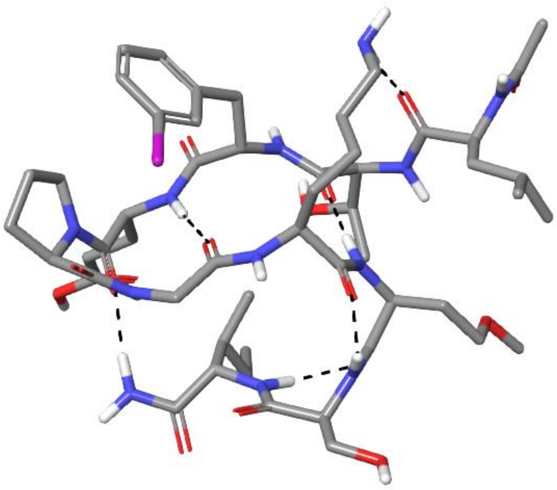

7: 6%

8: 14%

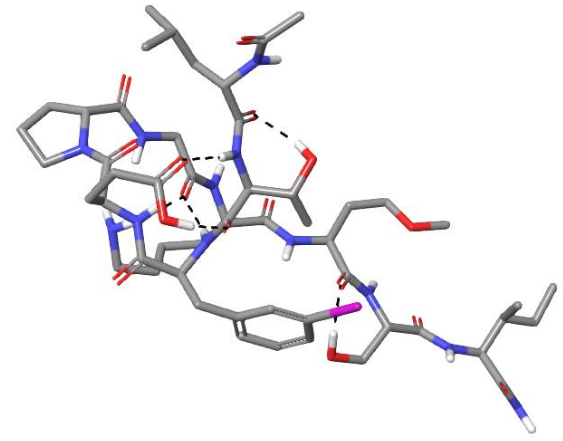

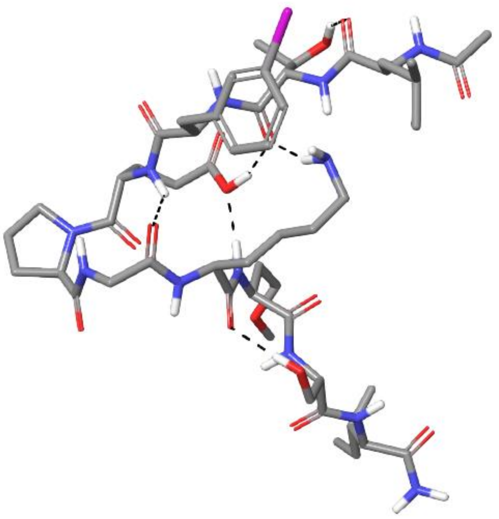

9: 6%

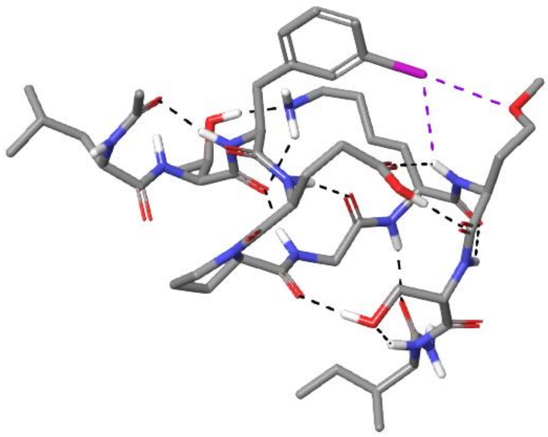

*Figure S14: The most populated solution conformations of **1** selected by NAMFIS. Hydrogen bonds are indicated as black dotted lines and Halogen bonds in purple. Non-polar protons are omitted for clarity.*

1: 13%

2: 24%

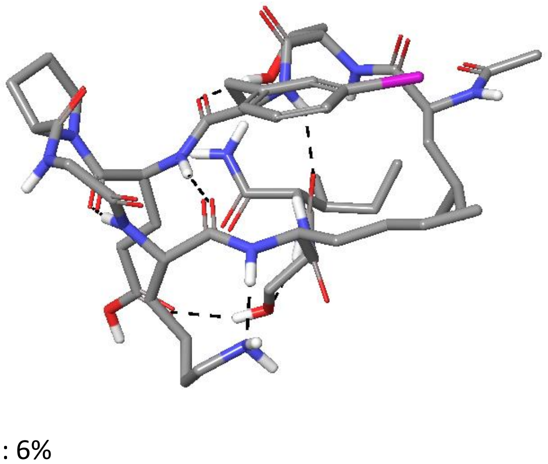

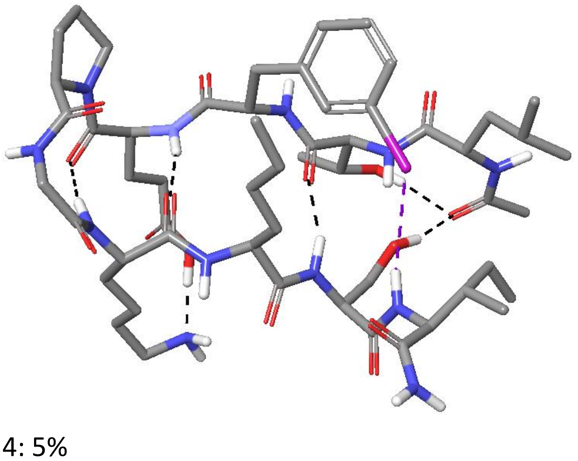

3: 6%

4: 5%

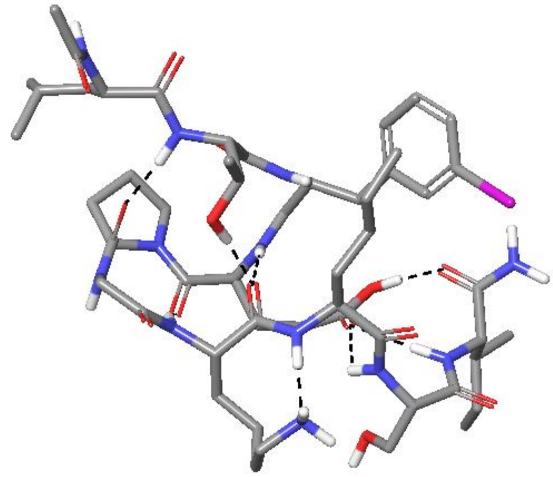

5: 6%

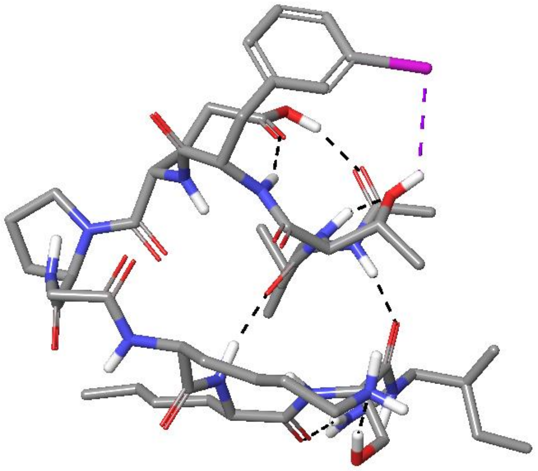

6: 6%

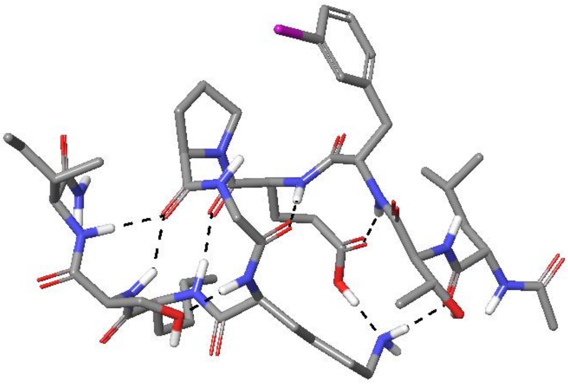

7: 10%

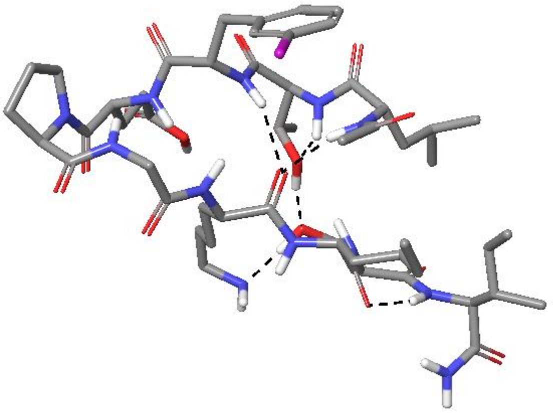

8: 9%

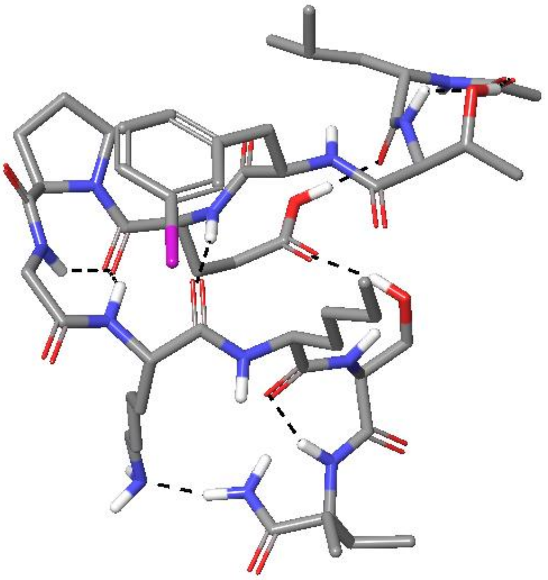

9: 6%

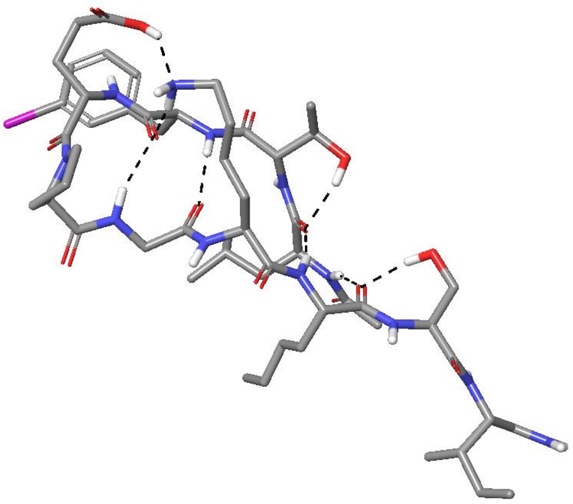

10: 10%

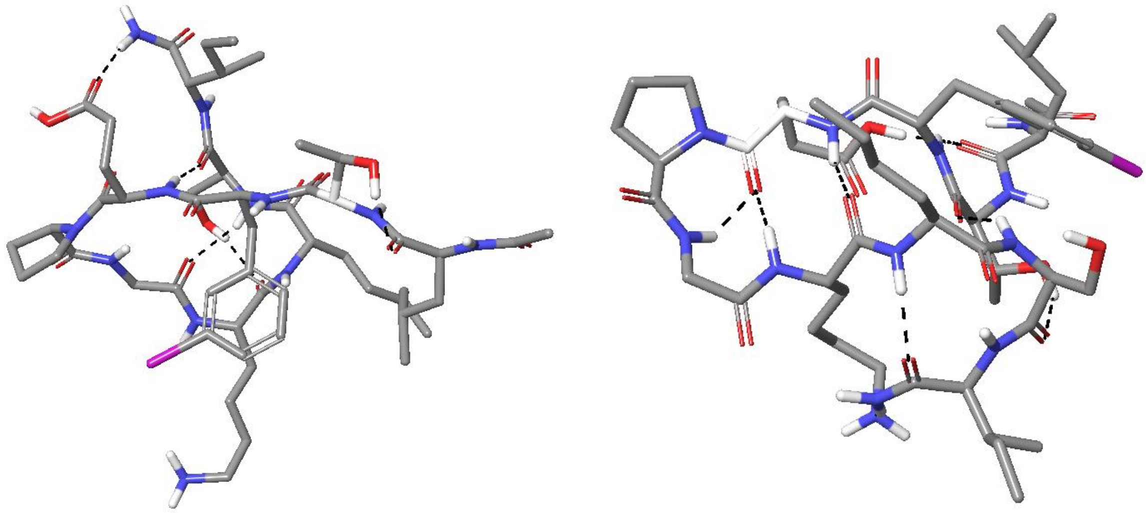

11: 5%

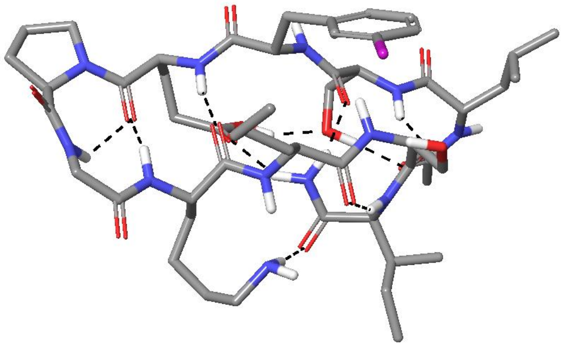

Figure S15: The most populated solution conformations of **2** selected by NAMFIS. Hydrogen bonds are indicated as black dotted lines. Non-polar protons are omitted for clarity.

Characterization of conformations

Classification of the conformers as β-hairpin was performed looking at the overall shape, organization of a cross-strand hydrogen bond network with correct formation of a ^{D}Pro^{5}-Gly^{4} type II' β-turn and backbone ϕ- and ψ-angles. Final classification is shown in Table S13. Rules for being considered as a β-hairpin conformer were set as follows:

1. Show ϕ- and ψ-angle geometry of ^{D}Pro^{4} and Gly^{6} associated with type II' β-turn formation

2. C_{α}-C_{α} distances of opposing amino acids associated with β-hairpin formation must be ≤ 6 Å on average

3. C(O)-N(H) distance of hydrogen bonding amino acids associated with β-hairpin formation must be ≤6Å on average

4. ϕ- and ψ-angles of amino acids in the antiparallel β-sheet plotted in a Ramachandran-plot must appear in the top left cubicle (ϕ: -180° - 0°; ψ: 0° – 180°)

β-Turn formation was assessed by measuring ϕ- and ψ-angles of the i+1 (^{D}Pro) and i+2 (Gly) amino acids and comparison to literature values for a type II' β-turn published by Jiménez.[12] Observed and literature reference values are listen in Table S16 for **1** and Table S17 for **2**.

Table S16: Comparison of ϕ- and ψ-angles of i+1 (^{D}Pro) and i+2 (Gly) amino acids in the turn of **1** with literature values related to a type II' β-turn.

**1**	**i+1**		**i+2**	
**Conf. No.**	**ϕ**	**ψ**	**ϕ**	**ψ**
1*	**67.5°**	**-100.3°**	**-80.1°**	**-17.7°**
2*	**55.5°**	**-110.0°**	**-85.3°**	**-8.8°**
3	74.8°	-60.3°	-115.6°	165.4°
4*	**64.7°**	**-102.0°**	**-115.7°**	**-0.2°**
5*	**65.2°**	**-104.2°**	**-88.2°**	**-15.1°**
6	75.8°	-47.9°	-114.2°	169.7°
7	67.6°	2.5°	179.9°	154.0°
8	60.4°	23.9°	103.6°	-101.4°
9	46.2°	-141.5°	-111.0°	-162.2°
**Type II' β-turn**	**60°**	**120°**	**-80°**	**0°**

Table S17: Comparison of ϕ- and ψ-angles of i+1 (^{D}Pro) and i+2 (Gly) amino acids in the turn of **2** with literature values related to a type II' β-turn.

**2**	**i+1**		**i+2**	
**Conf. No.**	**ϕ**	**ψ**	**ϕ**	**ψ**
1	**63.5**	**-111.7**	**-98.1**	**-8.3**
2*	**58.7**	**-108.7**	**-82.9**	**-14.6**
3	**62.4**	**-106.8**	**-103.1**	**2.3**
4	80.8	18.5	81.5	-91.8
5	64.2	55.6	79.0	-112.7
6	83.5	10.9	83.3	106.9
7	78.7	-66.4	-165.8	22.5
8	94.2	5.5	-162.6	106.3
9	94.7	-18.8	-176.1	149.7
10*	**83.4**	**-75.6**	**-154.5**	**33.3**
11*	**63.7**	**-85.3**	**-149.9**	**34.5**
**Type II' β-turn**	**60°**	**120°**	**-80°**	**0°**

Cross-strand C_α-C_α distances of Glu^4-Lys^7, $Phe(I)^3$-$hmSer^8$/Nle^8, Thr^2-Ser^9 and Leu^1-Ile^{10} were measured. Assessment of the hydrogen bonding network was based on interatomic distances of the carbonyl carbon C(O) and the amide nitrogen N(H) measured for proposed cross-strand hydrogen bonds (compare Figure S9). Occurrence of fraying is ubiquitous in all NAMFIS-selected conformers resulting in an overall increase in cross strand distances the further away from the center β-turn. As the classification into a β-hairpin considers the entire molecules shape, the average of a conformers cross-strand interatomic distance measures (C_α-C_α and C(O)-N(H)) must not exceed 6 Å to be considered a β-hairpin. Interatomic distances for all conformers are listed in Table S18 to Table S21.

Table S18: C_α-C_α interatomic distances for **1** measured between amino acids forming cross-strand hydrogen bonding pairs upon β-hairpin formation. Arithmetic means of distances must be ≤ 6 Å for the conformer to be considered for β-hairpin classification. Selected β-hairpin conformers are highlighted with an asterisk.

**1**	**C_α-C_α distance [Å]**				**Average**
**Conf. No.**	**Glu^4-Lys^7**	**Phe(I)^3-hmSer^8**	**Thr^2-Ser^9**	**Leu^1-Ile^{10}**	
1*	5.10	4.29	4.62	7.48	**5.37**
2*	5.01	4.46	5.39	6.09	**5.24**
3	6.43	5.40	5.80	9.26	6.72
4*	4.96	3.91	6.68	5.11	**5.17**
5*	4.96	4.41	4.54	7.68	**5.40**
6	6.47	6.15	8.22	9.68	7.63
7	6.90	7.48	8.61	15.19	9.55
8	4.92	6.93	10.79	14.42	9.26
9	6.46	7.89	10.05	12.50	9.22

Table S19: C_α-C_α interatomic distances for **2** measured between amino acids forming cross-strand hydrogen bonding pairs upon β-hairpin formation. Arithmetic means of distances must be ≤ 6 Å for the conformer to considered for β-hairpin classification. Selected β-hairpin conformers are highlighted with an asterisk.

**2**	**C_α-C_α distance [Å]**				**Average**
**Conf. No.**	**Glu^4-Lys^7**	**Phe(I)^3-hmSer^8**	**Thr^2-Ser^9**	**Leu^1-Ile^{10}**	
1	5.02	4.64	6.51	5.29	**5.37**
2*	4.91	4.43	5.38	6.24	**5.24**
3	4.84	5.00	9.35	13.61	8.20
4	5.58	7.83	4.38	5.04	**5.71**
5	4.59	8.42	10.25	15.09	9.59
6	5.34	7.01	6.41	7.29	6.51
7	5.24	4.22	5.89	11.94	6.82
8	7.27	9.20	8.17	9.52	8.54
9	6.94	5.11	8.41	11.18	7.91
10*	5.37	4.24	4.94	9.45	**6.00**
11*	5.25	4.19	5.16	5.18	**4.94**

Table S20: C(O)-N(H) interatomic distances for **1** measured between amino acids forming cross-strand hydrogen bonds upon β-hairpin formation. Arithmetic means of distances must be ≤ 6 Å for the conformer to be considered for β-hairpin classification. Selected β-hairpin conformers are highlighted with an asterisk.

**1**	**C(O)-N(H) distance [Å]**					**Average**
**Conf. No.**	**C(O) Glu^4 – N(H) Lys^7**	**C(O) Lys^7 – N(H) Glu^4**	**C(O) Thr^2 – N(H) Ser^9**	**C(O) Ser^9 – N(H) Thr^2**	**C(O) Ac – N(H) Ile^{10}**	
1*	3.88	3.91	4.09	5.19	9.07	**5.23**
2*	3.93	3.92	5.15	5.38	5.98	**4.87**
3	5.58	5.72	4.04	6.60	12.02	6.79
4*	3.77	3.78	5.98	5.94	6.06	**5.11**
5*	3.81	3.94	3.74	4.53	9.88	**5.18**
6	5.59	6.15	6.43	8.39	14.20	8.15
7	5.84	7.25	8.21	11.29	18.29	10.17
8	4.11	4.93	8.88	11.90	15.97	9.16
9	6.81	6.25	8.83	11.26	13.45	9.32

Table S21: C(O)-N(H) interatomic distances for **2** measured between amino acids forming cross-strand hydrogen bonds upon β-hairpin formation. Arithmetic means of distances must be ≤ 6 Å for the conformer to be considered for β-hairpin classification. Selected β-hairpin conformers are highlighted with an asterisk.

**2**	**C(O)-N(H) distance [Å]**					
**Conf.**	**C(O) N(H)**	**C(O) N(H)**	**C(O) N(H)**	**C(O) N(H)**	**C(O) N(H)**	**Average**
**No.**	**Glu^{4} Lys^{7}**	**Lys^{7} Glu^{4}**	**Thr^{2} Ser^{9}**	**Ser^{9} Thr^{2}**	**Ac Ile^{10}**	
1	3.87	3.92	5.74	4.45	8.34	**5.26**
2*	3.89	3.88	4.14	5.83	9.08	**5.36**
3	3.77	3.92	7.41	11.03	14.95	8.21
4	4.46	5.80	5.38	4.34	6.42	**5.28**
5	4.29	5.37	9.08	12.54	16.21	9.50
6	4.57	5.26	6.76	6.66	9.55	6.56
7	3.95	3.76	4.71	8.25	12.69	6.67
8	6.15	8.43	9.07	9.10	9.09	8.37
9	5.97	6.36	6.11	8.32	15.34	8.42
10*	3.91	4.02	3.83	5.55	12.52	**5.97**
11*	3.90	3.86	4.05	3.74	5.76	**4.26**

Finally, all ϕ- and ψ-angles of the backbone of **1** and **2** were deduced and plotted in a Ramachandran plot. Typically β-sheet angles appear somewhere in the top left cubicle (ϕ: -180° - 0°; ψ: 0° – 180°) depending on the twist of the obtained β-plane. Amino acids in the turn region (^{D}Pro and Gly) are not considered in the Ramachandran plot. Plots were analyzed by looking at outliers showing dihedral angles outside the favorable region for β-sheets, and how close the associated amino acid is to the turn. The closer an amino acid to the β-turn region the more crucial its effect on β-sheet propagation. This interpretation was used rather as an exclusion criterion more than a determination of β-hairpin formation. The Ramachandran plot mostly reflects if the backbones propagate as a planar amino acid strand. The more crucial formation of a β-turn and formation of a cross-strand hydrogen bonding network to adopt a β-hairpin conformation has been asses by the previous criteria. Ramachandran plot of individual conformers selected by NAMFIS are shown in Figure S16 for **1** and Figure S17 for **2**.

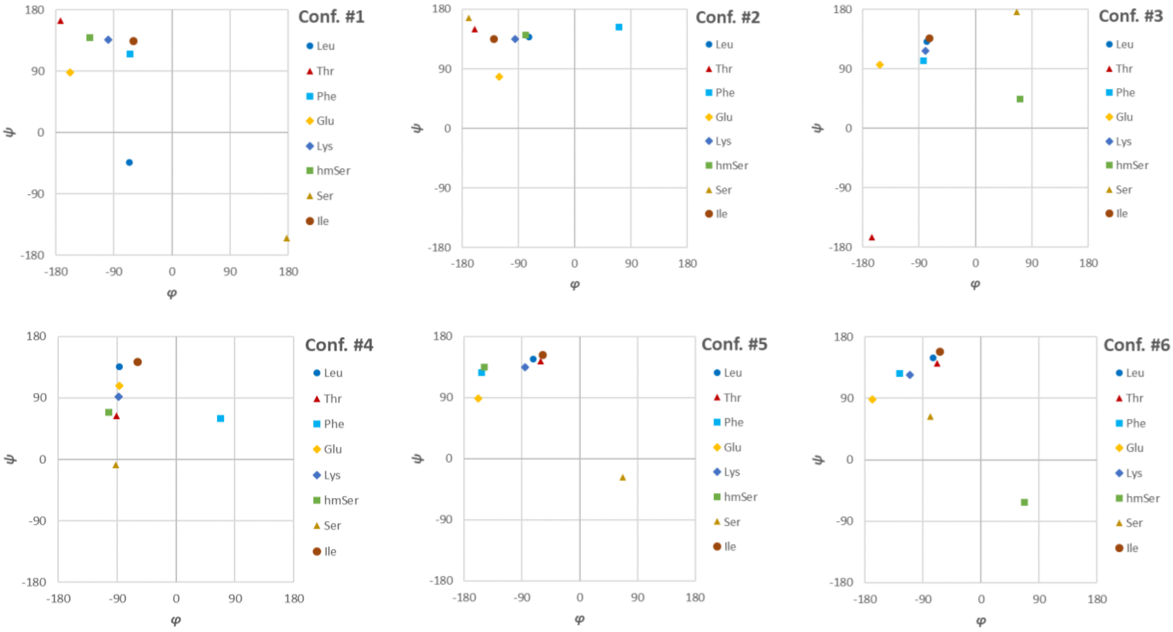

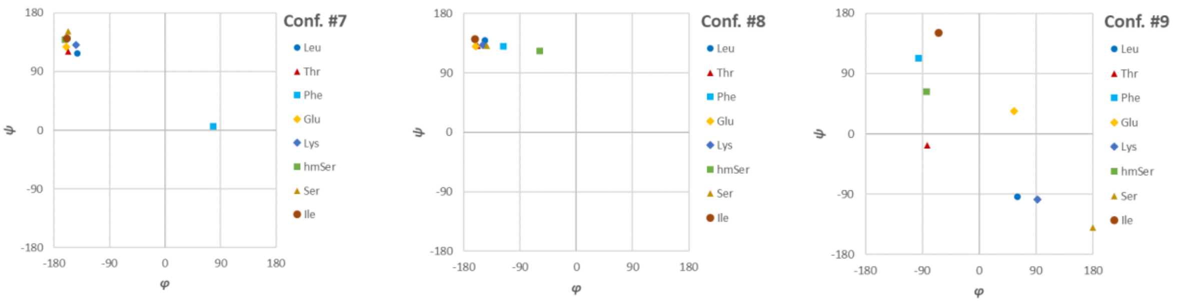

Figure S16: Ramachandran plot of individual conformers of **1** selected by NAMFIS.

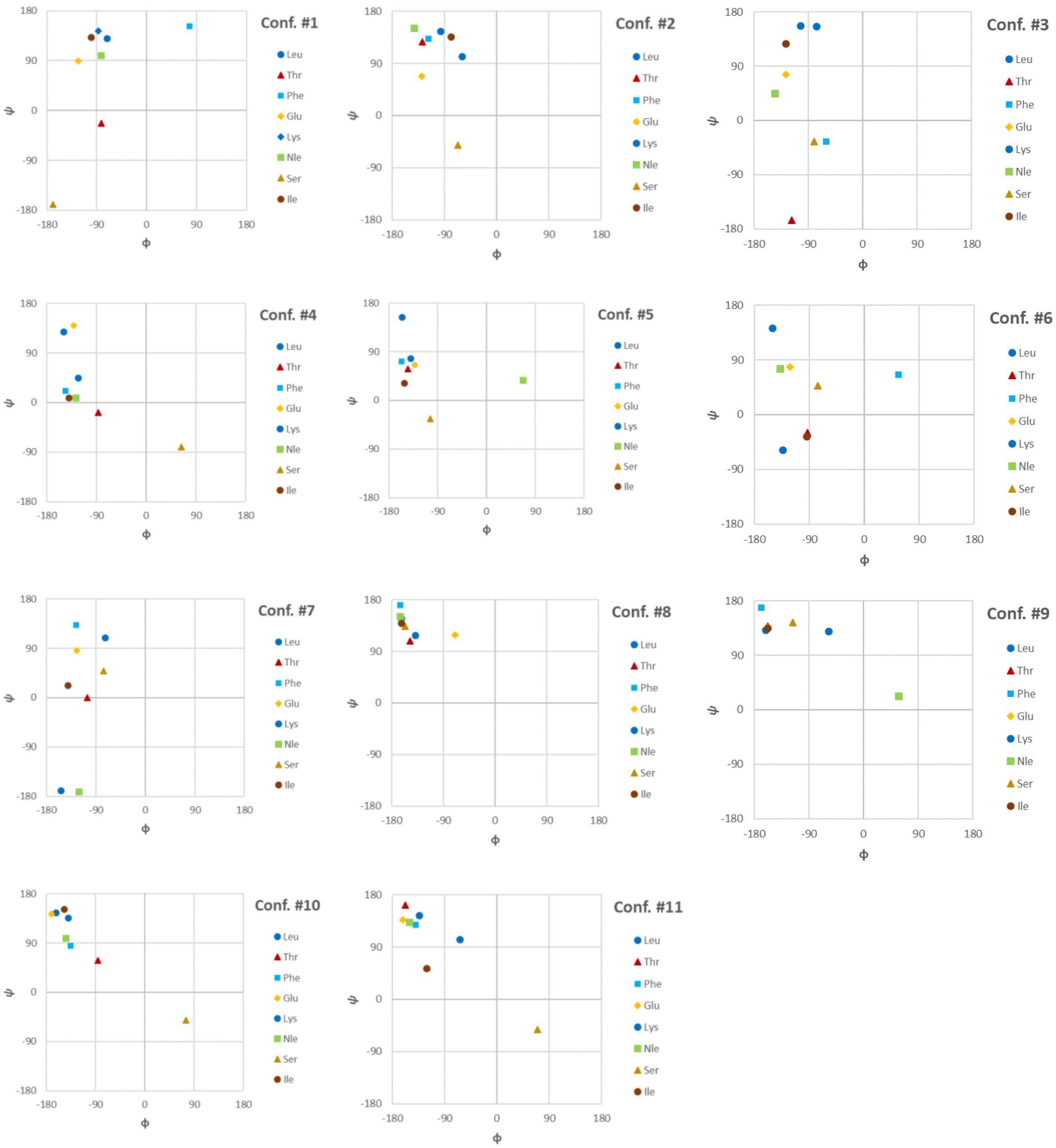

Figure S17: Ramachandran plot of individual conformers of **2** selected by NAMFIS.

5 RDC analysis

Residual dipolar couplings (RDC) were deduced as the difference (RDC = T - J) of the $^{1}J_{C\text{-}H}$ coupling constant in an anisotropic sample and observed total coupling $^{1}T_{C\text{-}H}$ in an isotropic sample. Obtained values for CH_2 and CH_3 were used as the half- and third-sum of its original value, respectively. For sample preparation, NMR experimental details and conformer ensemble preparation *vide infra.*

Table 22: RDC values measured as the difference (RDC = T – J) of the anisotropic C-H coupling constant to the isotropic of **1**. ^{a} represents the C-H vector *para* in respect to the iodine ^{b} represents the C-H vector *ortho* in respect to the iodine * Values are from a C-H$_n$ group (n > 1); obtained RDC values were divided by n.

#	*C-H vector* Residue	position	Group	Isotropic $^{1}J_{CH}$	Error	Anisotropic $^{1}T_{CH}$	Error	T - J RDC	Error
1	Phe(I)	α	CH	141.4	0.66	128.0	3.54	-13.4	4.20
2		β	CH_2^{*}	260.2	0.69	127.5	1.95	-67.9*	2.64
3		δ^{a}	CH	160.2	0.60	120.3	1.01	-39.9	1.61
4		δ^{b}	CH	165.3	0.70	109.7	0.67	-55.6	1.38
5		ε	CH	158.6	0.50	107.7	0.44	-50.9	0.94
6		ζ	CH	167.1	0.59	188.1	0.71	21.1	1.29
7	Glu	α	CH	138.9	0.68	196.4	1.69	57.5	2.37
8	Pro	α	CH	145.9	0.47	147.7	1.09	1.8	1.56
9	Gly	α	CH_2^{*}	280.6	0.41	292.6	0.55	6.1*	0.95
10	hmSer	γ	CH_2^{*}	283.9	0.27	286.8	0.87	1.5*	1.14
11		ε	CH_3^{*}	416.6	0.06	434.7	0.17	6.0*	0.23

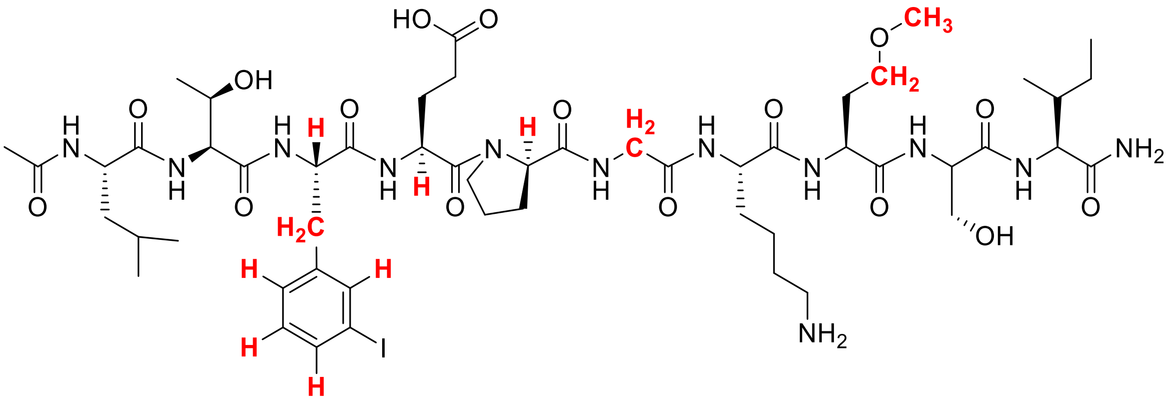

Figure S9: Depiction of deduced C-H dipolar couplings for **1** used for conformational analysis refining the position of the sidechains at position Phe(I)[8] and hmSer[3].

Experimental RDC data (D^{exp}) and the theoretical ensemble containing 24 conformers were imported into MSpin. The Fitter module of MSpin deconvolutes NMR observables into a series of discrete conformation contributions chosen form a presented ensemble. Each combination of n conformers is minimized by population weighing to get the best fit populations expressed as χ^2

$$\chi^2 = \sum_i \frac{\left(D_i^{exp} - \bar{D}_i^{calc}\right)^2}{\sigma_i^2} \qquad (S11)$$

where σ_i^2 is the associated error determined experimentally. This process is done iteratively increasing the number of possible outcome conformers (r) starting from 1. This avoids overfitting, *i.e.* the artificial

inclusion of minor populations to improve χ^2. The final score is given as AIC (Akaike information criterion) that includes the number of conformers, *r,* and minimized factor χ^2.

$$AIC = \chi^2 + 2(r-1) \tag{S12}$$

The calculation stops when combination of *r* conformers furnished a higher AIC value than the previous *r*-1 combination. Since all possible combinations *C* for *r* of *n* are tested following

$$C(n,r) = \frac{n!}{r!\,(n-r)!} \tag{S13}$$

where *r* is an increasing integer of selected conformers from the ensemble containing *n* elements, calculation steps for *r* increase exponentially. This brute force algorithm limits the size of *n* since MSpin cannot calculate combinations exceeding 20.000.000 steps. In our case ensemble *n* contained 24 elements (see Conformational search for RDC analysis). MSpin's Fitter module found no further decrease of AIC after *r* = 5 conforms (*C* = 42.504). Results of the MSpin Fitter deconvolution are summarized in Table S23, selected conformers are depicted in Table S24.

Table S23: Results of the RDC analysis using MSpins Fitter module. Molar fractions are given in %. Hairpin conformers are in bold and halogen bonding conformers are marked with *.

Conformation	Molar fraction %
1	**23**
2	**30***
3	11*
4	2
5	34*
AIC	8.5569
χ^2	0.5569
β-Hairpin content	53%
XB content	75%

1: 23%

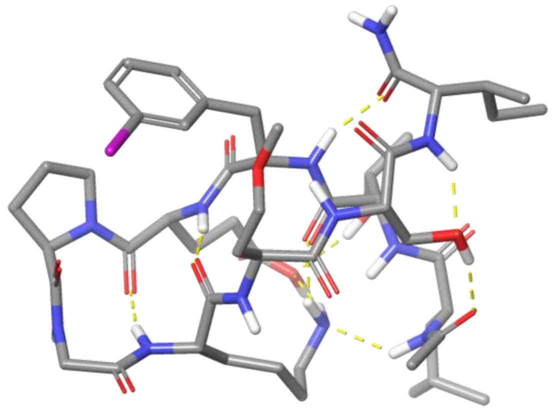

2: 30%

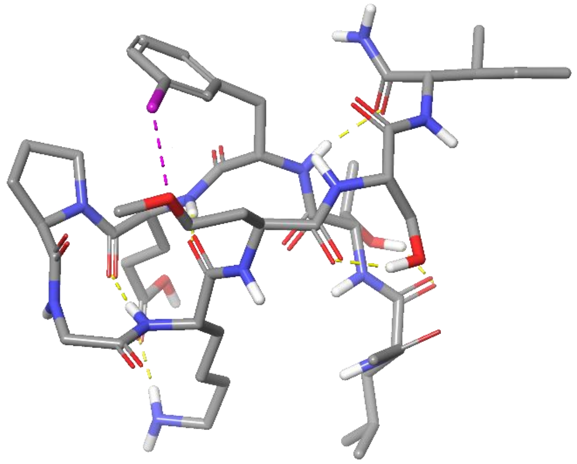

3: 11%

4: 2%

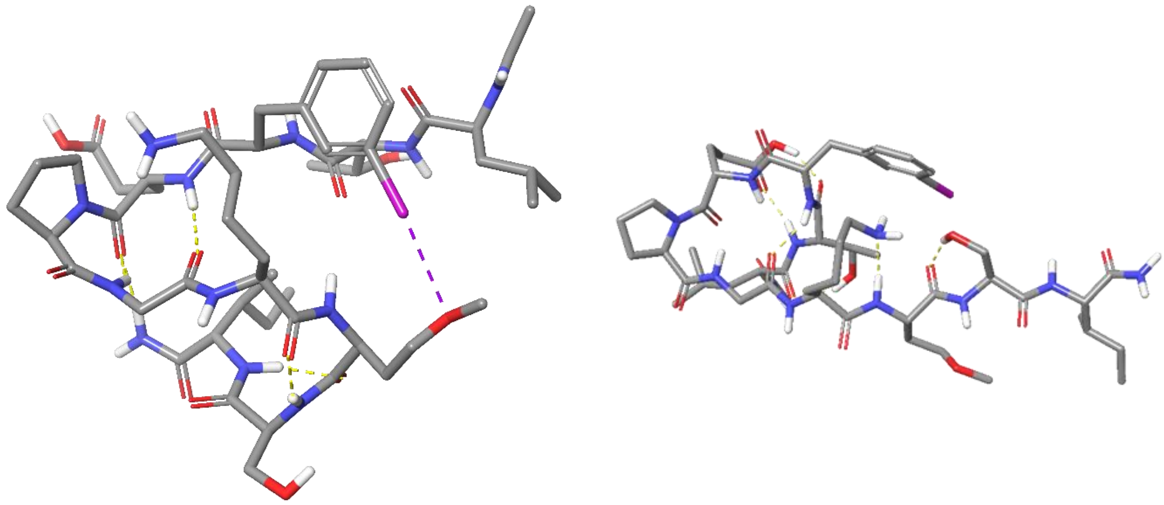

5: 34%

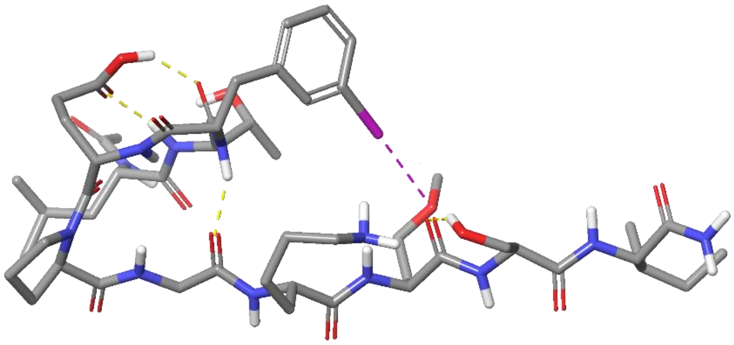

Table S24: Conformers and their assigned molar fractions of **1** found by RDC analysis using MSpin's Fitter module**.** Hydrogen bonds are depicted as yellow dotted lines. Halogen bonds are depicted as purple lines.

To get the SVD (Singular value decomposition) condition number, the Cornilescu quality factor *Q* and the GDO (Global degree of order), the 5 conformations selected by MSpin Fitter module were exported into a new input ensemble for MSpins RDC module. The same experimental data set was used applying a Levenberg-Marquadt SVD computation algorithm assuming a single tensor to fit populations. This iteratively re-optimized the calculated alignment tensor to gain populations weights best-fitting to the experimental data. To avoid getting trapped in a local minimum due to the non-linear character of the calculation, random sampling of initial populations was iterated 256 times.

Goodness of the fit is expressed in terms of the Cornilescu quality factor *Q*, a sort of scaled RMSD similar to Pearson's linear correlation factor, *R*.[13]

$$Q = \frac{RMS\left(\Delta\delta^{exp} - \Delta\delta^{calc}\right)}{RMS(\Delta\delta^{exp})} \qquad (S14)$$

Observed Q factor of 0.003837 for data fit was excellent and a SVD condition number of 4.028 being ≤ 20 indicated reliable alignment tensor determination. The GDO was back-calculated from the alignment tensor information and size of experimental RDC values to ~ 0.9% degree of alignment. This is in strong agreement with the type and amount of used alignment medium PBLG.[14] Comparison of back-calculated data using the Fitter module and the RDC module implemented in MSpin is shown in

Table S25. Back-calculated data as obtained from MSpins RDC module versus experimental data is plotted in Figure S18.

#	Exp. data [Hz]	Error [Hz]	Back-calculated data [Hz]	
			**Fitter module**	**RDC module**
1	-55.6	1.4	-55.66	-55.70
2	21.1	1.3	21.33	20.99
3	-39.9	1.6	-39.63	-39.65
4	-50.9	0.9	-50.86	-50.88
5	1.5	1.1	0.87	1.46
6	6.0	0.2	6.04	6.21
7	-13.4	4.2	-13.53	-13.62
8	-67.9	2.6	-67.93	-67.94
9	6.1	1.0	6.11	5.96
10	57.5	2.4	57.11	57.52
11	1.8	1.6	2.30	1.97
		**RMSD**	**0.2938**	**0.1445**

Table S25: Deconvolution of observed RDC data by NMR using two different algorithms implemented in MSpin, Fitter and RDC module. Goodness of the fit for both methods is indicated by RMSD.

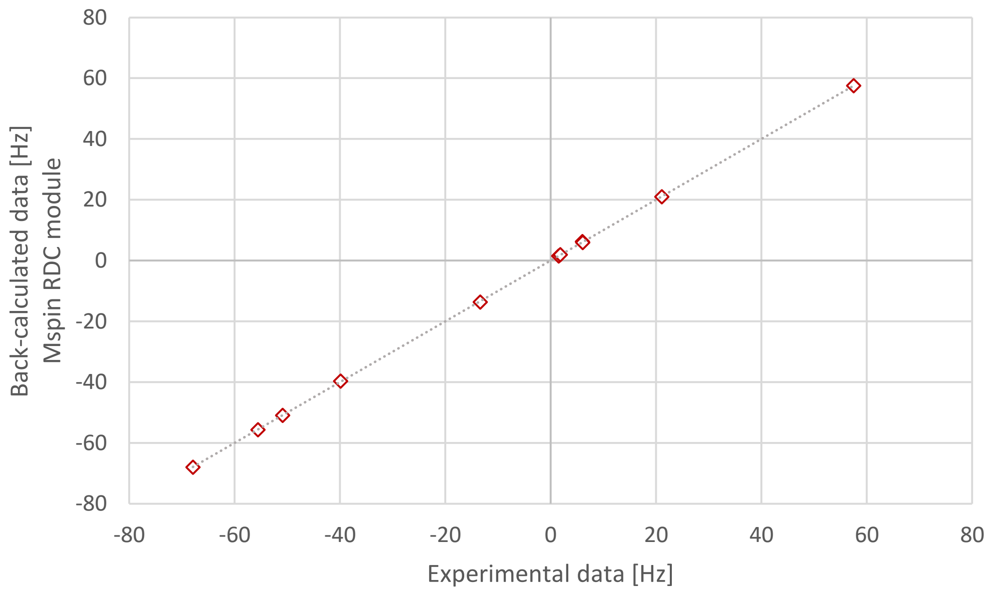

Figure S18: Plot of Back-calculated data obtained from data deconvolution by MSpins RDC module against the experimental RDC data obtained by NMR.

From Table S25 it is evident that the RDC module achieved a better population weighing than the Fitter module. This is probably due to rounding errors as the Fitter module only works with full percentage values in order to decrease the wall time of the rather expensive combinatorial calculation (see Table S26). Both algorithms operate independently and result in almost identical population balancing. We assume that the iterative process calculating best fit values for an increasing set of conformers implemented in the Fitter tool serves as good first estimate of conformational range while preventing data overfitting. The RDC tool fine adjusts population weights and give mathematical descriptors of

the alignment tensor and parameters for quality-of-fit assessment. Population weighing of the two algorithms is shown in Table S26.

Conformation	Populations	
	Fitter module	RDC module
1	23%	21.9%
2	30%	31.3%
3	11%	10.5%
4	2%	3.0%
5	34%	33.2%

Table S26: Comparison of resulting population fits of the two algorithms for the Fitter and RDC module implemented in MSpin.

6 Thermodynamic analysis

Obtained temperature dependent shift changes of H_α shifts have been normalized and combined as described in section 2.4.2 Melting curve analysis based on H_α shifts. Applying a least-squares curve fitting algorithm based on a literature protocol by Munekata *et al.*, we obtained the full two-state melting curves for **1** and **2**.[6]

Curve-fittings and all further described thermodynamic calculations are entirely performed by an in-house MatLab® script made available online at https://github.com/stepei/meltcurvefit.

$$\delta_{av} = \overline{\delta_U} + \left\{ \frac{\overline{\delta_F} - \overline{\delta_U}}{1 + exp\left[-\frac{\Delta H_m}{R} * \left(\frac{1}{T} - \frac{1}{T_m} \right) \right]} \right\} \qquad (S15)$$

Fitting equation S15 to the pooled experimental data (δ_{av}, see Table S10), yielded values for the mean unfolded shift ($\overline{\delta_U}$), folded shift ($\overline{\delta_F}$) and change in enthalpy (ΔH_m) at transition temperature (T_m) for both peptides. Fitted constants are listed in Table S27 and resulting curve-fits of experimental shift data are plotted in Figure S21 and Figure S22. Applying obtained values to equation S15 we could generate an extended melting curve data set for both peptides ranging from 100-600 K. An overlap of extended curves of **1** and **2** is shown in Figure S19 and represented as normalized plots in Figure S20. Discussion of the resulting parameters can be found in the main text.

Table S27: Obtained values for fitted parameters for **1** and **2** using a least-squares curve fitting protocol.

	$\overline{\delta_U}$	$\overline{\delta_F}$	ΔH_m [kJ mol^{-1}]	T_m [K]	RMSD
**1**	-1.46 ± 0.16	2.03 ± 0.18	23.9 ± 3.3	274.0 ± 2.5	0.054
**2**	-1.35 ± 0.06	1.93 ± 0.09	26.4 ± 1.7	267.6 ± 1.3	0.028

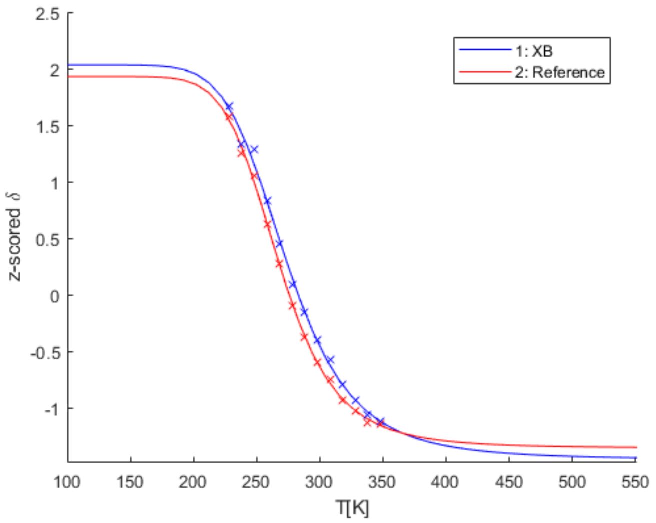

Figure S19: Overlap of extrapolated melting curves of halogen bonding **1** (blue) and the reference **2** (red). Data is obtained by function evaluation using fitted parameters found in *Table S27*. The y-axis represents the Z-score rescaled shift values necessary to average temperature shifts of amino acids with different shift range. Averaged shift values (δ_{av}) from observed amino acid Hα shift values are depicted as crosses.

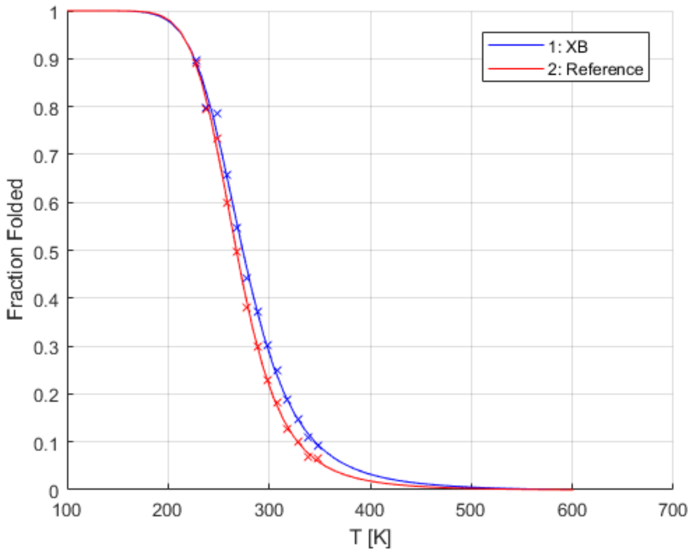

Figure S20: Overlap of normalized melting curves of halogen bonding **1** and the reference **2**. Curves are obtained from data extrapolation by function evaluation using fitted parameters found in *Table S27*.

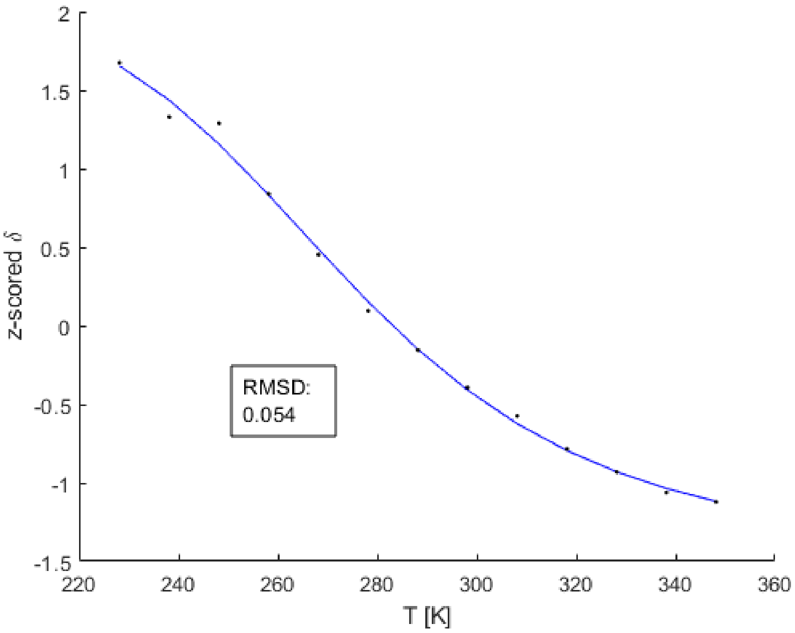

Figure S21: Result of curve-fitting to averaged shift data of **1**. Black dots represent the mean value of normalized Hα shifts for all amino acids. Assuming a two state behavior following the literature protocol of Munekata *et al.* experimental data points were fitted to a sigmoidal curve function depicted as blue line.

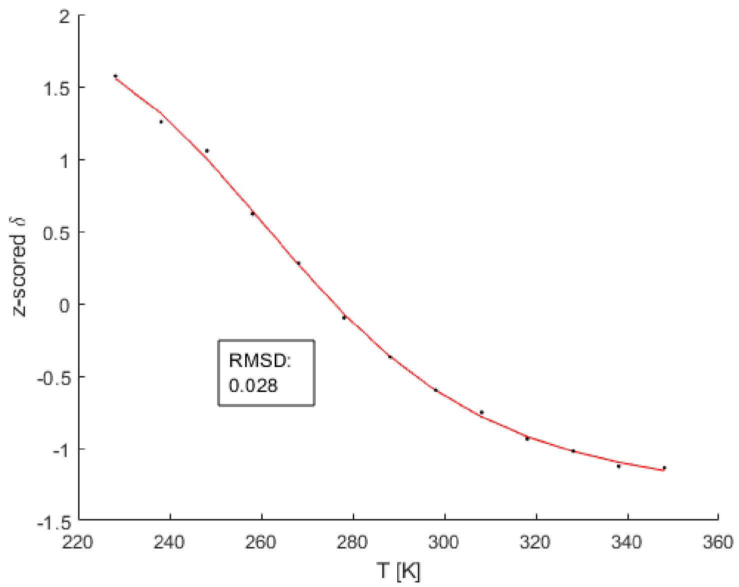

Figure S22: Result of curve-fitting to averaged shift data of **2**. Black dots represent the mean value of normalized Hα shifts for all amino acids. Assuming a two state behavior following the literature protocol of Munekata *et al.* experimental data points were fitted to a sigmoidal curve function depicted as red line.

As a relative descriptor of gain in stability comparing the halogen bonding compound **1** with the reference **2**, we deduced the ratio of folding constants as

$$K_F^{1/2} = \frac{k_F^1}{k_F^2} \qquad (S16)$$

where k_F^1 represents the folding constant of the halogen bonding **1** and k_F^2 the folding constant of the reference **2**. The folding constant k_F is commonly given as

$$k_F = \frac{[F]}{[U]} \qquad (S17)$$

where $[F]$ is the molar fraction of folded and $[U]$ is the molar fraction of unfolded peptide. Assuming a bimodal folding/unfolding behavior, both fractions must sum up to 1.

$$[F] + [U] = 1 \qquad (S18)$$

That assumption allows to denote

$$[F] = 1 - [U] \qquad (S19)$$

and

$$[U] = 1 - [F] \qquad (S20)$$

to be used in expressing K_F solely depending on the molar fractions of folded

$$[F] = \frac{k_F}{(k_F + 1)} \qquad (S21)$$

and unfolded peptide applying equation S17.

$$[U] = \frac{1}{(k_F + 1)} \qquad (S22)$$

Upon temperature change, the chemical shift observed by NMR (δ_{obs}) is a result of the change in ratio of molar fractions $[F]$ to $[U]$ within the limits of fully folded (δ_F) and unfolded (δ_U) shifts.

$$\delta_{obs} = \delta_F[F] + \delta_U[U] \qquad (S23)$$

Inserting deduced equation expressing $[F]$ and $[U]$ as function of K_F one obtains

$$\delta_{obs} = \delta_F\left\{\frac{k_F}{(k_F + 1)}\right\} + \delta_U\left\{\frac{1}{(k_F + 1)}\right\} \qquad (S24)$$

that now can be solved for K_F only depending on parameters observed by NMR spectroscopy.

$$k_F = \frac{(\delta_U - \delta_{obs})}{(\delta_{obs} - \delta_F)} \qquad (S25)$$

As **1** and **2** are highly similar in structure, and only differ in the presence of an additional non-covalent interaction site, the halogen bond in **1**, the observed melting curves are almost identical. To quantify the minor difference as change in thermodynamic stability we used the ratio ($K_F^{1/2}$) of their corresponding folding constants of **1** (k_F^1) to **2** (k_F^2).

$$K_F^{1/2} = \frac{k_F^1}{k_F^2} = \frac{(\delta_U^1 - \delta_{obs}^1)}{(\delta_{obs}^1 - \delta_F^1)} \Big/ \frac{(\delta_U^2 - \delta_{obs}^2)}{(\delta_{obs}^2 - \delta_F^2)} \qquad (S26)$$

The resulting value for $K_F^{1/2}$ can be calculated assuming a linear relationship of δ_{obs}^1 and δ_{obs}^2.[9] To visualize the dependency of the two folding constants we plotted $(\delta_{obs}^1 - \delta_F^1)(\delta_U^2 - \delta_{obs}^2)$ against $(\delta_U^1 - \delta_{obs}^1)(\delta_{obs}^2 - \delta_F^2)$. Values for folded and unfolded shift were obtained from the curve fitting procedure described above whereas values for δ_{obs} are extrapolated points to gain entire melting curves. As visible in Figure S23, plotting of the two curves against each other does not perfectly resemble a linear correlation. This is based in a difference in ΔH_m of both curves altering the ratio of δ_{obs}^1 to δ_{obs}^2. Assuming both curves would have identical ΔH_m all points would fall on the center of the symmetric 'surfboard'-shape plot. The slope of the line would not change as it represents the relative difference of T_m. Therefore, given a difference of ΔH_m between **1** and **2** within reasonable limits, linear regression analysis reveals the slope of the symmetric plot as the ratio $K_F^{1/2}$ of **1** to **2**.

$$K_F^{1/2}(\delta_{obs}^1 - \delta_F^1)(\delta_U^2 - \delta_{obs}^2) = (\delta_U^1 - \delta_{obs}^1)(\delta_{obs}^2 - \delta_F^2) \qquad (S27)$$

We found the slope $K_F^{1/2}$ to be 1.3018 indicating a shift of the melting curve for **1** to higher temperatures relative to **2**. Hence, **1** appears to have a 30% higher thermodynamic stability than **2** that must arise from the formation of a cross-strand halogen bond.

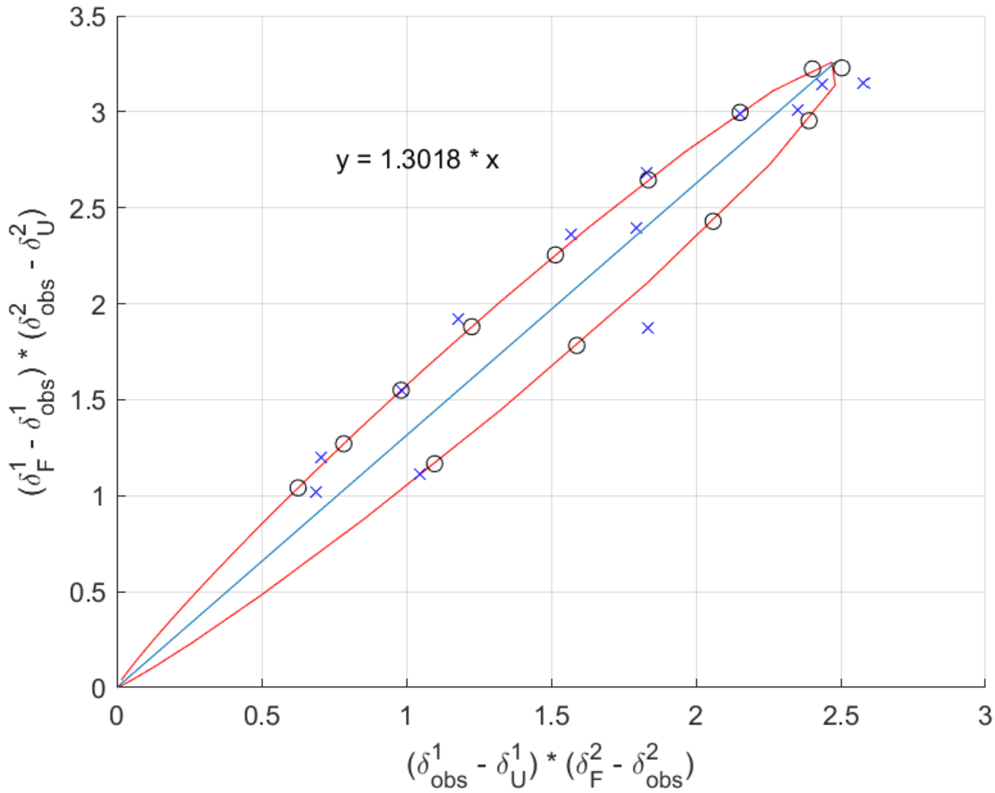

Figure S23: Relative folding ratio of **1** to **2**. All depicted data points were plotted applying equation S27 using different values for δ_{obs}. Red line represents the calculated values obtained from curve fitting and temperature extension. Black circles represent fitted melting curve data points within the experimental temperature range. Blue crosses depict the δ_{av} values resulting from rescaling and averaging experimentally determined shift changes.

7 DFT calculations

To evaluate the halogen bond interaction strength, we performed DFT calculations on the complex of iodobenzene and diethylether, and on that of *meta*-ethyl-iodobenzene and methoxypropane that resembles the truncated side chains of peptide **1** that form the halogen bond in focus. DFT calculations were run using the M06-2X functional[15] with the Grimme dispersion correction[16] in combination with the def2-TZVP(D) basis set, in *vacuo*.[17] Results are summarized in Tables S26 and S27.

Table S27: Interaction energies of iodobenzene and diethylether calculated using M062X/def2-TZVP(D). Values are given in Hartree atomic units unless stated otherwise. Resulting angle of C–I···O was 177.6° with a 3.01 Å I···O interatomic distance i.e. 94% of the sum or vdW radii. Hydrogen atoms are omitted for clarity.

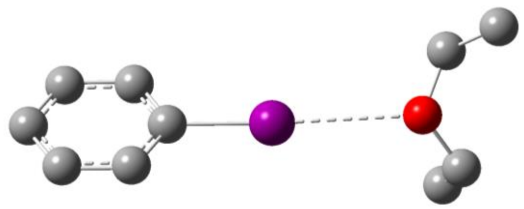

	C_6H_5-I	C_2H_5-O-C_2H_5	Complex	[Ht]	[kcal mol^{-1}]	[kJ mol^{-1}]
ΔE	-233.631	-529.257	-762.894	-0.006	-3.858	-16.142
ΔH	-233.485	-529.159	-762.649	-0.004	-2.628	-10.996
ΔG	-233.523	-529.197	-762.713	0.008	4.869	20.374
ΔG^{Grimme}	-233.52273	-529.197256	-762.707547	0.012	7.806	32.659

Table S28: Interaction energies of *meta*-ethyl-iodobenzene and methoxypropane calculated using M062X/def2-TZVP(D). Values are given in Hartree atomic units unless stated otherwise. Resulting angle of C–I···O was 173.0° with a 3.05 Å I···O interatomic distance i.e. 95% of the sum or vdW radii. Hydrogen atoms are omitted for clarity.

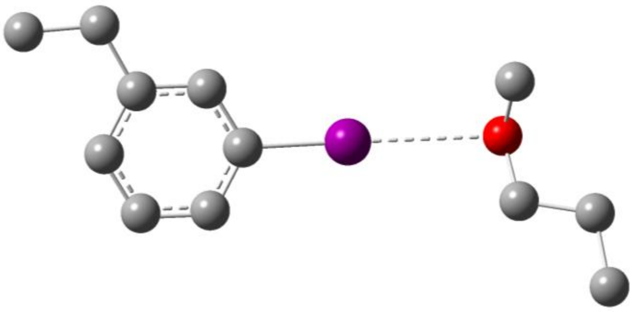

	C_8H_9-I	CH_3-O-C_3H_7	Complex	[Ht]	[kcal/mol]	[kJ/mol]
ΔE	-233.628	-607.874	-841.508	-0.006	-3.817	-15.968
ΔH	-233.482	-607.717	-841.203	-0.004	-2.556	-10.694
ΔG	-233.520	-607.763	-841.275	0.008	5.239	21.920
ΔG^{Grimme}	-233.519955	-607.761809	-841.268441	0.013	8.360	34.980

The similar ΔH and ΔG of the two systems suggest that neither steric nor electronic factors have a significant influence on the I to O halogen bond interaction of peptide **1**. The > ΔG confirms that this weak halogen bond would not be experimentally detectable in dilute solution at room temperature in an *inter*molecular system. Model system **1** was designed to make the experimental characterization of such a weak non covalent bond suitable, incorporating it into an *intra*molecular model system.

8 NMR spectra

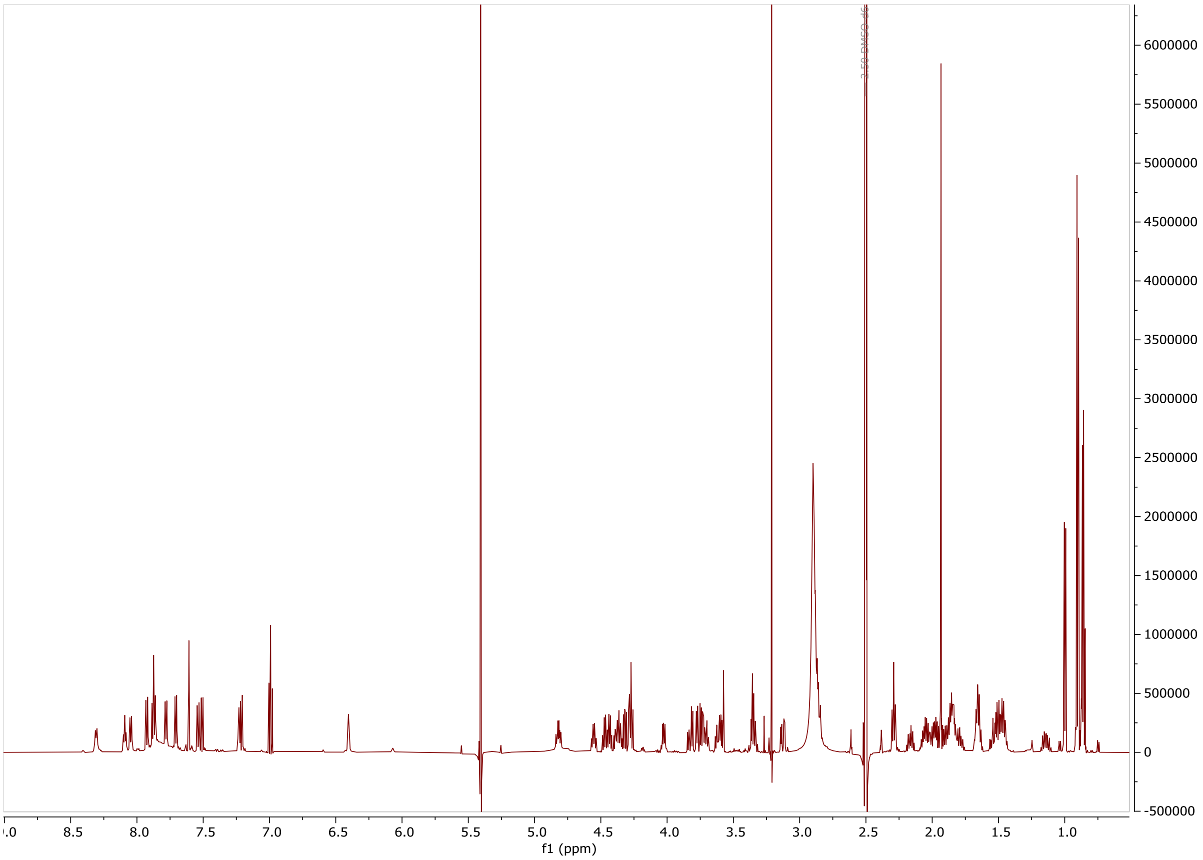

Figure 24: ^{1}H-NMR of **1** in CD_2Cl_2:DMSO-d_6 4:1 (600 MHz).

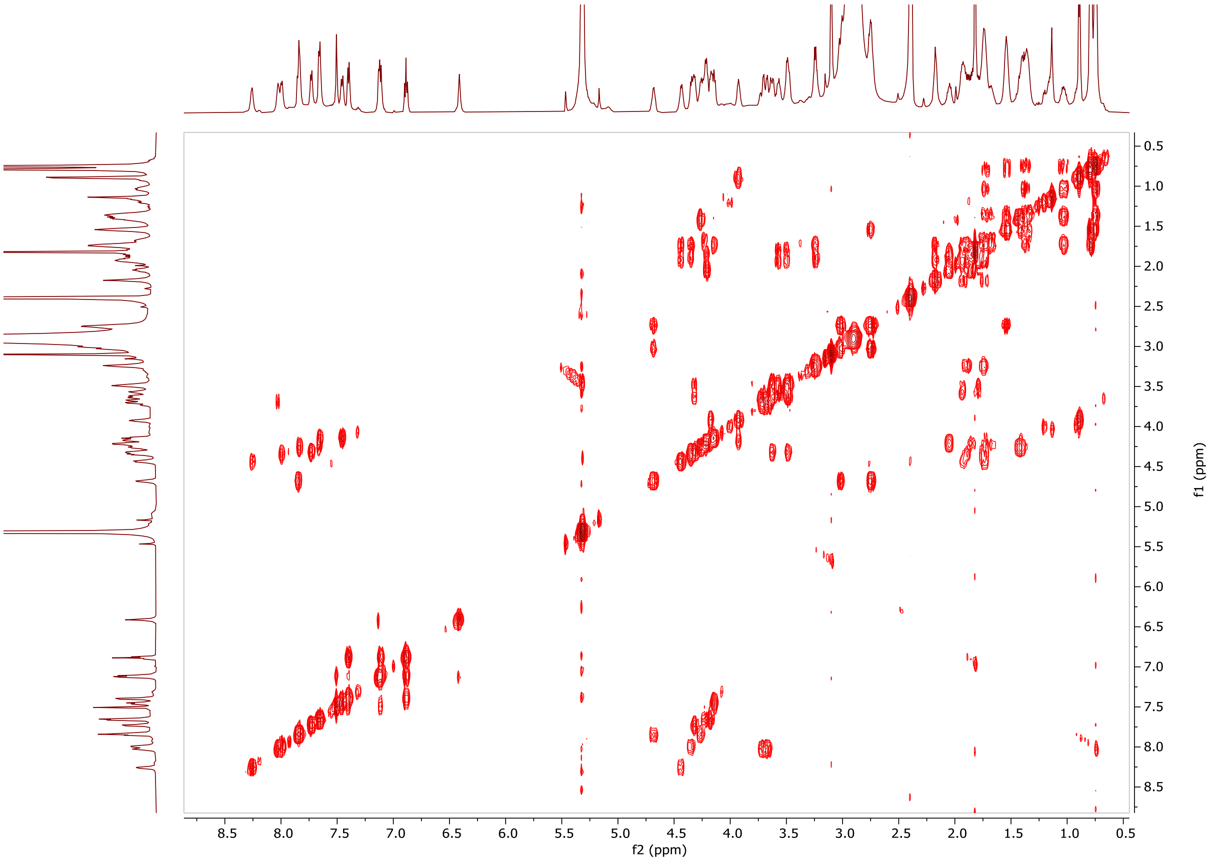

Figure 25: COSY spectrum of **1** in CD_2Cl_2:DMSO-d$_6$ 4:1 (600 MHz).

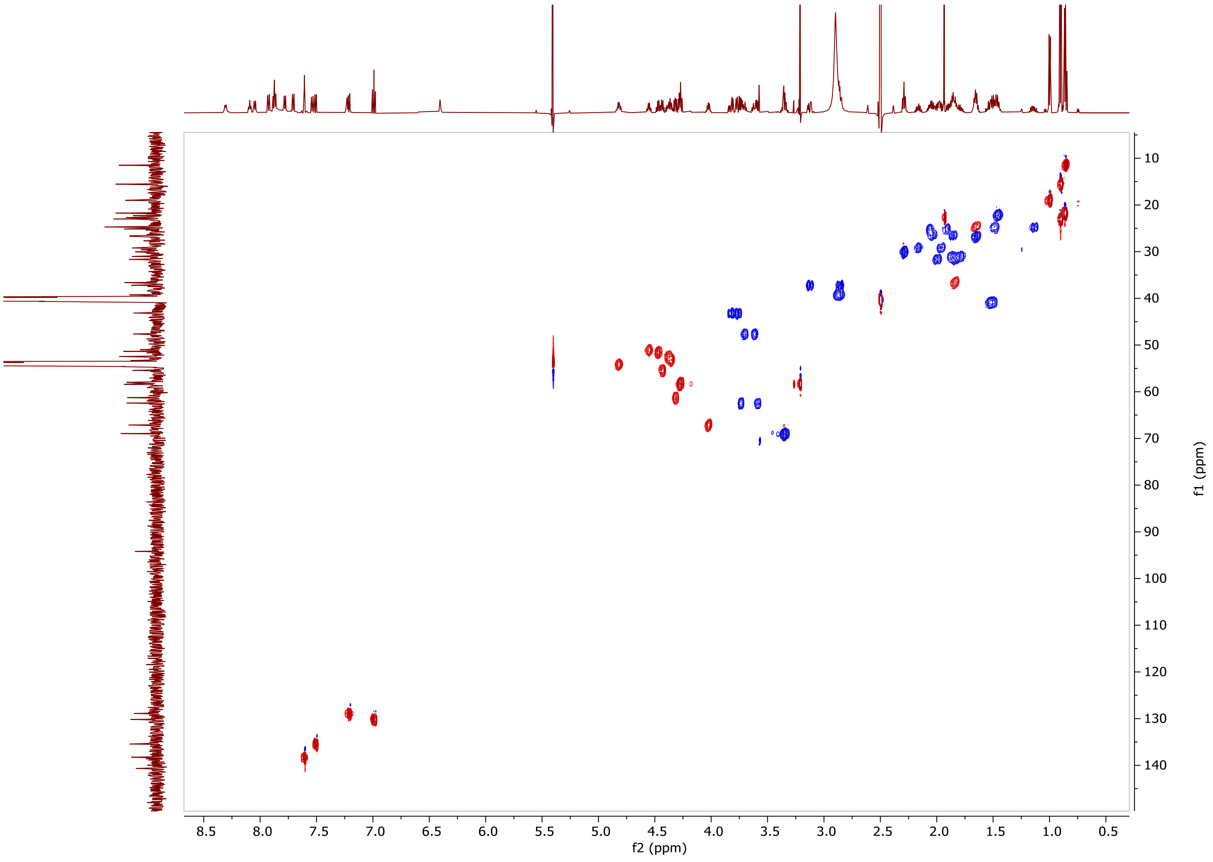

*Figure 26: $^{1}H^{13}C$ -HSQC spectrum of **1*** in CD_2Cl_2:DMSO-d$_6$ 4:1 (600 MHz).

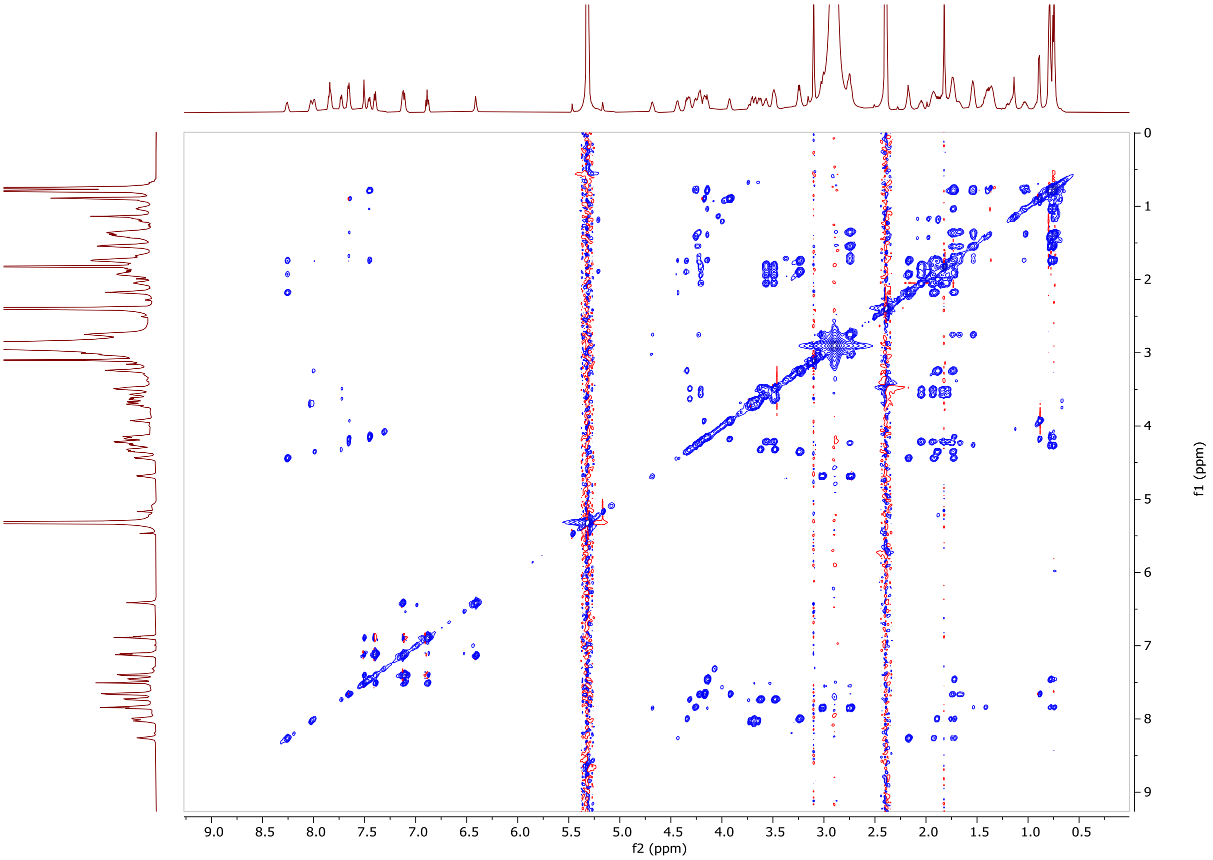

Figure 27: TOCSY spectrum of **1** in CD_2Cl_2:DMSO-d_6 4:1 (600 MHz).

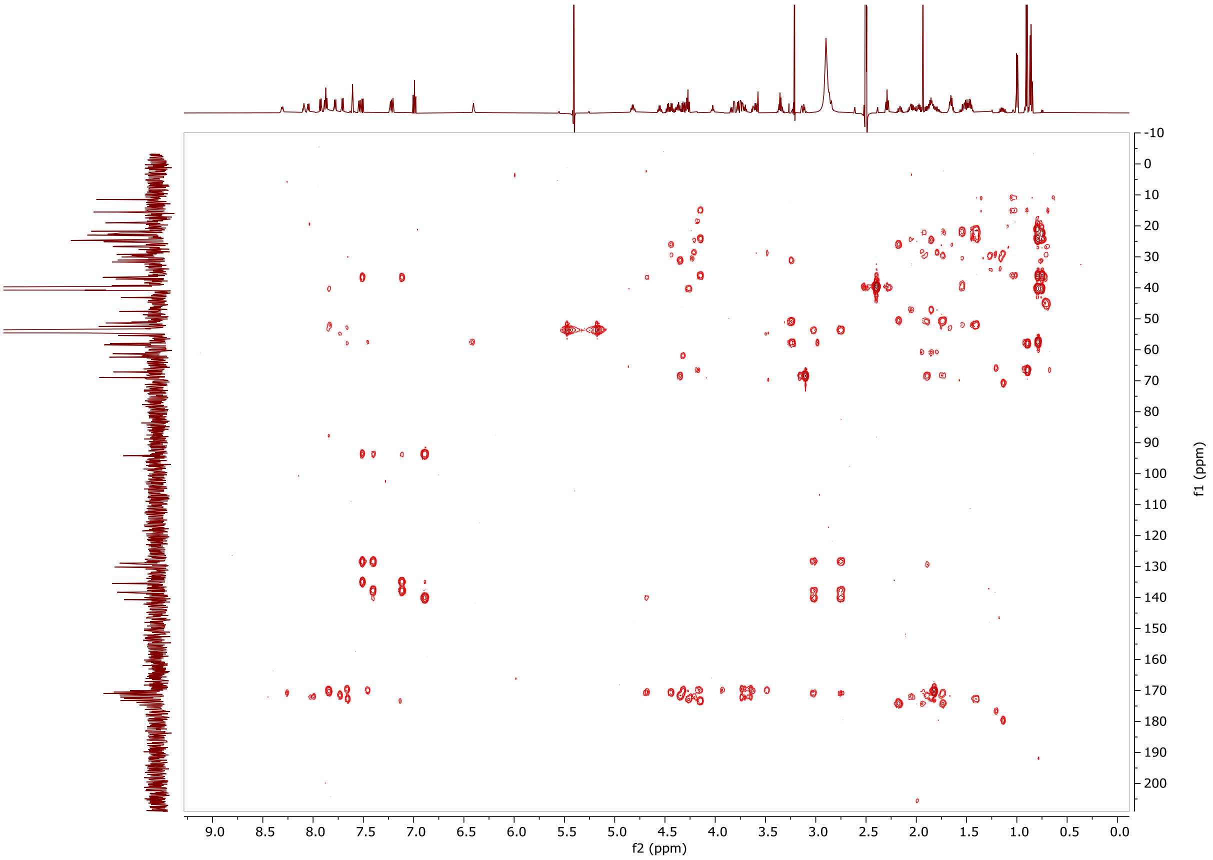

Figure 28: $^{1}H^{13}C$ -HMBC spectrum of **1** in CD_2Cl_2:DMSO-d_6 4:1 (600 MHz).

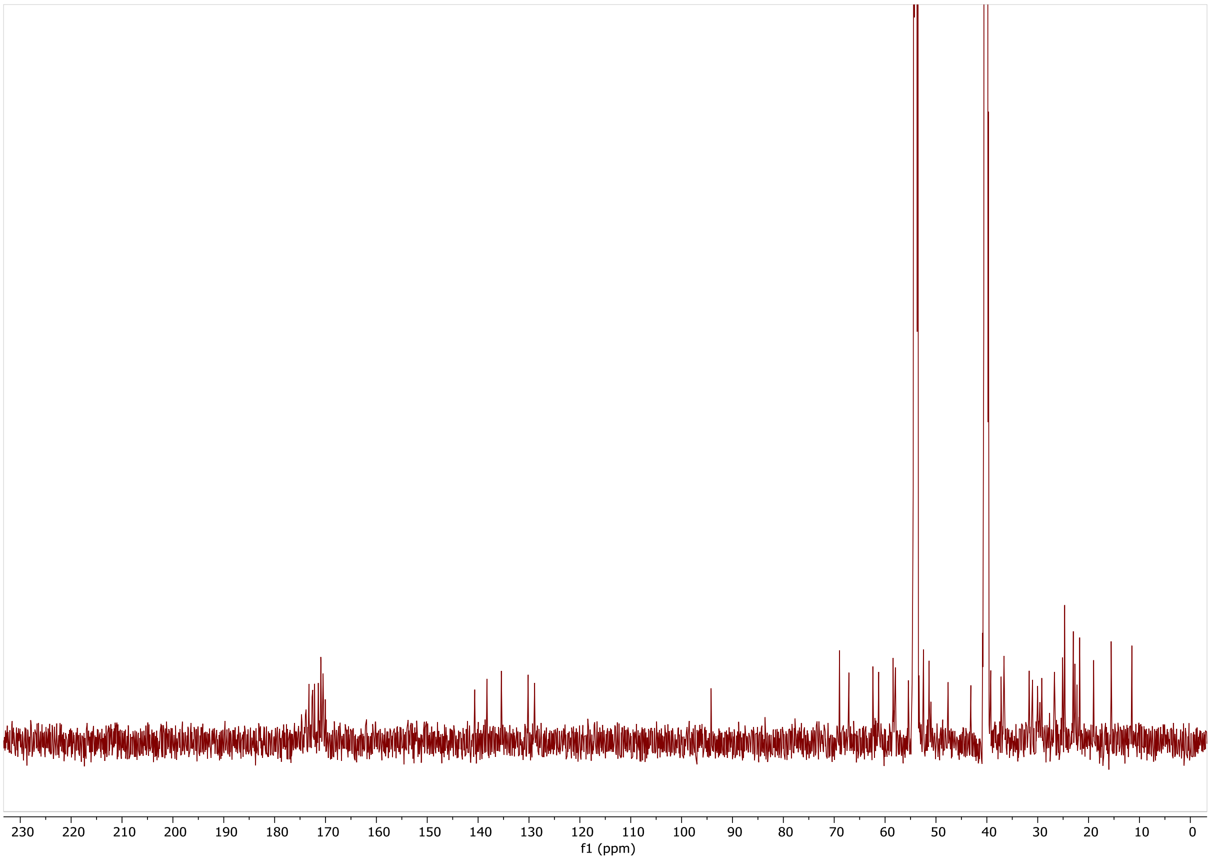

Figure 29: ^{13}C-NMR of **1** in CD_2Cl_2:DMSO-d_6 4:1 (150 MHz).

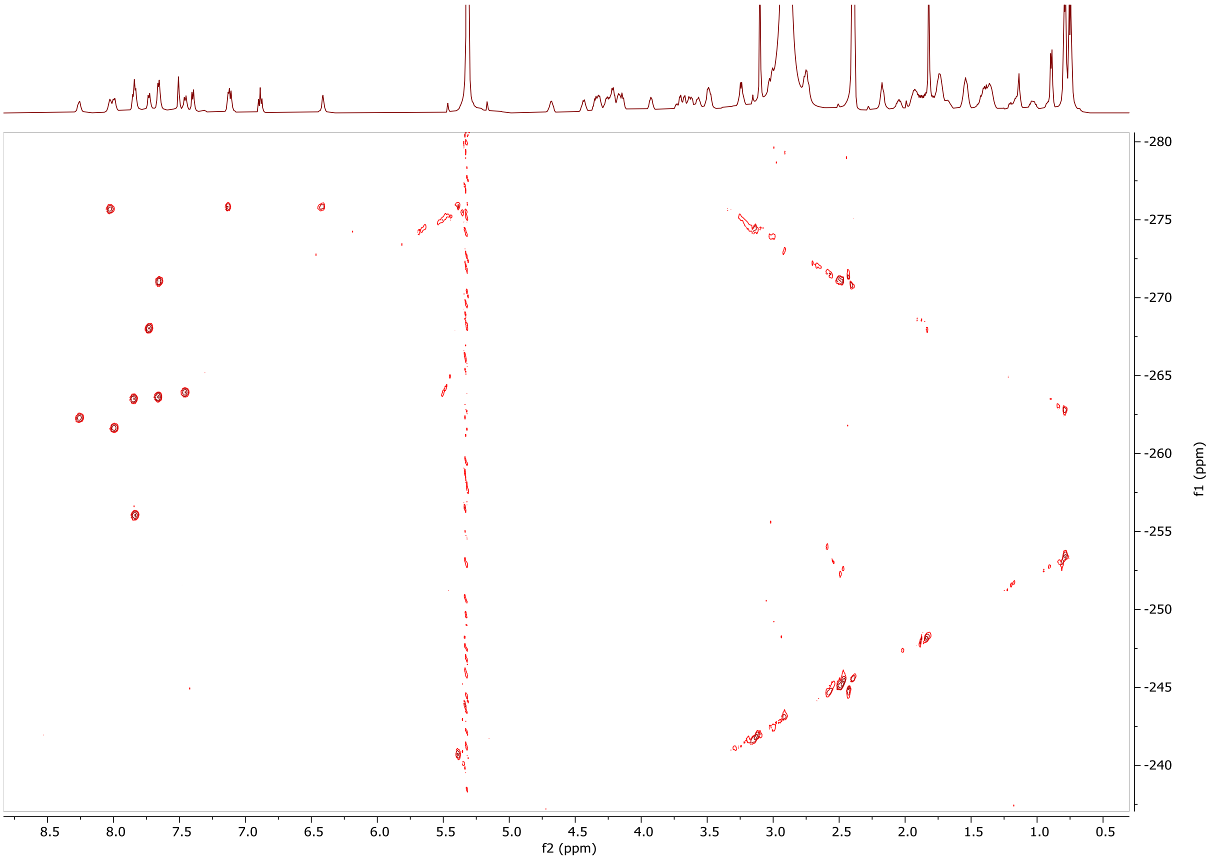

Figure 30: ^{1}H^{15}N -HSQC spectrum of **1** in CD_2Cl_2:DMSO-d_6 4:1 (600 MHz).

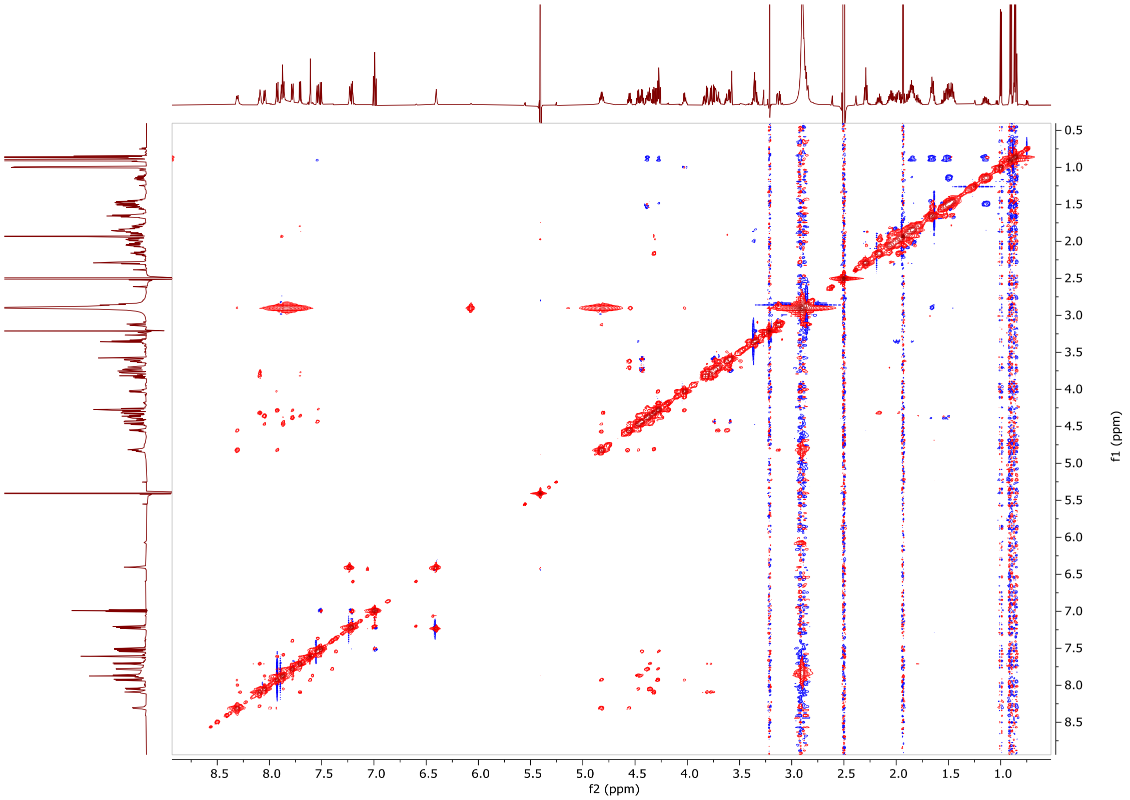

Figure 31: NOESY spectrum of **1** recorded with a 700 ms mixing time in CD_2Cl_2:DMSO-d_6 4:1 (600 MHz). Note: the positive (blue) NOE cross peaks of some side chain protons is due to their high mobility as compared to the overall tumbling of the entire molecule, which makes the double quantum ω_2 relaxation pathway to dominate over the zero quantum ω_0 pathway.

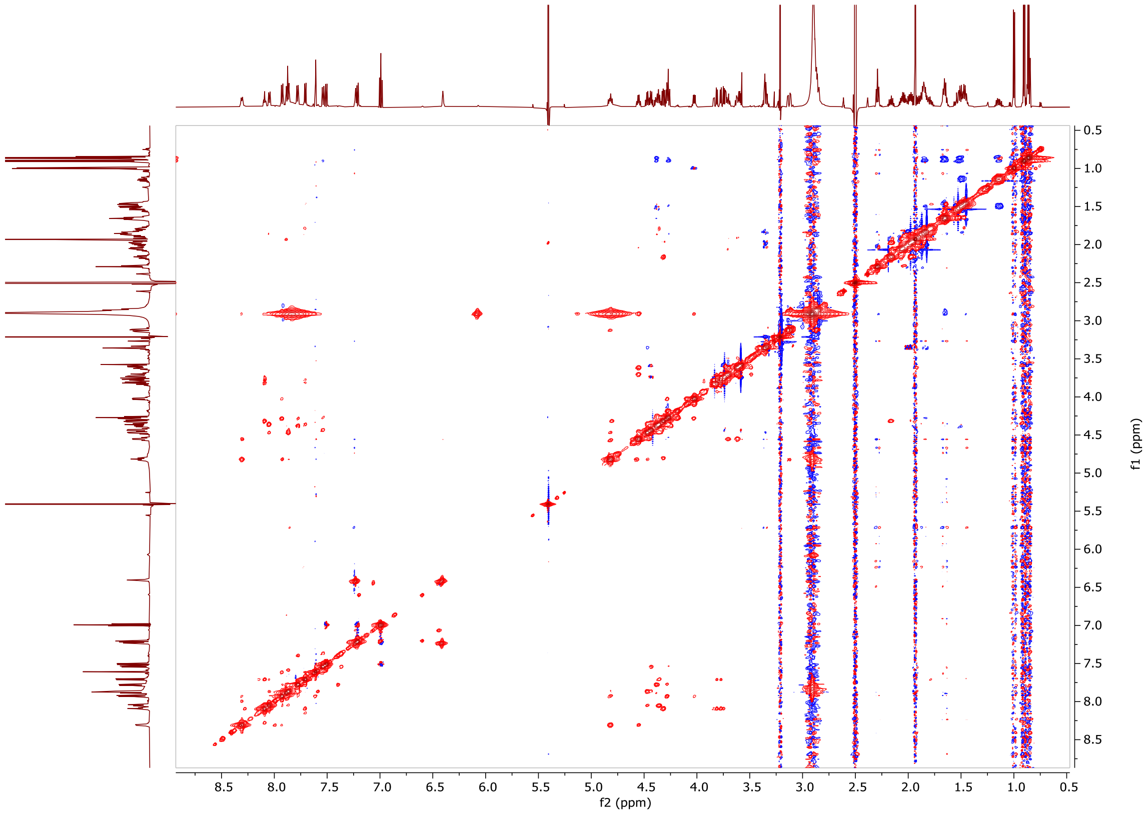

Figure 32: NOESY spectrum of **1** recorded with a 600 ms mixing time in CD_2Cl_2:DMSO-d_6 4:1 (600 MHz). Note: the positive (blue) NOE cross peaks of some side chain protons is due to their high mobility as compared to the overall tumbling of the entire molecule, which makes the double quantum ω_2 relaxation pathway to dominate over the zero quantum ω_0 pathway.

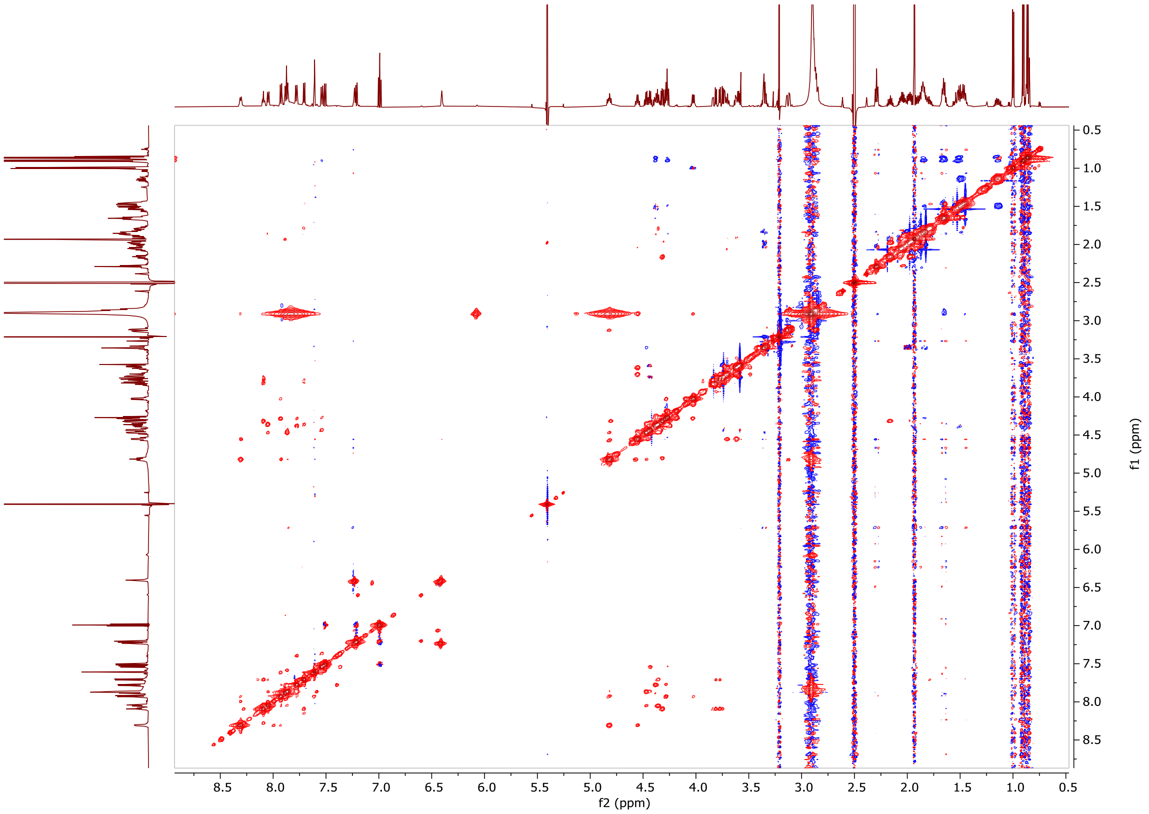

*Figure 33: NOESY spectrum of **1** recorded with a 500 ms mixing time* in CD_2Cl_2:DMSO-d_6 4:1 (600 MHz). Note: the positive (blue) NOE cross peaks of some side chain protons is due to their high mobility as compared to the overall tumbling of the entire molecule, which makes the double quantum ω_2 relaxation pathway to dominate over the zero quantum ω_0 pathway.

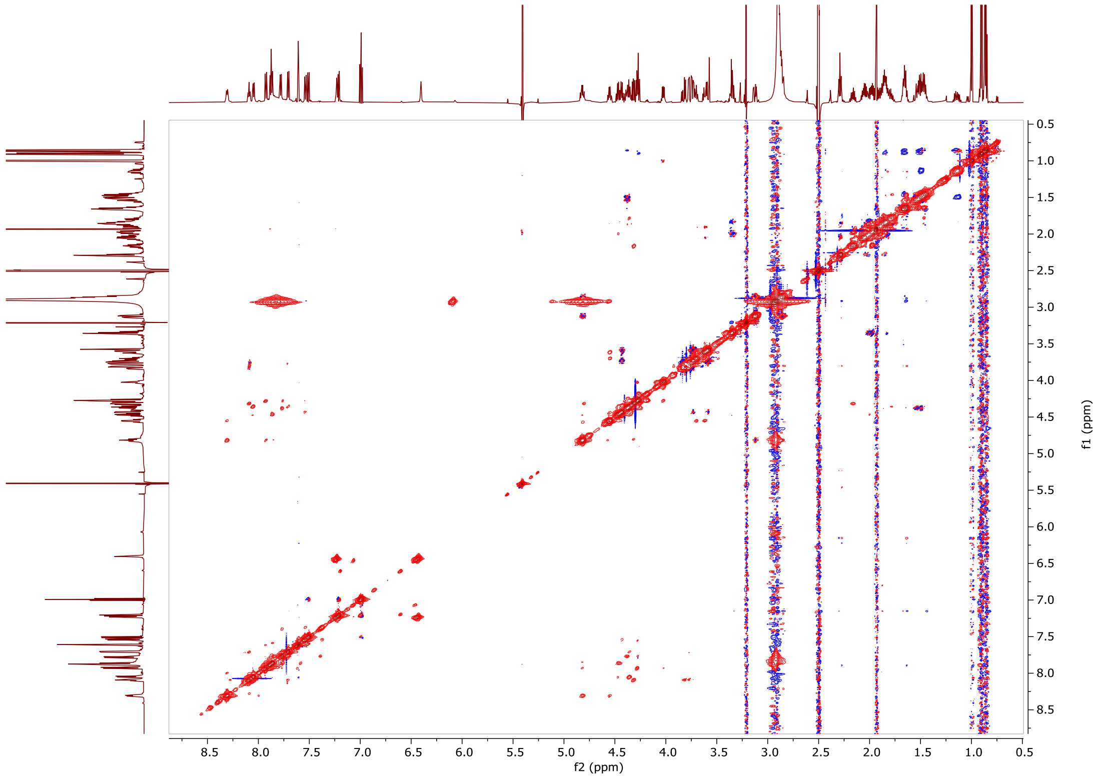

*Figure 34: NOESY spectrum of **1** recorded with a 400 ms mixing time* in CD_2Cl_2:DMSO-d_6 4:1 (600 MHz). Note: the positive (blue) NOE cross peaks of some side chain protons is due to their high mobility as compared to the overall tumbling of the entire molecule, which makes the double quantum ω_2 relaxation pathway to dominate over the zero quantum ω_0 pathway.

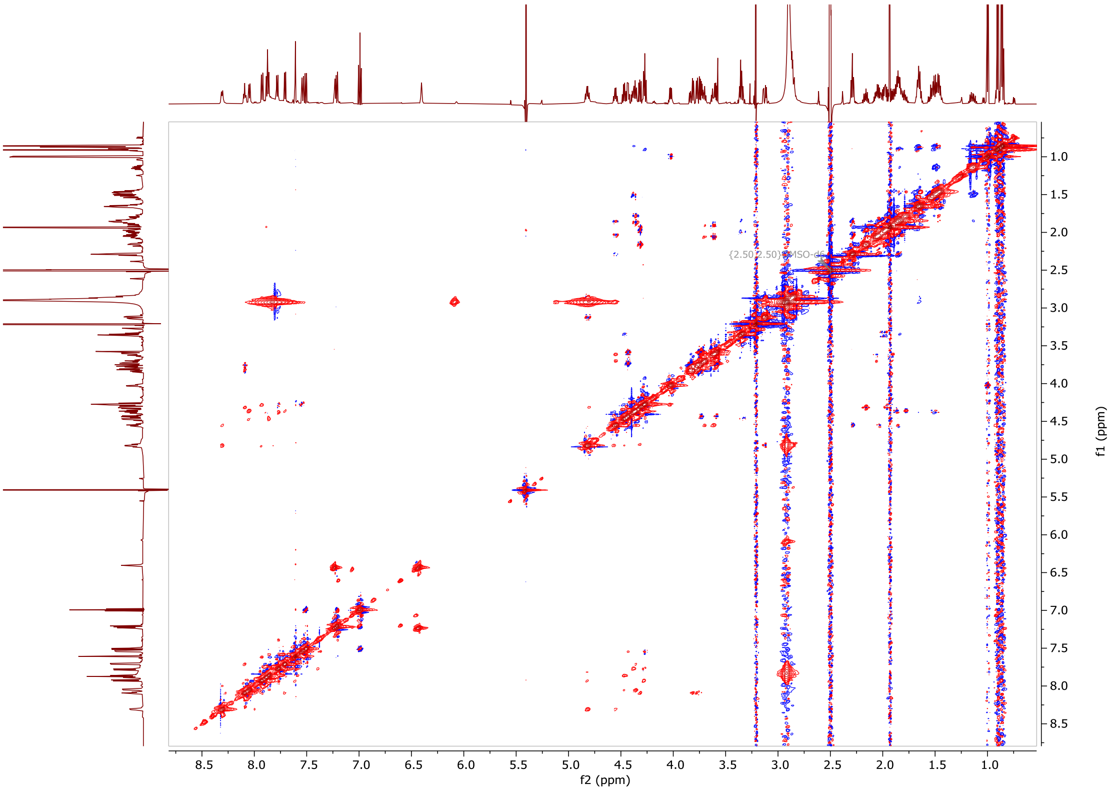

Figure 35: NOESY spectrum of **1** recorded with a 300ms mixing time in CD_2Cl_2:DMSO-d_6 4:1 (600 MHz). Note: the positive (blue) NOE cross peaks of some side chain protons is due to their high mobility as compared to the overall tumbling of the entire molecule, which makes the double quantum ω_2 relaxation pathway to dominate over the zero quantum ω_0 pathway.

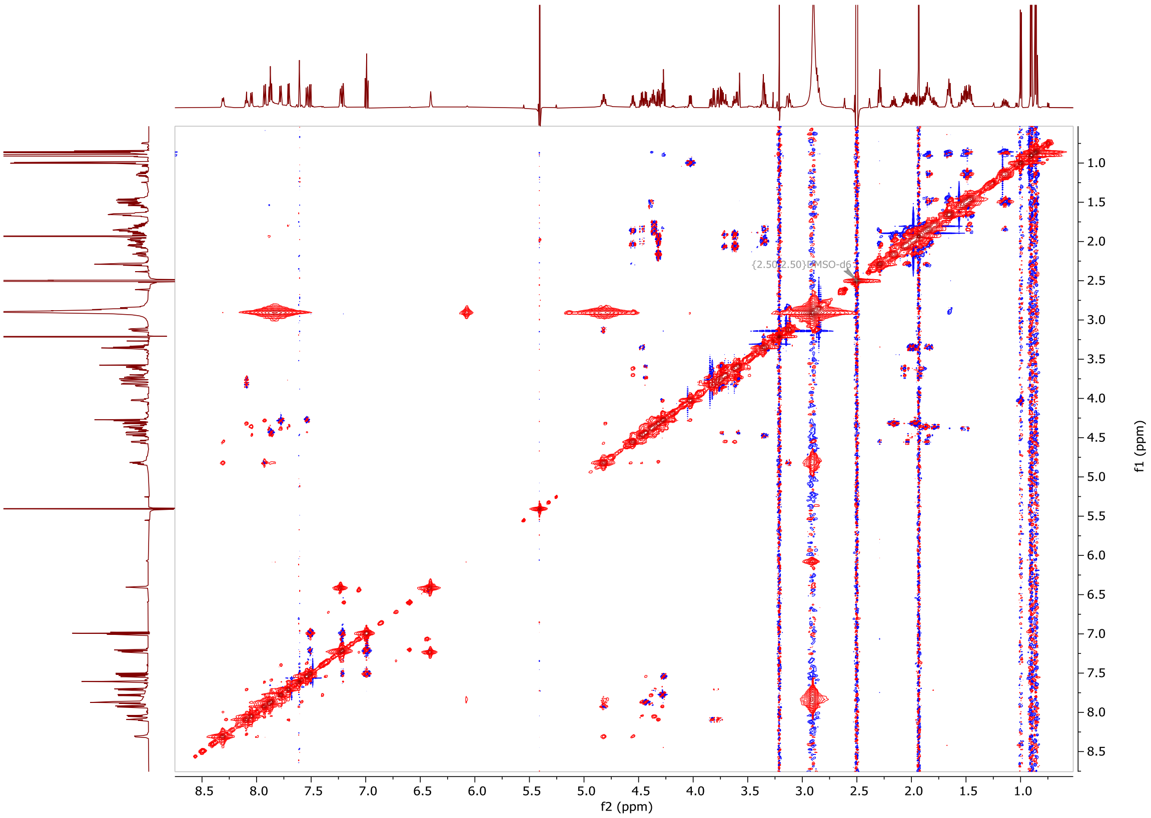

Figure 36: NOESY spectrum of **1** recorded with a 200 ms mixing time in CD_2Cl_2:DMSO-d_6 4:1 (600 MHz). Note: the positive (blue) NOE cross peaks of some side chain protons is due to their high mobility as compared to the overall tumbling of the entire molecule, which makes the double quantum ω_2 relaxation pathway to dominate over the zero quantum ω_0 pathway.

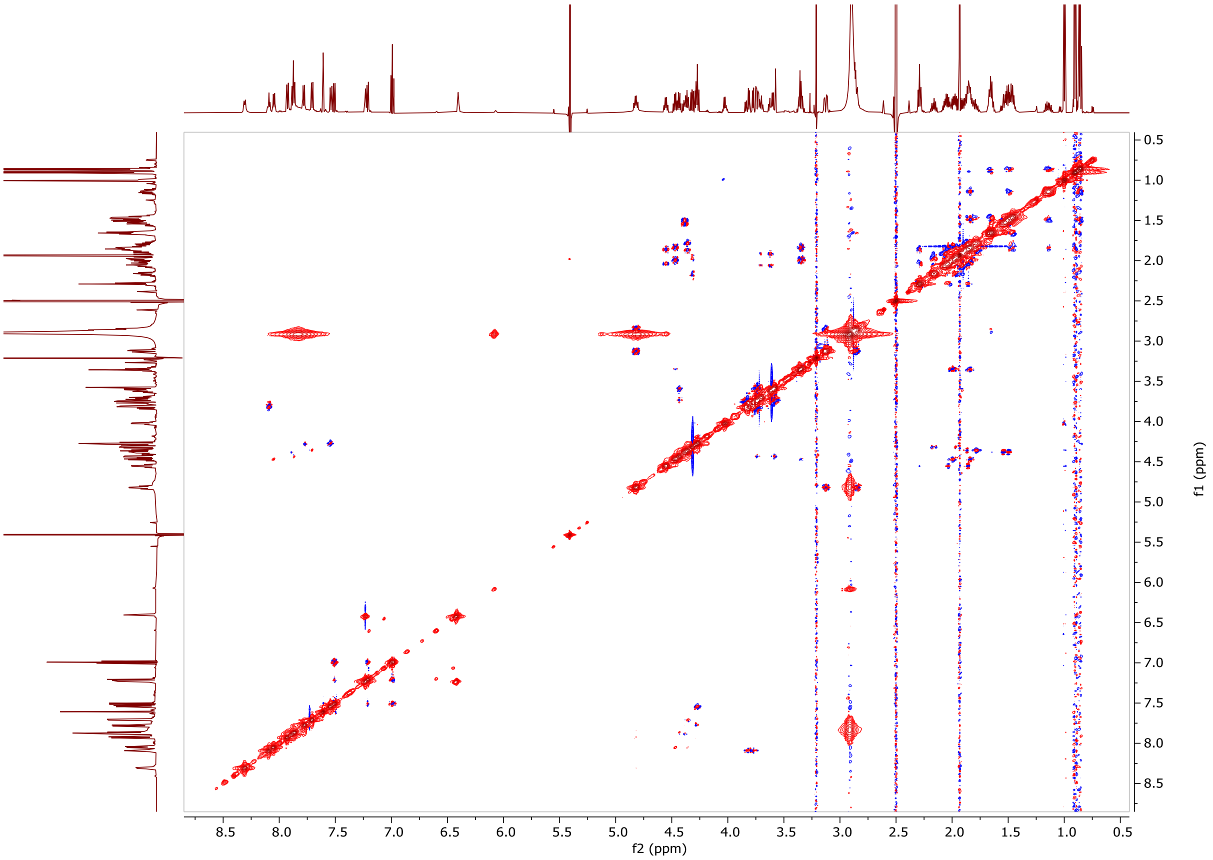

Figure 37: NOESY spectrum of **1** recorded with a 100 ms mixing time in CD_2Cl_2:DMSO-d_6 4:1 (600 MHz). Note: the positive (blue) NOE cross peaks of some side chain protons is due to their high mobility as compared to the overall tumbling of the entire molecule, which makes the double quantum ω_2 relaxation pathway to dominate over the zero quantum ω_0 pathway.

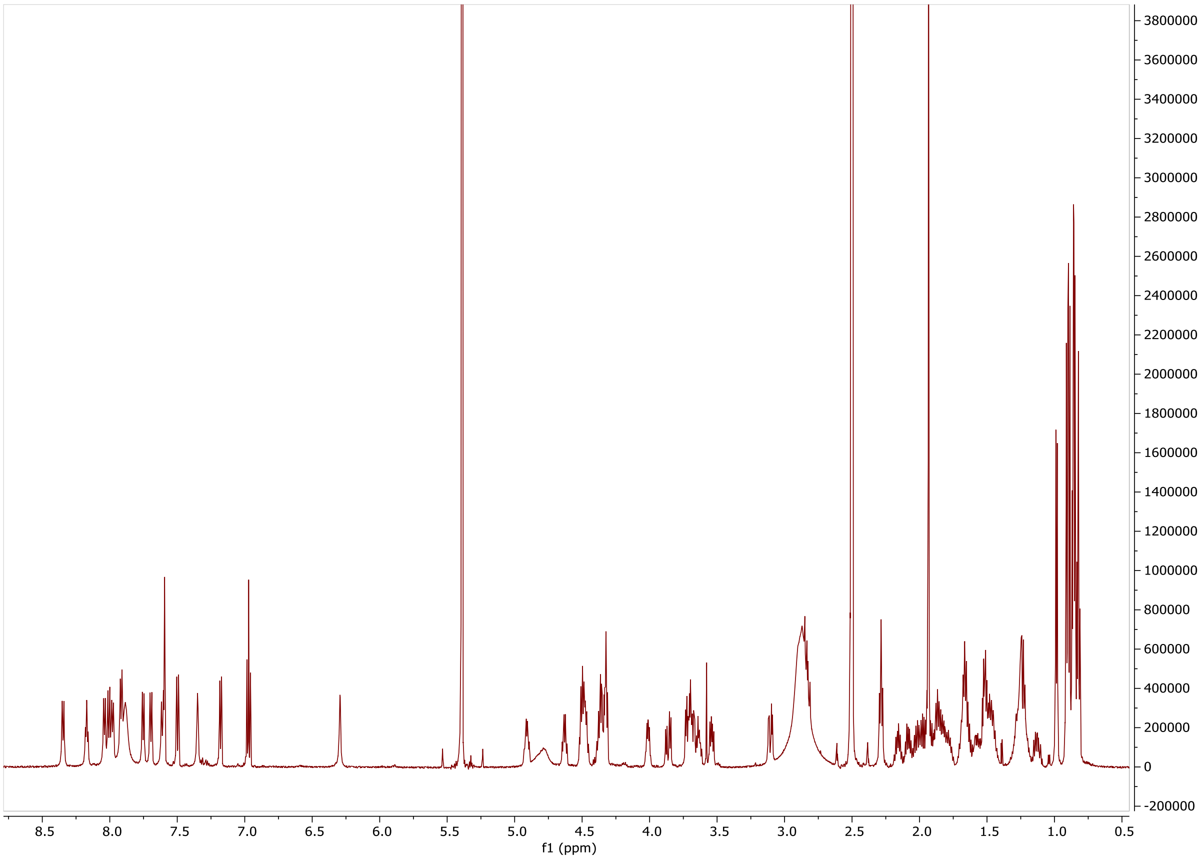

Figure 38: ^{1}H-NMR spectrum of **2** in CD_2Cl_2:DMSO-d_6 4:1 (600 MHz).

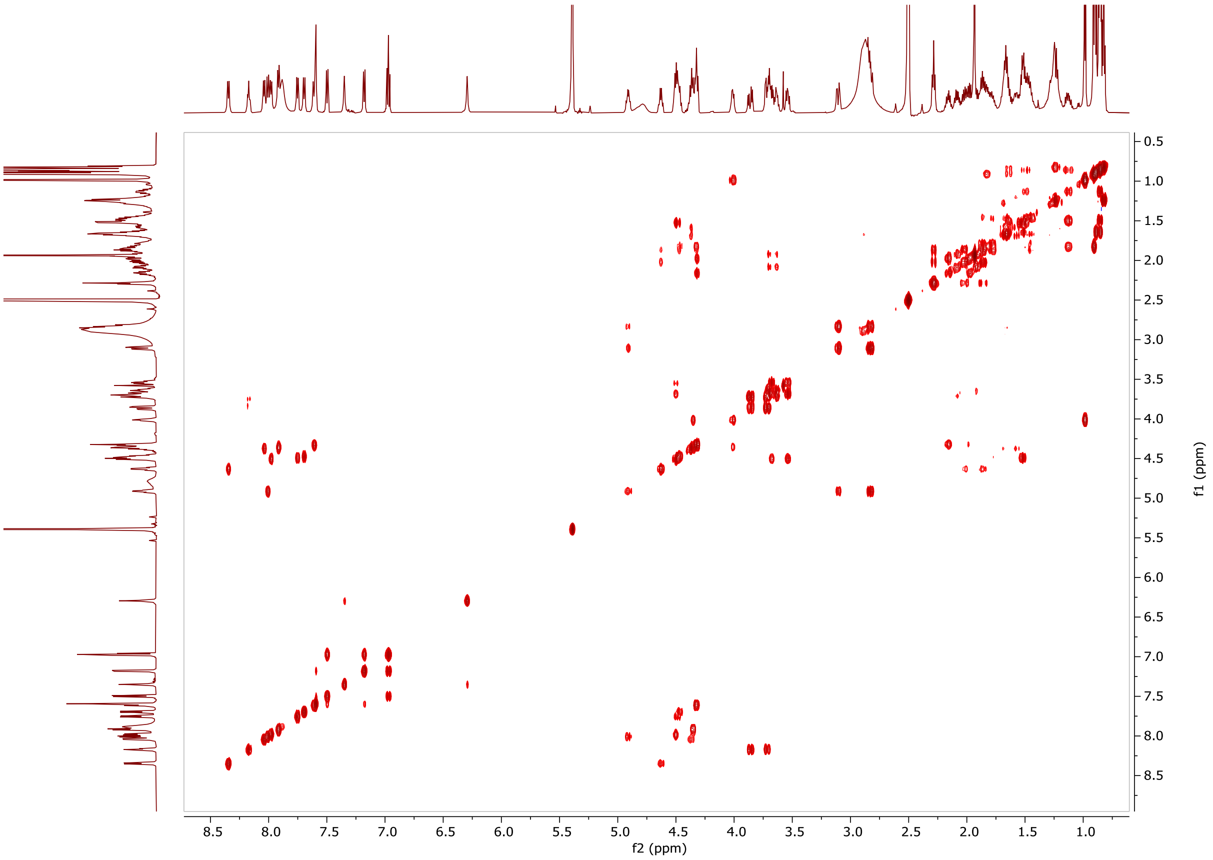

Figure 39: COSY spectrum of **2** in CD_2Cl_2:DMSO-d_6 4:1 (600 MHz).

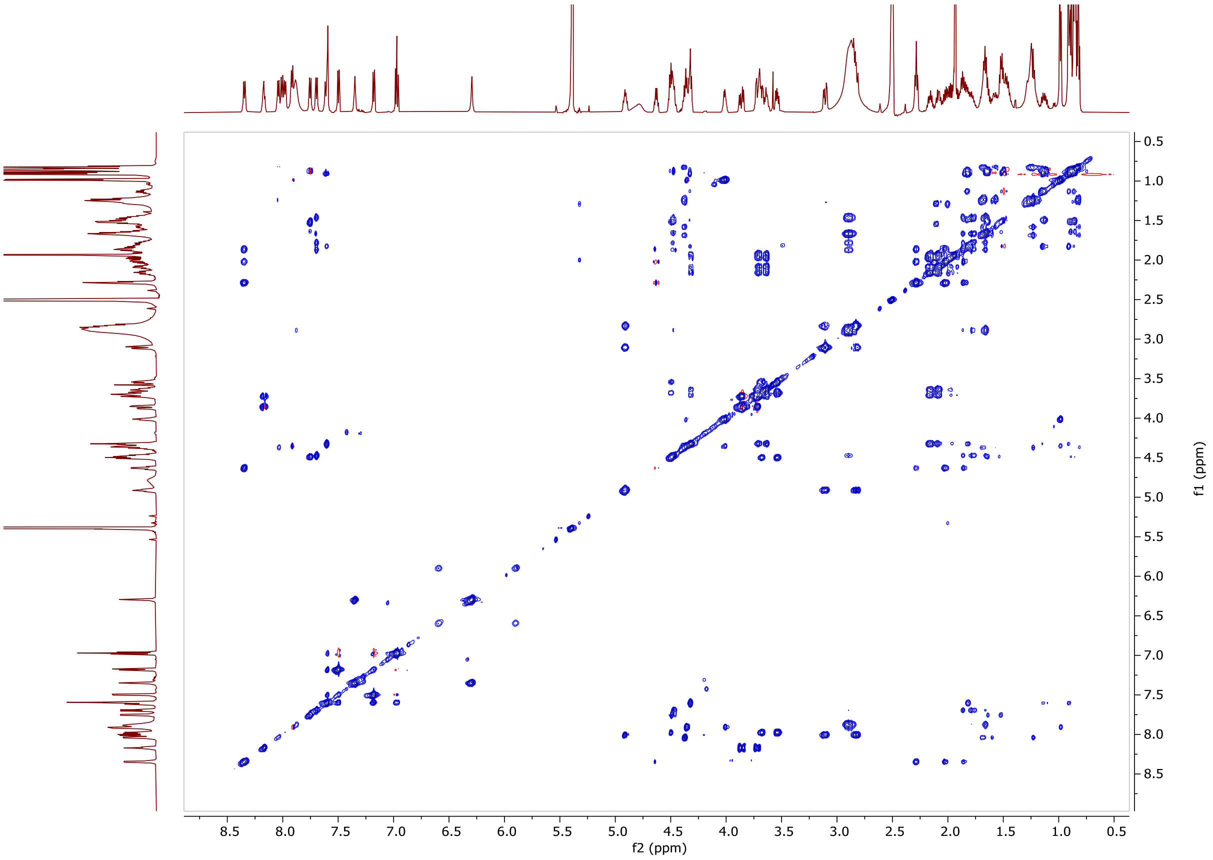

Figure 40: TOCSY spectrum of **2** in CD_2Cl_2:DMSO-d_6 4:1 (600 MHz).

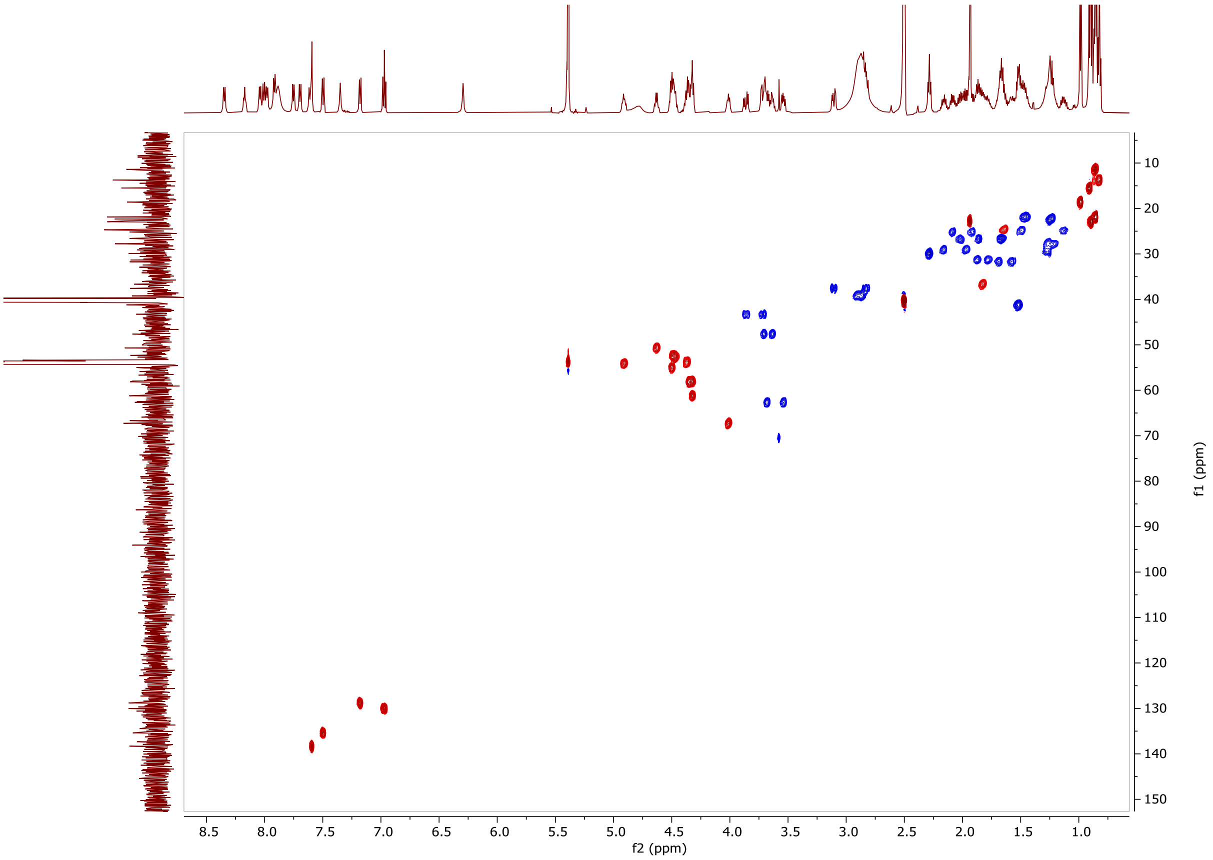

Figure 41: $^{13}C^{1}H$-HSQC spectrum of **2** in CD_2Cl_2:DMSO-d_6 4:1 (600 MHz).

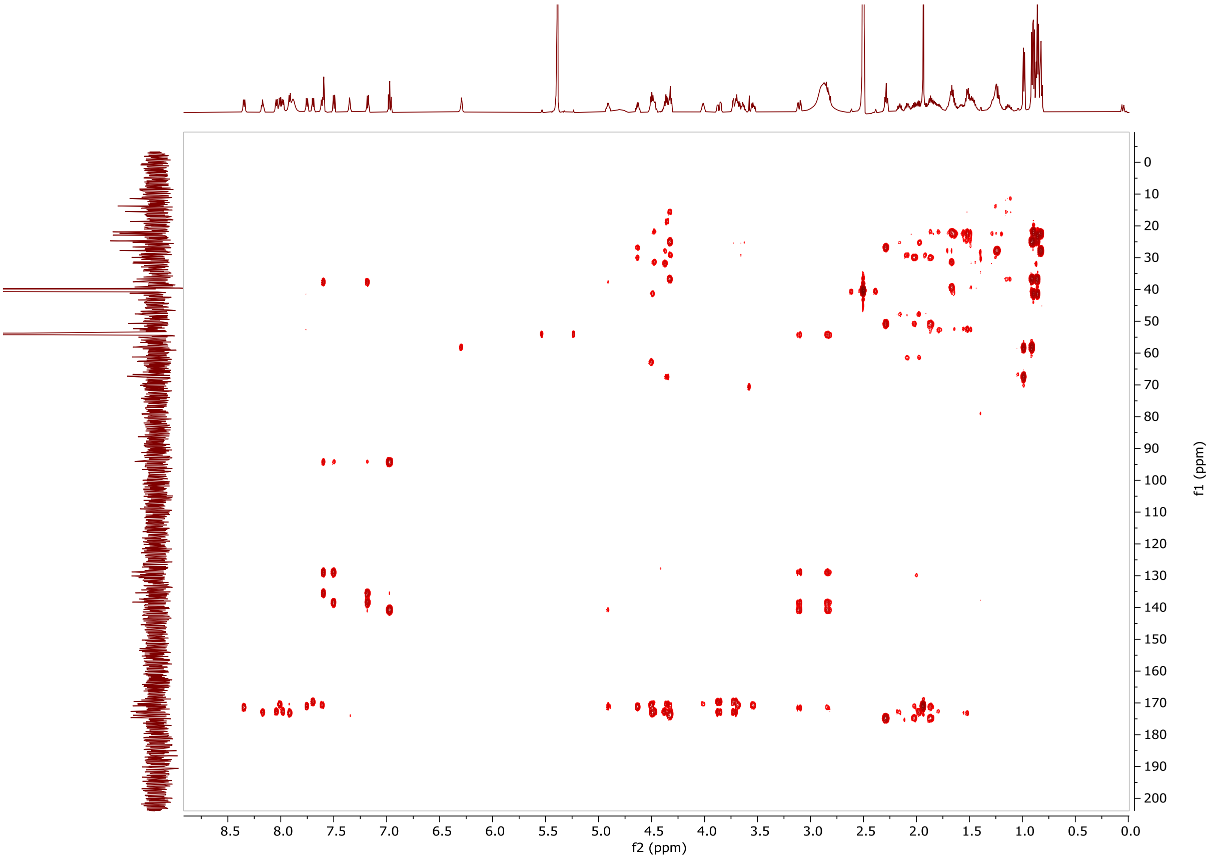

Figure 42: $^{1}H^{13}C$ -HMBC spectrum of **2** in CD_2Cl_2:DMSO-d_6 4:1 (600 MHz).

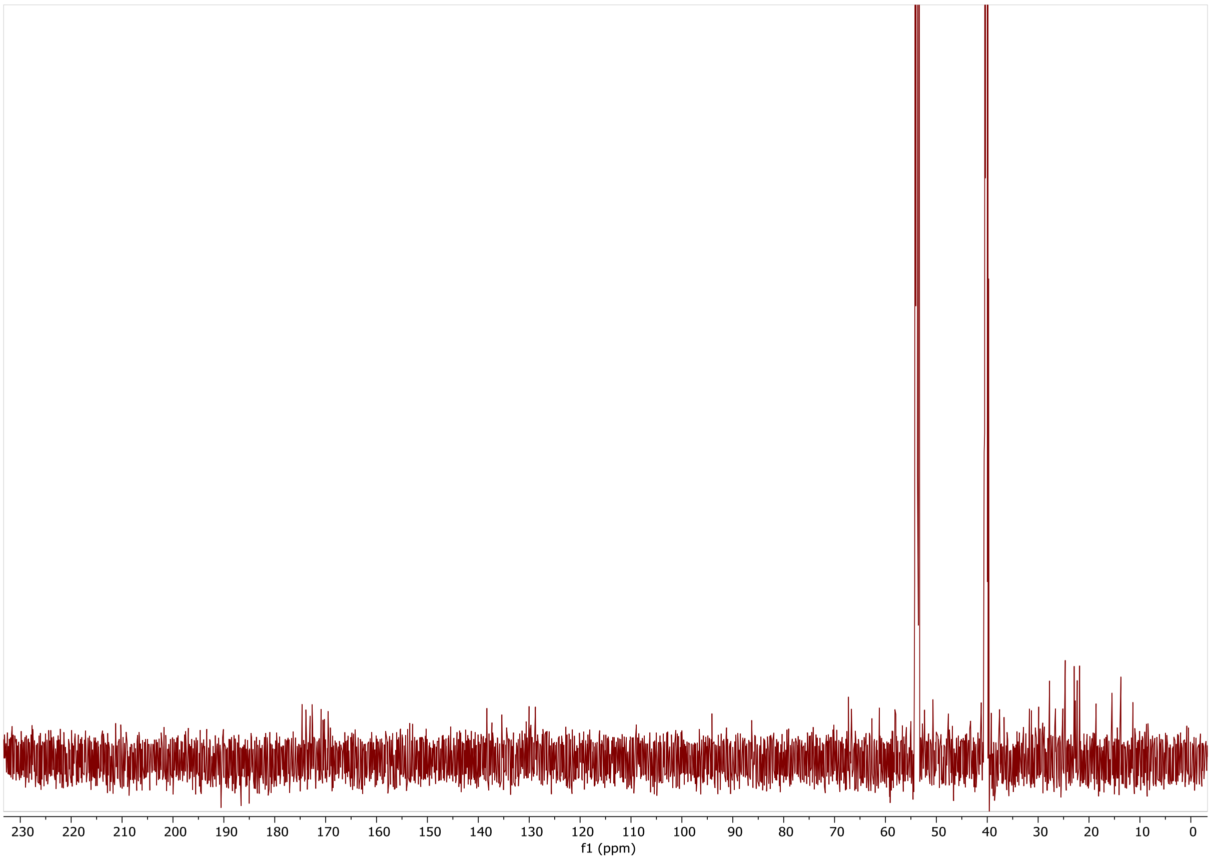

Figure 43: ^{13}C-NMR spectrum of **2** in CD_2Cl_2:DMSO-d_6 4:1 (150 MHz).

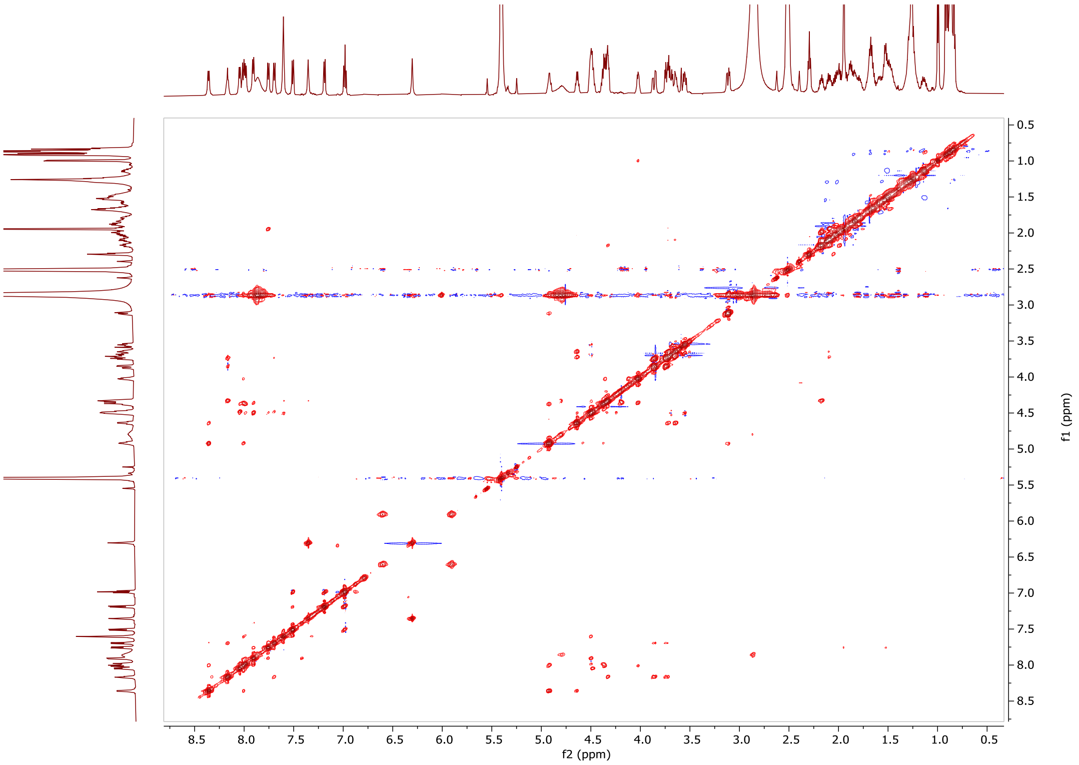

Figure 44: NOESY spectrum of **2** recorded with a 700 ms mixing time in CD_2Cl_2:DMSO-d_6 4:1 (600 MHz). Note: the positive (blue) NOE cross peaks of some side chain protons is due to their high mobility as compared to the overall tumbling of the entire molecule, which makes the double quantum ω_2 relaxation pathway to dominate over the zero quantum ω_0 pathway.

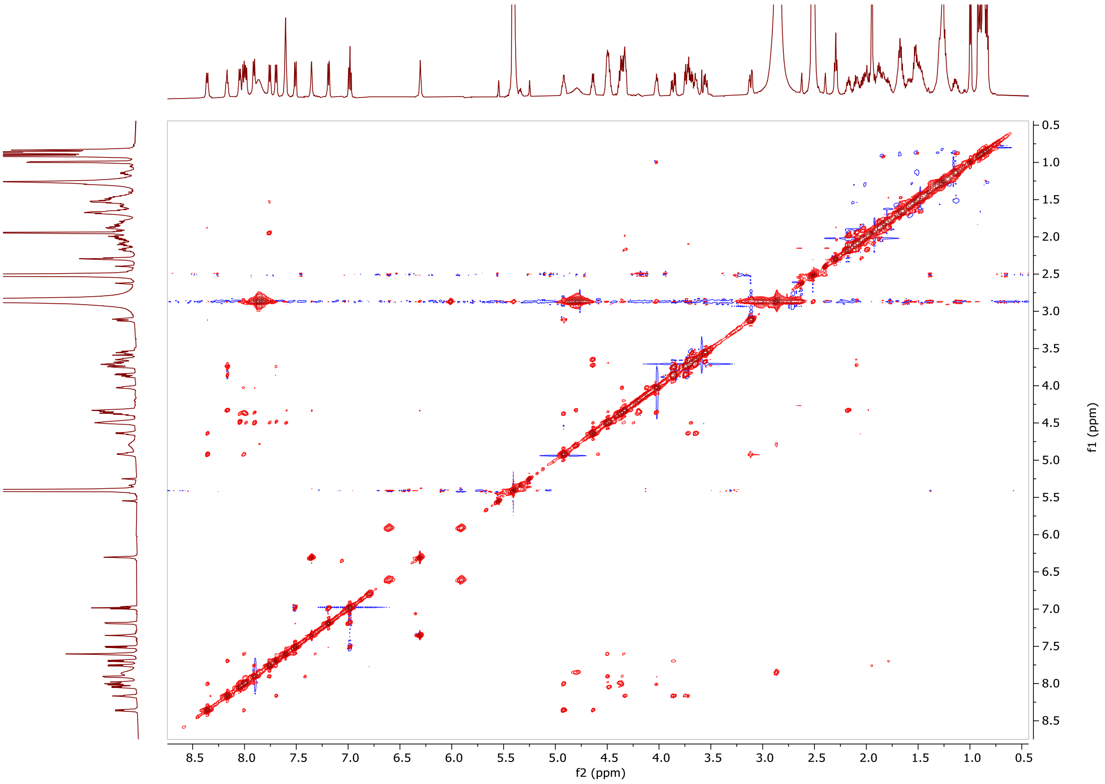

Figure 45: NOESY spectrum of **2** recorded with a 600 ms mixing time in CD_2Cl_2:DMSO-d_6 4:1 (600 MHz). Note: the positive (blue) NOE cross peaks of some side chain protons is due to their high mobility as compared to the overall tumbling of the entire molecule, which makes the double quantum ω_2 relaxation pathway to dominate over the zero quantum ω_0 pathway.

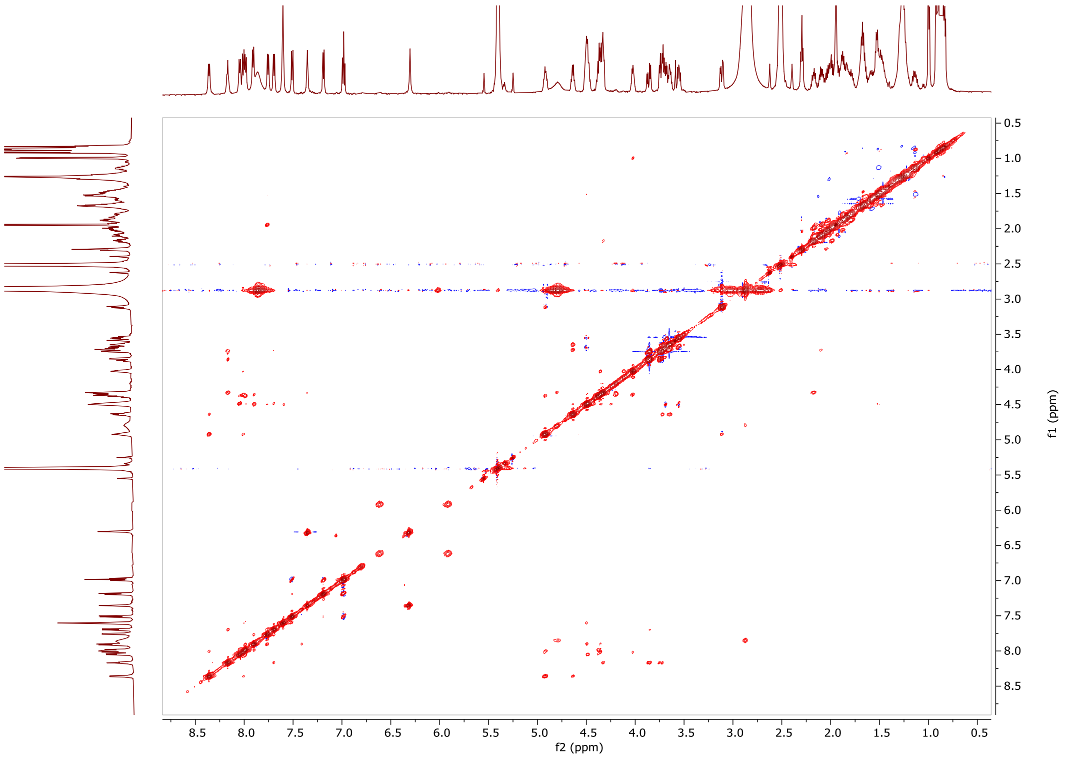

Figure 46: NOESY spectrum of **2** recorded with a 500 ms mixing time in CD_2Cl_2:DMSO-d_6 4:1 (600 MHz). Note: the positive (blue) NOE cross peaks of some side chain protons is due to their high mobility as compared to the overall tumbling of the entire molecule, which makes the double quantum ω_2 relaxation pathway to dominate over the zero quantum ω_0 pathway.

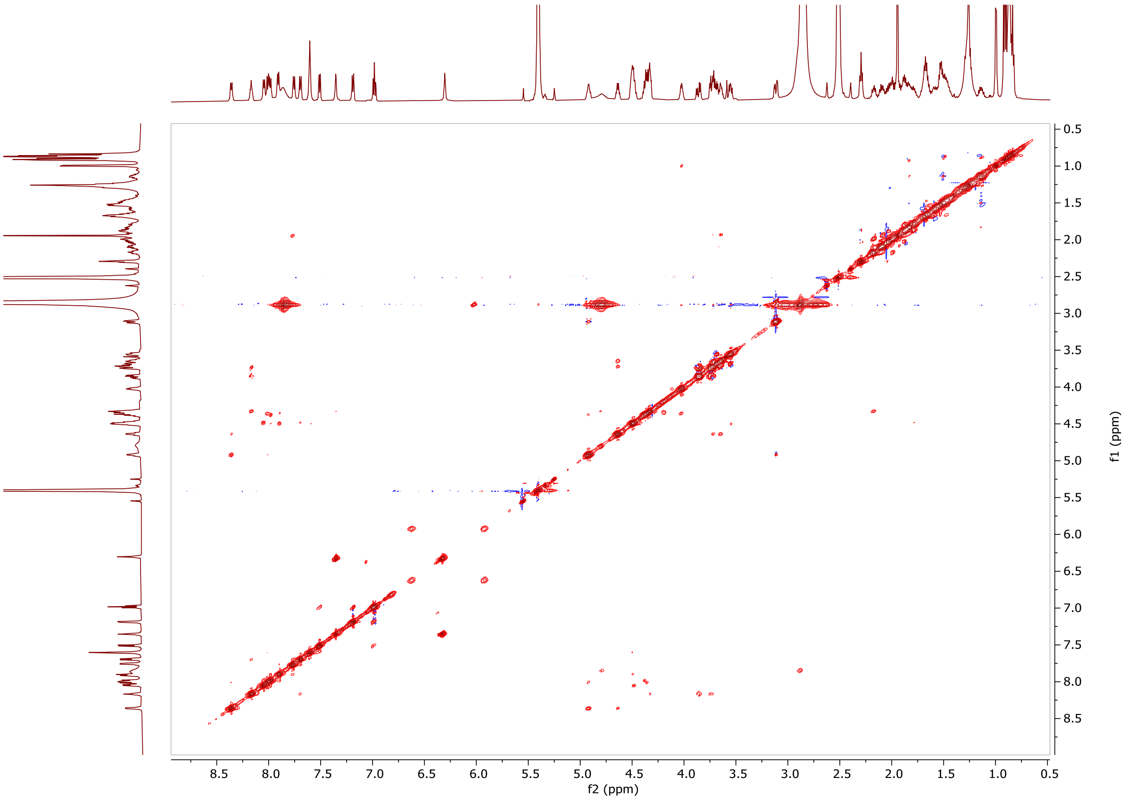

Figure 47: NOESY spectrum of **2** recorded with a 400 ms mixing time in CD_2Cl_2:DMSO-d_6 4:1 (600 MHz). Note: the positive (blue) NOE cross peaks of some side chain protons is due to their high mobility as compared to the overall tumbling of the entire molecule, which makes the double quantum ω_2 relaxation pathway to dominate over the zero quantum ω_0 pathway.

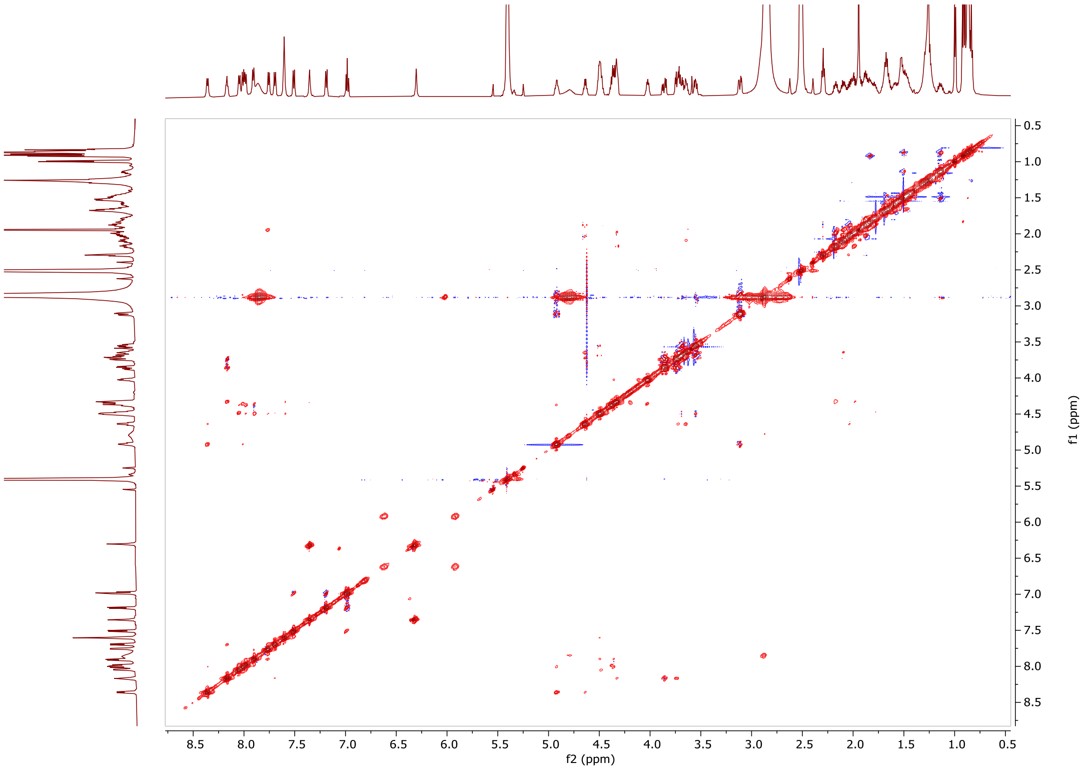

Figure 48: NOESY spectrum of **2** recorded with a 300 ms mixing time in CD_2Cl_2:DMSO-d_6 4:1 (600 MHz). Note: the positive (blue) NOE cross peaks of some side chain protons is due to their high mobility as compared to the overall tumbling of the entire molecule, which makes the double quantum ω_2 relaxation pathway to dominate over the zero quantum ω_0 pathway.

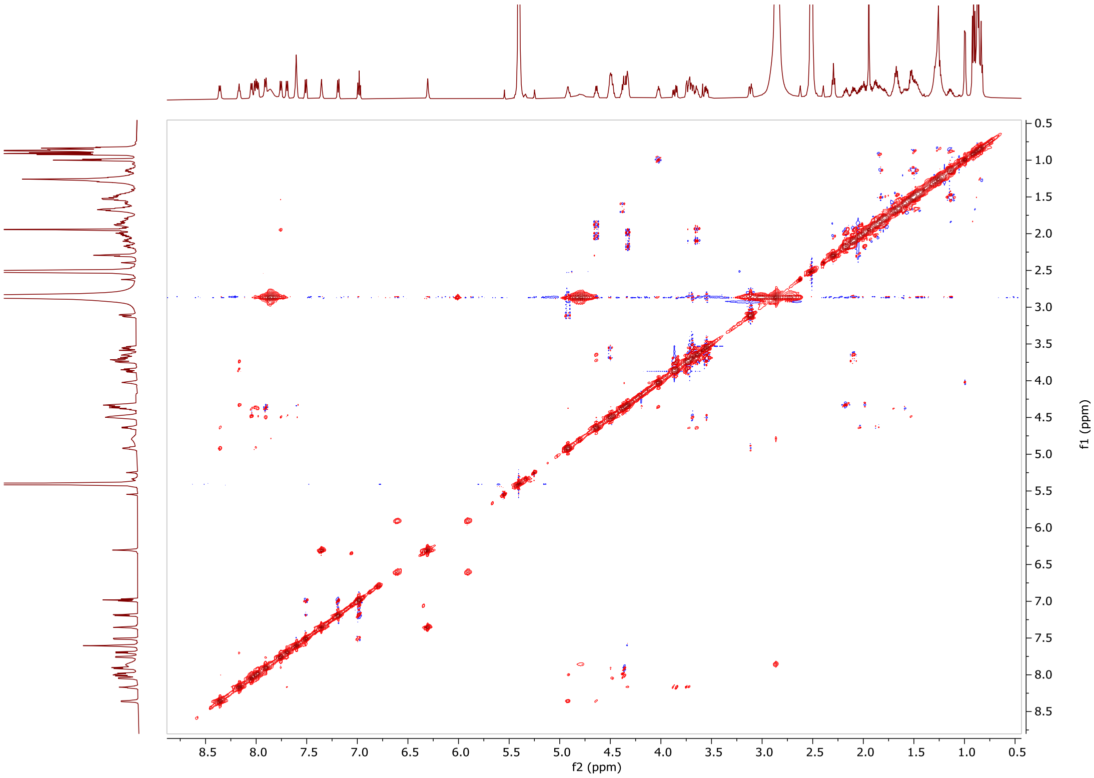

*Figure 49: NOESY spectrum of **2** recorded with a 200 ms mixing time* in CD_2Cl_2:DMSO-d_6 4:1 (600 MHz). Note: the positive (blue) NOE cross peaks of some side chain protons is due to their high mobility as compared to the overall tumbling of the entire molecule, which makes the double quantum ω_2 relaxation pathway to dominate over the zero quantum ω_0 pathway.

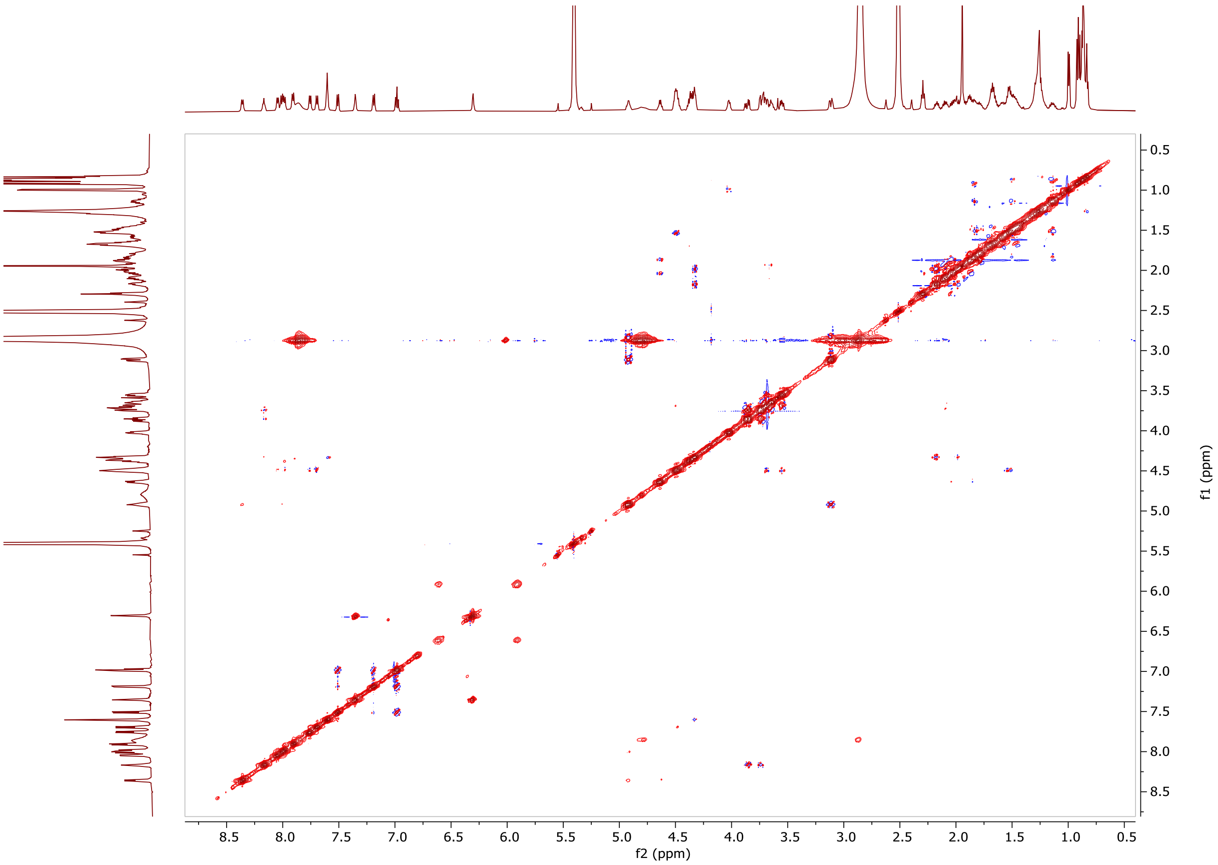

*Figure 50: NOESY spectrum of **2** recorded with a 100 ms mixing time* in CD_2Cl_2:DMSO-d_6 4:1 (600 MHz). Note: the positive (blue) NOE cross peaks of some side chain protons is due to their high mobility as compared to the overall tumbling of the entire molecule, which makes the double quantum ω_2 relaxation pathway to dominate over the zero quantum ω_0 pathway.

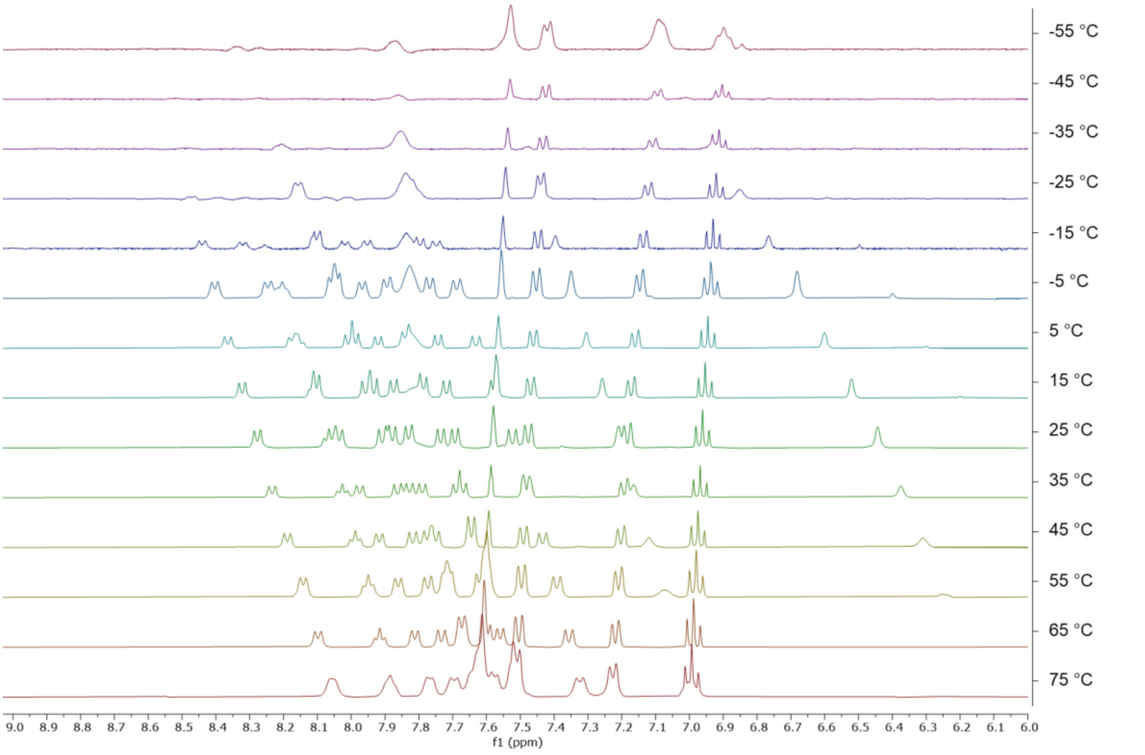

Figure 51: Stacked ^{1}*H-NMR spectra of peptide* ***1*** *following the chemical shift change of amide NH protons. Temperature range from -55°C to +75°C in increments of 10°C.*

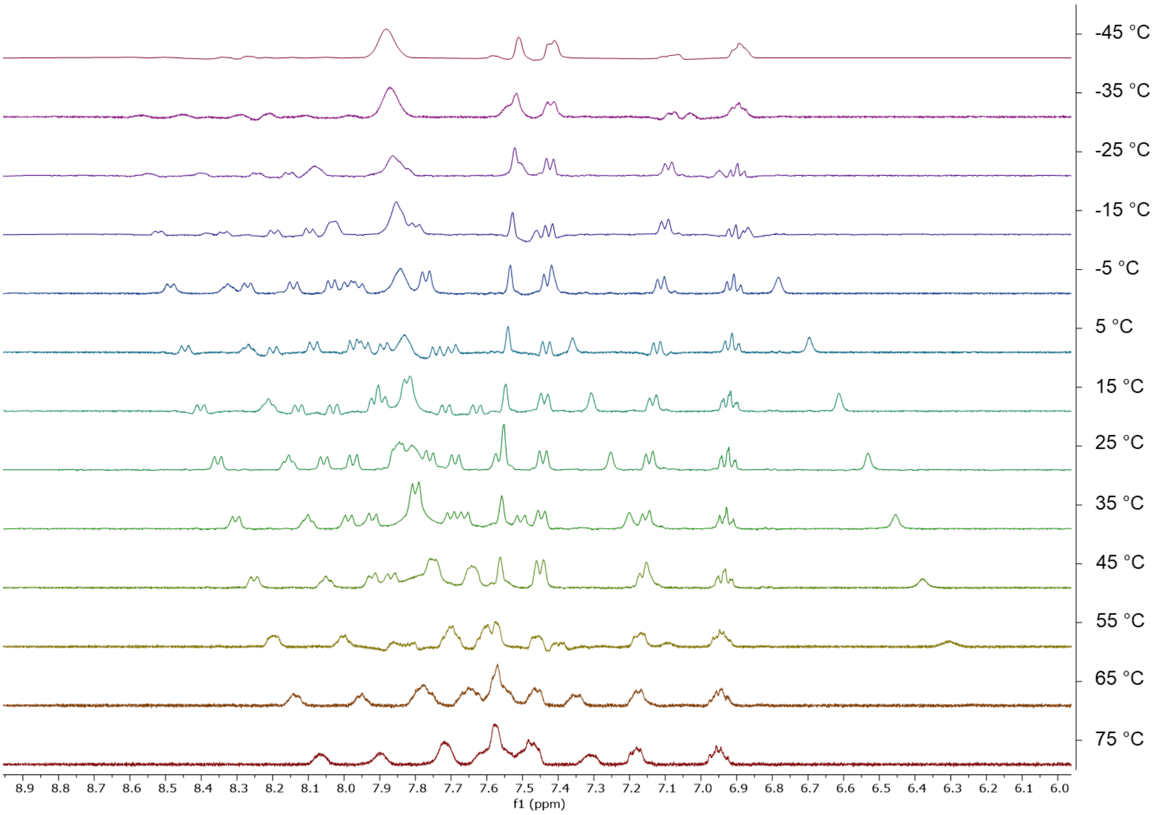

Figure 52: Stacked ^{1}H-NMR spectra of peptide **2** following the chemical shift change of amide NH protons. Temperature range from -45°C to +75°C in increments of 10°C.

9 HRMS Spectrograms

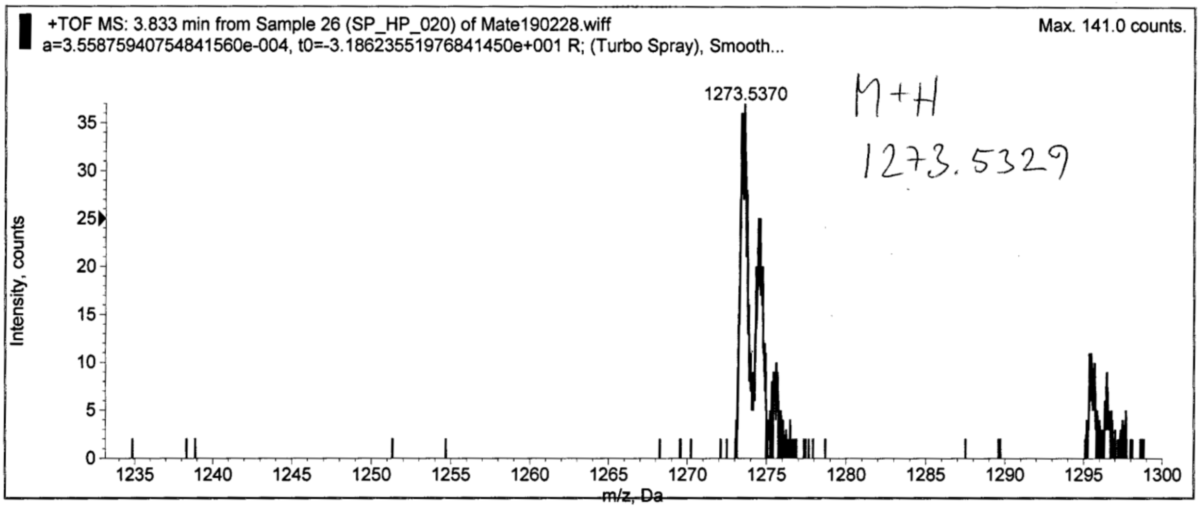

Figure 53: HRMS spectrogram of **1**. $[M+H]^+$ calculated for $C_{53}H_{85}IN_{12}O_{16}$: 1273.5329. Found: 1273.5370. Error: 3.22 ppm.

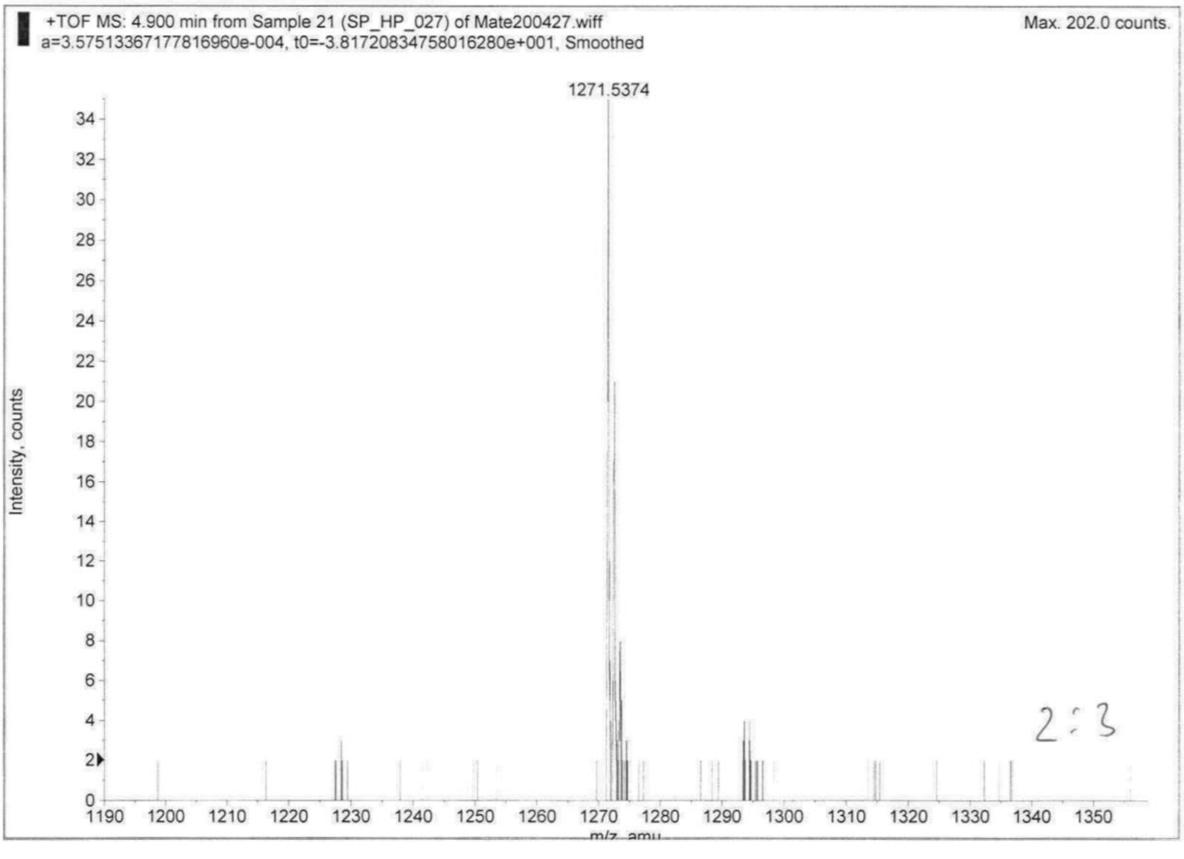

Figure 54: HRMS spectrogram of **2.** $[M+H]^+$ calculated for $C_{54}H_{87}IN_{12}O_{15}$: 1271.5537. Found: 1271.5374. Error: -12.82 ppm.

10 References

[1] C. P. Butts, C. R. Jones, E. C. Towers, J. L. Flynn, L. Appleby, N. J. Barron, *Org. Biomol. Chem.* **2011**, *9*, 177-184.

[2] H. Kessler, *Angew. Chem. Int.Ed.* **1982**, *94*, 509-520.

[3] K. Wüthrich, *NMR of proteins and nucleic acids*, **1986**.

[4] D. L. Minor, P. S. Kim, *Nature* **1994**, *367*, 660-663.

[5] S. Honda, N. Kobayashi, E. Munekata, *J. Mol. Biol.* **2000**, *295*, 269-278.

[6] N. Kobayashi, S. Honda, H. Yoshii, E. Munekata, *Biochemistry* **2000**, *39*, 6564-6571.

[7] A. N. Analytical Methods Committee, *Anal. Meth.* **2016**, *8*, 5553-5555.

[8] P. Auffinger, F. A. Hays, E. Westhof, P. S. Ho, *Proc. Natl. Acad. Sci (USA)* **2004**, *101*, 16789.

[9] H. Andersson, E. Danelius, P. Jarvoll, S. Niebling, A. J. Hughes, S. Westenhoff, U. Brath, M. Erdélyi, *ACS Omega* **2017**, *2*, 508-516.

[10] (a) H. Kessler, C. Griesinger, J. Lautz, A. Mueller, W. F. Van Gunsteren, H. J. C. Berendsen, *J. Am. Chem. Soc.* **1988**, *110*, 3393-3396;
(b) M. P. Williamson, in *Spectroscopic Methods and Analyses: NMR, Mass Spectrometry, and Metalloprotein Techniques* (Eds.: C. Jones, B. Mulloy, A. H. Thomas), Humana Press, Totowa, NJ, **1993**, pp. 69-85.

[11] N. Nevins, D. Cicero, J. P. Snyder, *J. Org. Chem.* **1999**, *64*, 3979-3986.

[12] M. A. Jiménez, in *Protein Design: Methods and Applications* (Ed.: V. Köhler), Springer New York, New York, NY, **2014**, pp. 15-52.

[13] G. Cornilescu, J. L. Marquardt, M. Ottiger, A. Bax, *J. Am. Chem. Soc.* **1998**, *120*, 6836-6837.

[14] A. Marx, C. Thiele, *Chem. Eur. J.* **2009**, *15*, 254-260.

[15] Y. Zhao, D. G. Truhlar, *Theor. Chem. Acc.* **2008**, *120*, 215.

[16] a) S. Grimme, J. Antony, S. Ehrlich, H. Krieg, *J. Chem. Phys.* **2010**, *132*, 154104; b) S. Grimme, *J. Comput. Chem.* **2006**, *27*, 1787.

[17] a) F. Weigend, R. Ahlrichs, *Phys. Chem. Chem. Phys.* **2005**, *7*, 3297; b) D. Rappoport, F. Furche, *J. Chem. Phys.* **2010**, *133*, 134105; c) E. Engelage, D. Reinhard, S. M. Huber, *Chem. Eur. J.* **2020**, *26*, 3843.
